# Pathogenicity of Mediator Complex Subunit 27 (*MED27*) in a Neurodevelopmental Disorder with Cerebellar Atrophy

**DOI:** 10.1002/advs.202505535

**Published:** 2025-09-29

**Authors:** Nuermila Yiliyaer, Xiaocheng Li, Tianyu Guo, Haiying Zhou, Lihai Gong, Luowei Yuan, Yang Fu, Yulong Qiao, Ying Lam Lui, Nuo Chen, Pengfei Lin, Hoi Hung Cheung, Ho Ko, Linyan Meng, Xiao Chen, Yong Lei, Kin Ming Kwan, Huating Wang, Shen Gu

**Affiliations:** ^1^ School of Biomedical Sciences Faculty of Medicine The Chinese University of Hong Kong (CUHK) Hong Kong SAR China; ^2^ Gerald Choa Neuroscience Institute CUHK Hong Kong SAR China; ^3^ School of Medicine CUHK (Shenzhen) Shenzhen Guangdong 518172 China; ^4^ Department of Orthopaedics and Traumatology Li Ka Shing Institute of Health Sciences CUHK Hong Kong SAR China; ^5^ Center for Neuromusculoskeletal Restorative Medicine Limited Hong Kong Science Park Hong Kong SAR China; ^6^ School of Life Sciences CUHK Hong Kong SAR China; ^7^ Department of Neurology and Research Institute of Neuromuscular and Neurodegenerative Diseases Qilu Hospital of Shandong University Jinan Shandong 250012 China; ^8^ Division of Neurology Department of Medicine and Therapeutics & Li Ka Shing Institute of Health Sciences Faculty of Medicine CUHK Shatin Hong Kong China; ^9^ Department of Molecular and Human Genetics Baylor College of Medicine Houston TX 77030 USA; ^10^ Baylor Genetics Houston TX 77021 USA; ^11^ Department of Sports Medicine and Orthopedic Surgery of The Second Affiliated Hospital and Liangzhu Laboratory Zhejiang University School of Medicine Hangzhou 310000 China; ^12^ Dr. Li Dak Sum‐Yip Yio Chin Center for Stem Cells and Regenerative Medicine Zhejiang University School of Medicine Hangzhou Zhejiang 310058 China; ^13^ Center for Cell and Developmental Biology CUHK Hong Kong SAR China; ^14^ State Key Laboratory of Agrobiotechnology (CUHK) Hong Kong SAR China; ^15^ CUHK Shenzhen Research Institute Shenzhen 518063 China; ^16^ Key Laboratory for Regenerative Medicine Ministry of Education School of Biomedical Sciences Faculty of Medicine CUHK Hong Kong SAR China; ^17^ Kunming Institute of Zoology‐The Chinese University of Hong Kong (KIZ‐CUHK) Joint Laboratory of Bioresources and Molecular Research of Common Diseases Hong Kong SAR China; ^18^ CUHK‐GIBH CAS Joint Research Laboratory on Stem Cell Regenerative Medicine CUHK Hong Kong SAR China

**Keywords:** MED27, Mediator complex, neurodevelopmental disorder, pathogenic mechanism

## Abstract

Neurodevelopmental disorders (NDDs) affect brain function and development, with 90% lacking approved treatments. Understanding their pathogenic mechanisms is critical for developing precision gene therapies. An autosomal recessive NDD associated with variants in the Mediator complex subunit 27 (*MED27*) gene is previously identified. The Mediator complex is essential for transcription initiation by bridging transcription factors (TFs) at enhancers to RNA polymerase II at promoters. All patients with *MED27* variants exhibit cerebellar hypoplasia or atrophy, underscoring the cerebellum's heightened vulnerability to MED27 dysfunction. To investigate the disease mechanisms, in vitro stem cells carrying patient‐specific *MED27* variants and in vivo mouse models with *Med27* loss‐of‐function (LoF) are generated. These preclinical models recapitulate key patient phenotypes, including progressive cerebellar atrophy and motor deficits. Molecular analyses reveal that mutant MED27 destabilizes the Mediator complex, impairing its chromatin occupancy and altering chromatin interactions. Comprehensive transcriptomic profiling, including single‐cell resolution spatial transcriptomics, identifies dysregulation of downstream targets regulated by *MED27*, such as critical master regulatory TFs involved in neurogenesis and cerebellar development. This study elucidates a partial LoF mechanism underlying *MED27*‐associated NDDs and establishes a prototype for investigating NDDs caused by pathogenic variants in Mediator subunits.

## Graphical abstract

MED27 is one of the 26 subunits in the human Mediator complex (MED). Neurodevelopmental disorder‐causing MED27 genetic variants induce instability of MED, leading to disrupted DNA occupancy, altered chromatin interaction, and subsequent transcriptional dysregulation of critical downstream genes, including master regulatory transcription factors essential for early neurogenesis and cerebellar development.

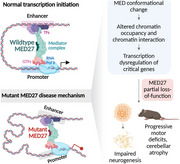

## 1 Introduction

The Mediator complex (MED) is a highly conserved, large protein assembly comprising 26 subunits in humans.^[^ ^1^, ^2^ ^]^ Together with RNA polymerase II (Pol II) and several general transcription factors (TFs), it initiates the transcription of most protein‐coding genes and many noncoding RNAs.^[^ ^3^ ^]^ Besides being a general transcriptional co‐activator, MED is also strictly required for the functionality of cell type‐specific gene regulatory circuits.^[^ ^4^ ^]^ By bridging TFs bound at enhancers to the RNA Pol II transcription machinery at promoters, its conformational flexibility allows it to integrate and transmit regulatory signals between enhancers and promoters. MED occupies promoters in cell type‐specific patterns, resulting in cell type‐specific modulation of gene expression.^[^ ^5^, ^6^ ^]^ Furthermore, different MED subunits bind to different TFs, and these subunit‐specific TF–Mediator interactions activate distinct TF‐specific gene sets.^[^ ^3^ ^]^ Studies on individual MED subunits have shown that each subunit influences specific cellular or developmental processes. For instance, MED1 binds with KLF4, a master TF in endothelium, to activate the BMP/TGF‐β pathway genes in pulmonary arterial endothelial cells,^[^ ^7^ ^]^ while MED20 interacts with the TF C/EBPβ to promote transcription of the central adipogenic factor PPARγ in adipocytes.^[^ ^8^ ^]^

Several MED subunit genes have been implicated in monogenic diseases, including *MED11* (Online Mendelian Inheritance in Man [OMIM] #620327), *MED17* (OMIM #613668), *MED23* (OMIM #614249), *MED25* (OMIM #616449), and *MED27* (OMIM #619286, first associated with a single‐gene defect in our previous study^[^ ^9^ ^]^). In addition, subunits from the Mediator kinase module, including *MED12*, *MED12L*, *MED13*, *MED13L*, and *CDK8*, have been linked to Mendelian diseases (OMIM# 301068/ 309520/ 300895/ 305450, 618872, 618009, 616789, and 618748, respectively). Notably, all these patients presented with neurodevelopmental disorders (NDDs), albeit with variable neurological defects and brain structural anomalies. Collectively, these conditions are referred to as “neuro‐MEDopathies”.^[^ ^10^, ^11^ ^]^ For these MED subunits, while the neurological features in patients suggest their critical roles in brain development, the pathogenicity mechanisms remain largely unexplored. Furthermore, each subunit may regulate distinct gene sets, as evidenced by the variable brain structural anomalies observed in patients with different subunit‐specific disease‐causing variants.

In our recent study, we identified *MED27* as an autosomal recessive NDD‐causing gene.^[^ ^9^ ^]^ Through clinical exome sequencing, we identified homozygous or compound heterozygous variants in *MED27* in 16 similarly affected patients with NDD from 11 families.^[^ ^9^ ^]^ Of the 11 unique variants identified, three were frameshift (FS) and one involved a canonical splice site, indicating a loss‐of‐function (LoF) of MED27; the remaining were highly conserved missense changes predicted to be deleterious by multiple in silico programs. *MED27* patients shared overlapping clinical presentations, including intellectual disability, developmental delay, central hypotonia, distal limb spasticity/dystonic movement, delayed motor/speech development, and cerebellar hypoplasia observed via brain magnetic resonance imaging (MRI). In a subsequent study^[^ ^11^ ^]^ describing 57 affected individuals (including the 16 patients from our initial report) with biallelic *MED27* variants from 30 unrelated families, the phenotypic NDD manifestations spanned a broad continuum. However, brain MRI consistently revealed cerebellar hypoplasia in all patients, with progressive cerebellar atrophy observed in those who underwent follow‐up MRI scans. The prominent cerebellar phenotype observed in patients highlighted the cerebellum's vulnerability to alterations in *MED27*.

Currently, there are few published studies on MED27, and information on its functional characterization during development is nearly absent. Understanding the pathogenicity mechanisms (i.e., how patient‐specific genetic variants cause disease) is critical for developing precision therapies for treatment. This study aimed to uncover the pathogenicity mechanisms of this syndrome. To achieve this, we generated both in vitro cellular and in vivo mouse models to mimic patients’ genotypes and recapitulate their clinical presentations. We investigated the molecular, neuropathological, and behavioral abnormalities in these preclinical models. In addition to elucidating the disease's pathogenicity, our study also underscored the essential roles of MED27 during embryogenesis and cerebellar development.

## 2 Results

### 2.1 Disturbed Spontaneous Differentiation in Human Embryonic Stem Cells (hESCs) with Patient‐Specific *MED27* Variant

**Figure 1:**
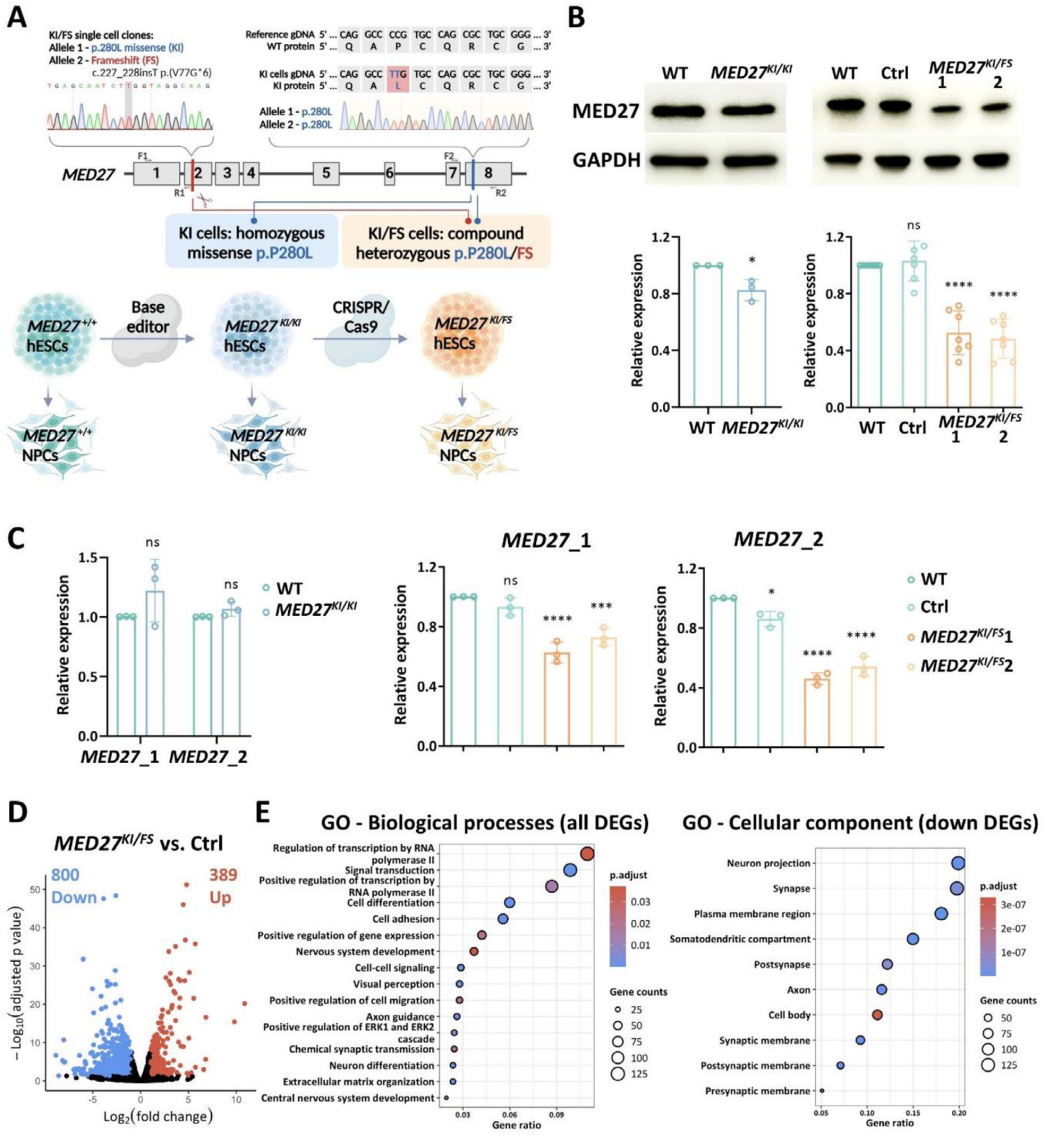
Generation of human stem cells with patient‐specific *MED27* variants. A) Human embryonic stem cells (hESCs) mutant clones with homozygous missense (*MED27^KI^* ^/^ *^KI^*) or compound heterozygous (*MED27^KI^* ^/^ *^FS^*) variants were introduced by genome editing. hESCs were further differentiated into neuronal progenitor cells (NPCs) for characterization. Two primer pairs used for qPCR of *MED27* mRNA levels are indicated by F1/R1 and F2/R2. KI, knock‐in; FS, frameshift; gDNA, genomic DNA; WT, wildtype. Created with BioRender.com. B) Representative Western blot images and quantifications of MED27 protein expression levels in *MED27^KI^* ^/^ *^KI^* and *MED27^KI^* ^/^ *^FS^* hESCs compared to the WT and control hESCs. GAPDH was used as an endogenous control. Ctrl, non‐edited control cells underwent identical genomic editing procedures; *MED27^KI^* ^/^ *^FS^* 1 and 2, two independent edited clones with desired genotype. C) qPCR results illustrating *MED27* mRNA levels in *MED27^KI^* ^/^ *^KI^* and *MED27^KI^* ^/^ *^FS^* hESCs compared to the WT and control hESCs. *MED27*_1 and *MED27*_2 are qPCR results from two different primer pairs, one before and the other after the CRISPR/Cas9 cut site (indicated by the scissor in Figure 1A). D) Volcano plot showing bulk RNA‐sequencing result comparing *MED27^KI^* ^/^ *^FS^* and control hESCs’ transcriptomic profiles. 800 downregulated differentially expression genes (DEGs) and 389 upregulated DEGs were identified (|Log_2_foldchange|>1, adjusted *p* < 0.05). E) Gene ontology (GO) analysis of DEGs identified in Figure 1D. The bubble size corresponds to the number of genes each term involved. The color intensity of the bubble corresponds to adjusted *p* value. In Figure 1B,C, data were generated from at least three biological replicates. Error bars represent standard deviation (SD) of the mean. One‐way ANOVA was used for statistical analysis. *, ***, ****, and ns denote *p* < 0.05, *p* < 0.001, *p* < 0.0001, and *p* > 0.05, respectively.

To investigate the pathogenicity of the *MED27* gene, we introduced the patient‐specific missense variant p.P280L into H1 hESCs using a CRISPR/Cas9‐modified base editor system.^[^ ^12^ ^]^ The P280L variant was selected because it was the most recurrent change in our cohort^[^ ^9^ ^]^ and was associated with severe‐to‐profound phenotypes in patients.^[^ ^11^ ^]^ We successfully established hESCs with a homozygous P280L mutation (*MED27^KI^* ^/^ *^KI^* in **Figure** **1**A) and confirmed its expression via Sanger sequencing of *MED27* cDNA (Figure S1A, Supporting Information). Western blot analysis revealed a slight reduction of MED27 protein expression due to the homozygous missense change, although the mRNA level of *MED27* in mutant cells remain unaltered (Figure 1B,C), suggesting some instability of the mutant protein.

Using CRISPR/Cas9,^[^ ^13^ ^]^ we further generated hESCs containing a heterozygous FS mutation in trans with P280L (*MED27^KI^* ^/^ *^FS^* in Figure 1A) to mimic the genotypes of patients harboring compound heterozygous FS and missense changes. Sanger sequencing of *MED27* cDNA in *MED27^KI^* ^/^ *^FS^* cells confirmed that only the non‐FS allele was expressed (Figure S1A, Supporting Information), indicating that the mRNA transcribed from the FS allele underwent nonsense‐mediated decay. This was further supported by an ≈50% reduction in MED27 protein and mRNA levels in *MED27^KI^* ^/^ *^FS^* hESCs compared to wildtype (WT) or non‐edited hESCs (control cells subjected to identical experimental procedures without *MED27* editing) (Figure 1B,C). Immunofluorescence (IF) staining of OCT4 and NANOG validated the pluripotency of all the mutant and control clones (Figure S1B, Supporting Information).

Since MED primarily functions as a general transcriptional co‐activator, we first examined the transcriptomic profiles of these hESCs. Bulk RNA‐sequencing (RNA‐seq) comparing *MED27^KI^* ^/^ *^FS^* to control cells identified 1189 differentially expressed genes (DEGs), including 800 downregulated and 389 upregulated genes (Figure 1D). Gene ontology (GO) analysis on all DEGs revealed top biological processes involving Pol II‐regulated transcription, consistent with the known functions of MED27. Notably, the top biological processes also included several terms related to nervous system development and neuron differentiation. Further analysis of the downregulated DEGs showed that 80% of the top ten cellular component terms were neuron‐related, suggesting transcriptional impairment of genes involved in neuronal processes due to *MED27* mutations (Figure 1E). To further validate the preferential transcriptional dysregulation of the neuronal lineage, mutant and control hESCs were subjected to spontaneous differentiation to generate embryoid bodies (EBs) containing the three germ layers—endoderm, mesoderm, and ectoderm. Subsequently, the expression levels of selected marker genes representing each germ layer^[^ ^14^, ^15^, ^16^ ^]^ were analyzed by reverse transcription‐quantitative polymerase chain reaction (RT‐qPCR). Corroborating with RNA‐seq results, ectodermal marker genes (*FOXG1*, *OTX2*, *PAX6*) were significantly reduced in mutant cells derived from EBs, while endodermal (*SOX17*, *FOXA2*, *GATA4*) and mesodermal markers (GSC, *TBXT*, *CDX2*) remained unaltered or increased. This result is consistent with impaired neuronal lineage differentiation caused by *MED27* mutations (Figure S1C, Supporting Information).

### 2.2 Altered Chromatin Occupancy and Transformed Genome Organization in *MED27* Mutant Neuronal Progenitor Cells (NPCs)

**Figure 2:**
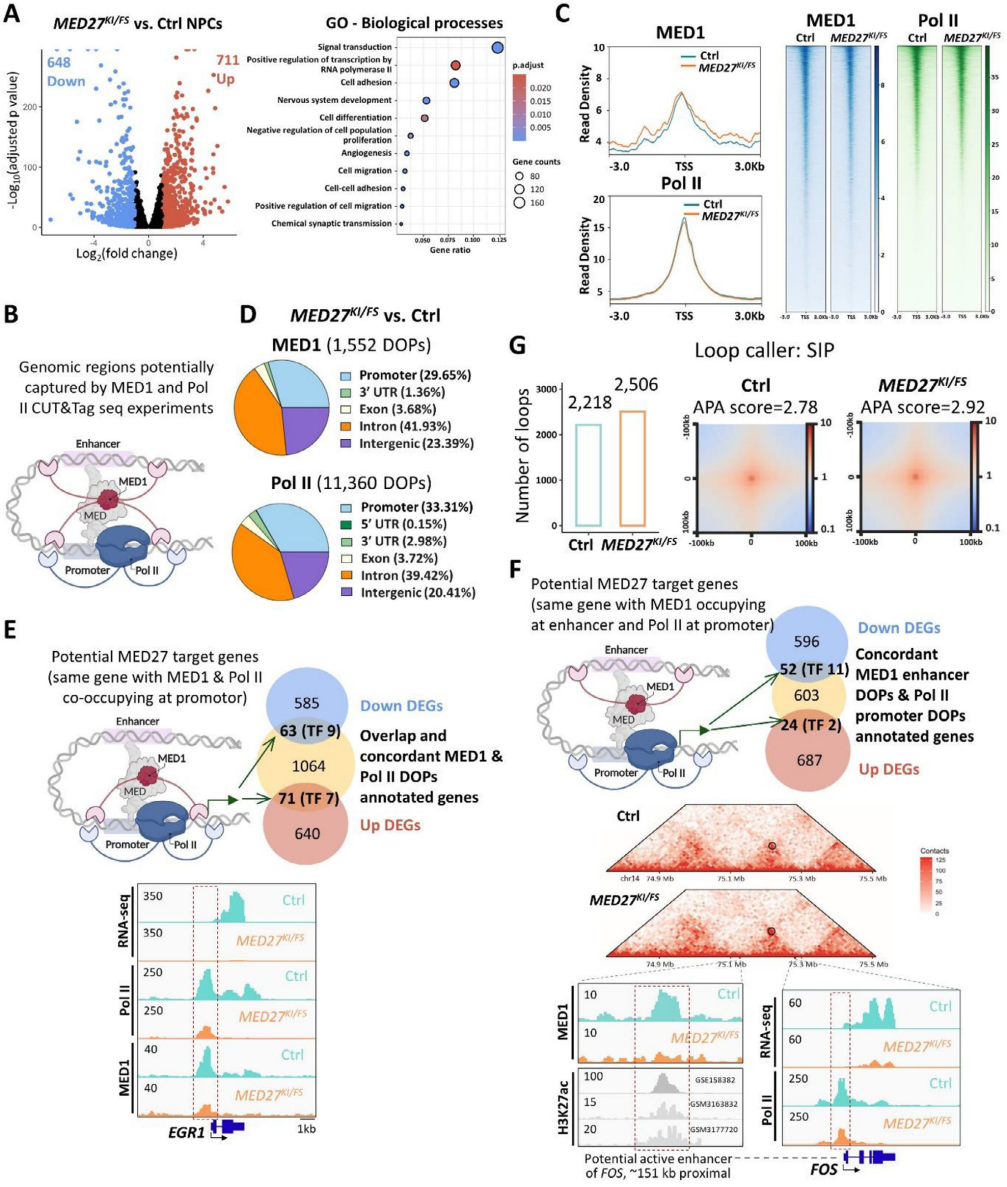
Altered DNA occupancy and transformed genome organization in *MED27* mutant neuronal progenitor cells (NPCs). A) Volcano plot (left) showing bulk RNA‐sequencing result comparing *MED27^KI^* ^/^ *^FS^* and control NPC's transcriptomic profiles. 648 downregulated DEGs and 711 upregulated DEGs were identified (|Log_2_foldchange |>1, adjusted *p* < 0.05). Top biological processes involved by GO analysis of all the DEGs are shown on the right. The bubble size corresponds to the number of genes each term involved. The color intensity of the bubble corresponds to adjusted *p*‐value. B) Illustration of the genomic regions potentially captured by MED1 and Pol II CUT&Tag seq experiments. Note that within the transcription machinery, Pol II interacts with the promoter region, while MED1 could interact with both active enhancer and promoter regions. Created with BioRender.com. C) Meta‐analysis and heatmap of MED1 and Pol II CUT&Tag read density around transcription start site (TSS) within a ±3 kb window, respectively. D) Genomic distribution of MED1 and Pol II differential occupancy peaks (DOPs) between mutant and control NPCs, respectively. E) Venn diagram (up) showing overlapped DEGs with concordant MED1 and Pol II DOPs at the promoter regions. TF, transcription factors identified among the overlapped genes. (Down) Bulk RNA‐seq read counts of *EGR1* and occupancy profiles of Pol II and MED1 at *EGR1*’s promoter. F) Venn diagram (top) showing overlapped DEGs with MED1 DOPs at active enhancers and Pol II DOPs at promoters. Hi‐C heatmap (middle) showing the chromatin loop connecting the *FOS* potential enhancer to the *FOS* promoter (coordinates in GRCh38/hg38). (Bottom) Bulk RNA‐seq read counts of *FOS* and occupancy profiles of Pol II at the *FOS* promoter, as well as MED1 at the potential active enhancer. The active enhancer region has positive H3K27ac occupancy across three NPC datasets (see also Figure S2B, Supporting Information). G) Bar chats illustrating loop counts and heatmap showing loop strengths (loopability) for control and mutant NPCs analyzed by loop caller SIP. Aggregate peak analysis (APA) scores were calculated to demonstrate enrichment of loops for each sample.

Since MED initiates transcription by bridging TFs bound at enhancers to Pol II at promoters, we hypothesized that mutant MED27 induces dysregulated transcription of target genes by reshaping the genomic occupancy profile of MED and transforming chromatin interactions. To test this hypothesis, we used NPCs differentiated from hESC, as NPCs give rise to nearly all neuronal and glial cells that populate the central nervous system (CNS).^[^ ^17^ ^]^ The differentiated cells showed morphological consistency with NPCs and expressed the NPC markers NESTIN and PAX6 (Figure S1D, Supporting Information). Similarly to hESCs, *MED27^KI^* ^/^ *^FS^* NPCs exhibited approximately 50% reductions in MED27 protein and mRNA levels compared to control NPCs (Figure S1E,F, Supporting Information). To analyze transcriptomic changes, we performed bulk RNA‐seq on mutant and control NPCs, identifying 648 downregulated and 711 upregulated DEGs. GO analysis of these DEGs revealed significant enrichment for pathways involved in Pol II transcription regulation and neurodevelopmental processes, consistent with the expected dysregulated pathways caused by patient‐specific *MED27* mutations (**Figure** **2**A).

To investigate the genomic occupancy profiles of MED27, we attempted chromatin immunoprecipitation sequencing (ChIP‐seq) and cleavage under targets and tagmentation (CUT&Tag) sequencing but were unsuccessful using all commercially available MED27 antibodies. This limitation likely stems from MED27's embedding within the MED complex and its entanglement with other subunits,^[^ ^1^ ^]^ making it inaccessible for antibody binding. Since MED27 always functions as part of MED, we analyzed MED as a whole instead. Specifically, we conducted CUT&Tag seq for MED1, the most commonly utilized subunit to represent MED occupancy profiles,^[^ ^18^, ^19^ ^]^ as well as Pol II CUT&Tag seq to identify transcriptional machinery occupancy sites. Pol II occupancy profiles were analyzed at promoter regions, while MED1 occupancy was analyzed at both promoter and active enhancer regions (Figure 2B).

In *MED27* mutant versus control NPCs, we identified 1552 and 11360 differential occupancy peaks (DOPs) for MED1 and Pol II, respectively (Figure 2C,D). Analysis of common and concordant DOPs between the two factors annotated 1198 genes, which overlapped with 63 downregulated and 71 upregulated DEGs identified from RNA‐seq (down DEG with down DOPs for both MED1 and Pol II comparing mutant NPCs to the control, or up DEG with up DOPs). These overlapping genes are potential downstream targets of MED27, regulated at their promoter regions (Figure 2E). Among the 63 downregulated DEGs, the gene with the highest mRNA level reduction in mutant NPCs compared to controls was *EGR1* (Early Growth Response 1) (Figure 2E and Table S1, Supporting Information). RT‐qPCR and CUT&Tag qPCR confirmed reduced *EGR1* expression and diminished MED1/Pol II occupancy at its promoter in mutant NPCs (Figure S2A, Supporting Information). As a prototype immediate early response gene (IEG), EGR1 is a critical transcriptional regulator involved in brain development, learning, and long‐term neuronal plasticity.^[^ ^20^ ^]^

To verify the above findings, we conducted MED23 CUT&Tag seq and performed a similar analysis as was done using MED1 data. MED23 is a subunit from the tail module of the MED complex, which has a more flexible and dynamic structure.^[^ ^3^ ^]^ Consistent with this, the genomic occupancy of MED23 was altered to a much greater degree in mutant cells compared to MED1 (a middle module subunit). Specifically, we identified 1552 DOPs for MED1 in mutant versus control NPCs (Figure 2D), while for MED23, we identified 15468 DOPs (Figure S2C, Supporting Information). To investigate further, we performed an integrative analysis combining bulk RNA‐seq data with MED23 and Pol II CUT&Tag seq data, following the same approach used for MED1 and Pol II. This analysis identified 4703 genes with common and concordant DOPs between MED23 and Pol II. These genes overlapped with 105 downregulated and 258 upregulated DEGs identified from RNA‐seq (i.e., downregulated DEG with decreased DOPs for both MED23 and Pol II in mutants compared to controls, or upregulated DEG with increased DOPs) (Figure S2D, Supporting Information). Among the 105 downregulated DEGs, the gene with the highest mRNA level reduction in mutant NPCs compared to controls was *EGR1*, consistent with the analysis results from MED1 (Figure S2D and Table S1, Supporting Information). Furthermore, the DEGs identified using MED1 data were highly consistent with those identified using MED23 data, with 66.7% downregulated DEGs (42 out of 63) and 98.6% upregulated DEGs (70 out of 71) identified using MED1 also found using MED23 data (Figure S2E, Supporting Information). These findings demonstrate the robustness of our analysis pipeline and further support *EGR1* as the top downstream target in the context of mutant MED27.

In addition, we investigated potential downstream target genes with MED1 DOPs at active enhancers and Pol II DOPs at promoters (Figure 2F). Enhancer regions in NPCs were designated using H3K27ac, an active enhancer‐defining histone marker^[^ ^21^, ^22^, ^23^ ^]^ (Figure S2B, Supporting Information). This analysis identified 52 downregulated and 24 upregulated DEGs with concordant MED1 DOPs at active enhancers and Pol II DOPs at promoters (Figure 2F). Among these, *FOS*, which encodes the c‐Fos protein, was one of the most significantly downregulated TFs (Figure 2F; Table S1, Supporting Information). Similar to *EGR1*, *FOS* is an IEG with well‐established roles in neuronal activity and brain development.^[^ ^24^ ^]^ Dysregulation of both *EGR1* and *FOS* has been reported in MED23‐deficient ESCs and NDD patients with *MED23* or *MED12* pathogenic variants.^[^ ^25^, ^26^, ^27^, ^28^ ^]^

Eukaryotic genome organization operates at three levels: (i) the linear genome, defined by nucleotide sequences; (ii) the epigenome, defined by DNA methylation and histone modifications; and (iii) the three‐dimensional (3D) genomic structure that organizes chromatin into distinct nuclear territories. The 3D spatial organization can be analyzed and mapped using high‐throughput chromosome conformation capture (Hi‐C).^[^ ^29^ ^]^ Given MED's bridging effect of enhancers and promoters, we hypothesized that MED‐containing mutant MED27 could alter chromatin interactions. To test this hypothesis, we performed Hi‐C analysis of control and mutant NPCs, obtaining 248 million uniquely mapped contacts, which allowed us to visualize chromatin looping events at a 10‐kb resolution. Using the SIP algorithm,^[^ ^30^ ^]^ we identified 2218 chromatin loops in control NPCs and 2506 loops in mutant NPCs (Figure 2G), indicating an increase in the number of loops due to the mutant MED27. Furthermore, aggregated peak analysis (APA) revealed an enhancement in interaction strength, with values increasing from 2.78 in the control to 2.92 in the mutant NPCs, suggesting increased chromatin interactions. In addition, we observed changes in the chromatin loop landscape induced by mutant MED27, with 44.1% of loops in control NPCs and 50.5% in mutant NPCs showing alterations (Figure S2F, Supporting Information). Specifically, we identified 310 control‐specific loops and 588 mutant‐specific loops, which accounted for 8.9% and 16.9% of all merged loops, respectively (Figure S2G, Supporting Information). These findings align with previous reports showing that cells with Pol II depletion exhibited higher loop counts and more prominent loops,^[^ ^31^, ^32^ ^]^ suggesting a potential LoF in the transcriptional machinery due to patient‐specific *MED27* variants. To validate these findings, we employed another loop‐calling algorithm, Mustache,^[^ ^33^ ^]^ which produced consistent results (Figure S2I, Supporting Information).

We also examined the genes involved in the 310 control‐specific loops (lost in the mutant) and 588 mutant‐specific loops (gained in the mutant) and performed GO analysis. For genes contained in these loops, GO analysis revealed that the top biological processes and molecular functions were related to Pol II‐regulated transcription, consistent with the known functions of the MED complex (Figure S2H, Supporting Information). Specifically, 106 genes within 99 control‐specific loops and 161 genes within 158 mutant‐specific loops were associated with these GO terms (Table S1, Supporting Information).

Notably, a specific chromatin loop identified in our Hi‐C experiment supports the interaction between the potential *FOS* enhancer and its promoter in both control and mutant cells. As shown in Figure 2F, two anchors of this loop connect the two genomic regions, with one anchor (chr14:75130000‐75140000) in close proximity to the potential *FOS* enhancer (chr14:75126990‐75127389) and the other anchor (chr14:75300000‐75310000) near the *FOS* promoter (chr14:75278673‐75278919). This observation further supports *FOS* as a direct downstream target regulated by mutant MED27.

### 2.3 Decreased Stability of the MED Complex due to Patient‐Specific *MED27* Variants

**Figure 3:**
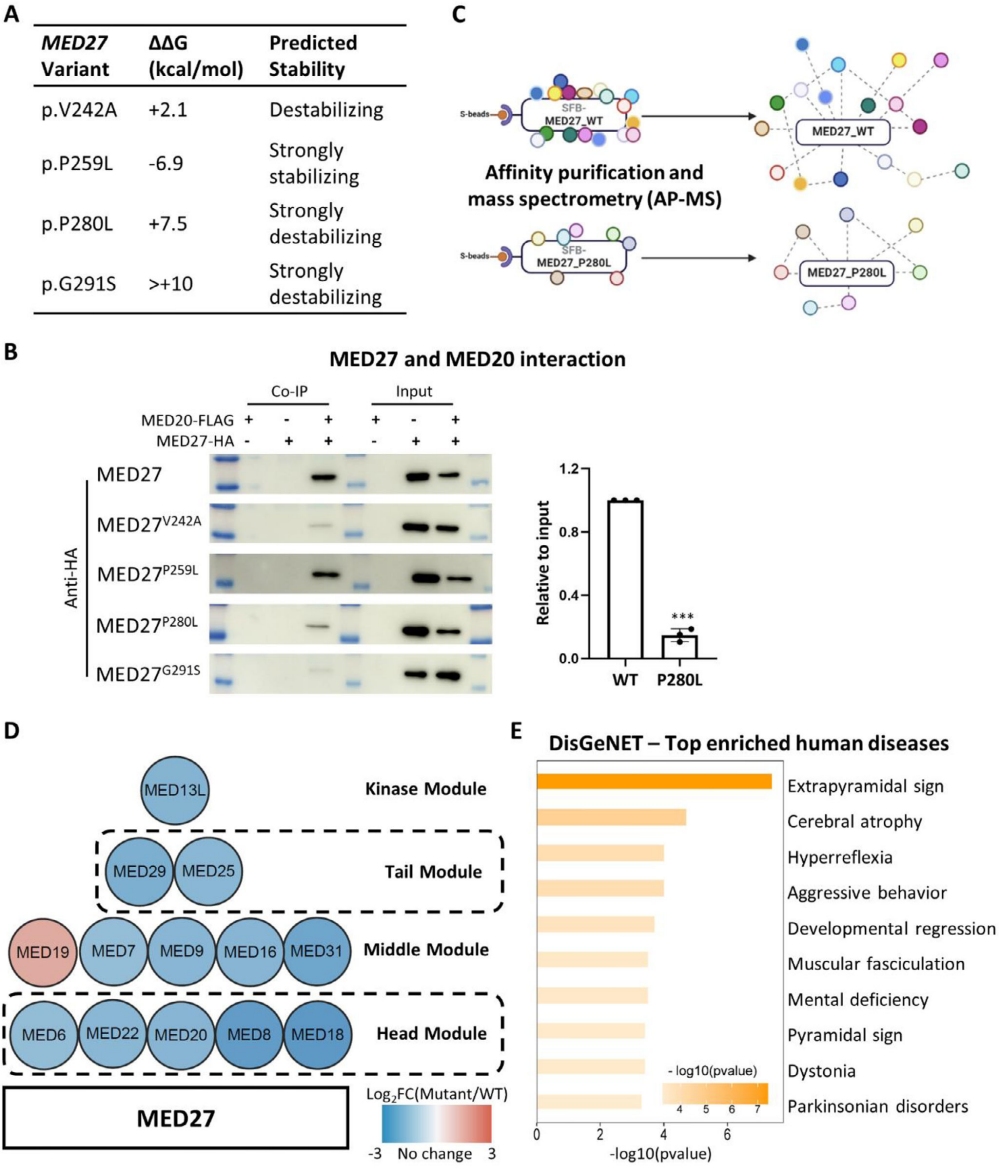
Decreased stability of the MED complex due to patient‐specific *MED27* mutations. A) Predicted changes in Gibbs free energy comparing protein complex with mutant MED27 versus WT MED27 (∆∆*G*) by in silico modeling of MED27 and its neighboring subunits. ∆∆*G* > +2 kcal mol^−1^ was defined as destabilizing. Prediction scores were adopted from ref.[11] B) Representative co‐immunoprecipitation (Co‐IP) blots on the interactions between MED27 (WT and four mutant forms) and MED20 (left). Semi‐quantitative analysis on the interacting strength between MED27 and MED20, comparing WT MED27 to mutant MED27 with the p.P280L mutation (right). Data were generated from three independent replicates. Error bars represent SD of the mean. Unpaired *t*‐test was used for statistical analysis. *** denotes *p* < 0.001. C) Illustration of the AP‐MS experiment to identify interacting proteins with WT MED27 and p.P280L mutant MED27, respectively. WT or mutant MED27 were conjugated with SFB tags (S tag, FLAG epitope tag, and streptavidin‐binding peptide tag) for affinity purification (AP). Post AP proteins were subjected to mass spectrometry (MS) to reveal the identities of the MED27 interacting proteins. D) The protein–protein interaction (PPI) map featuring MED27 as the bait protein and MED subunits as the prey proteins it interacted. The color intensity of prey proteins corresponds to Log_2_foldchange (Log_2_FC) value comparing captured protein amount by the mutant MED27 versus that by the WT MED27. E) Top 10 enriched disease terms comparing differentially interacting proteins between mutant MED27 (p.P280L) and WT MED27 by DisGeNET analysis. Disease terms with *p*‐value < 0.01, a minimum count of 3, and an enrichment factor > 1.5 are defined as significantly enriched. The top 10 enriched terms shown here are ranked by –log_10_ (*p*‐value).

As an architectural and functional bridge connecting enhancers and promoters to initiate gene transcription,^[^ ^5^ ^]^ a stable yet flexible conformation is essential for the functionality of the MED complex. Structurally, MED is divided into three modules‐head, middle, and tail.^[^ ^1^, ^2^ ^]^ Cryo‐electron microscopy structural analyses of MED have revealed that MED27 straddles the head and tail modules. The N‐terminal region of MED27 interacts with MED29, while its C‐terminal domain directly contacts several subunits from the head and tail modules, including MED20, MED22, MED28, and MED30.^[^ ^2^ ^]^ Previous in silico modeling of MED27 and its neighboring subunits predicted that nearly all patient‐specific *MED27* variants would destabilize the MED conformation^[^ ^11^ ^]^ (**Figure** **3**A). We therefore hypothesized that mutant MED27 with patient‐specific changes disrupts its interaction with neighboring subunits, inducing conformational changes of the MED complex and subsequently leading to transcriptional dysregulation of downstream genes.

To test this hypothesis, we first analyzed the interactions between WT and mutant MED27 proteins and their neighboring subunits using co‐immunoprecipitation (Co‐IP). Consistent with in silico predictions, MED27 proteins with patient‐specific p.V242A, p.P280L, and p.G291S mutations—‐all predicted to be destabilizing—‐exhibited weaker binding with MED20 and MED22. Conversely, the p.P259L mutation, predicted to be stabilizing, showed no such effect (Figure 3B; Figure S3, Supporting Information). Interactions between MED27 and MED28, MED29, or MED30 were not disrupted by any of the patient‐specific mutations in our Co‐IP experiments (Figure S3, Supporting Information).

To further identify proteins with disrupted interactions with mutant MED27, we conducted affinity purification coupled with mass spectrometry (AP‐MS) (Figure 3C). A total of 41 proteins were found to differentially interact with the p.P280L mutant MED27 compared to WT MED27. Among these, 21 proteins interacted exclusively with the mutant MED27, 19 proteins interacted exclusively with the WT MED27, and one protein interacted with both but exhibited variable interaction strength (Table S2, Supporting Information; see the Experimental Section for experimental and data analysis details). Consistent with in silico modeling and Co‐IP results, 12 MED subunits, including MED20 and MED22, were identified in the WT MED27 interactome. In contrast, only one MED subunit was detected in the mutant MED27 interactome (Figure 3D). This significant reduction in MED subunit interactions further supports the hypothesis that patient‐specific MED27 mutation destabilize the MED complex. DisGeNET analysis of the differentially interacting proteins between WT and mutant MED27 revealed enrichment of phenotypes highly relevant to the clinical presentations of *MED27* patients, including cerebral atrophy, hyperreflexia, developmental regression, mental deficiency, and dystonia (Figure 3E). These findings validate the reliability of our AP‐MS results and support our hypothesis that P280L mutant MED27 disrupts the conformation of the MED complex, leading to less stable interactions among its subunits.

### 2.4 Germline LoF of *Med27* Induced Peri‐implantation Lethality in Mice

**Figure 4:**
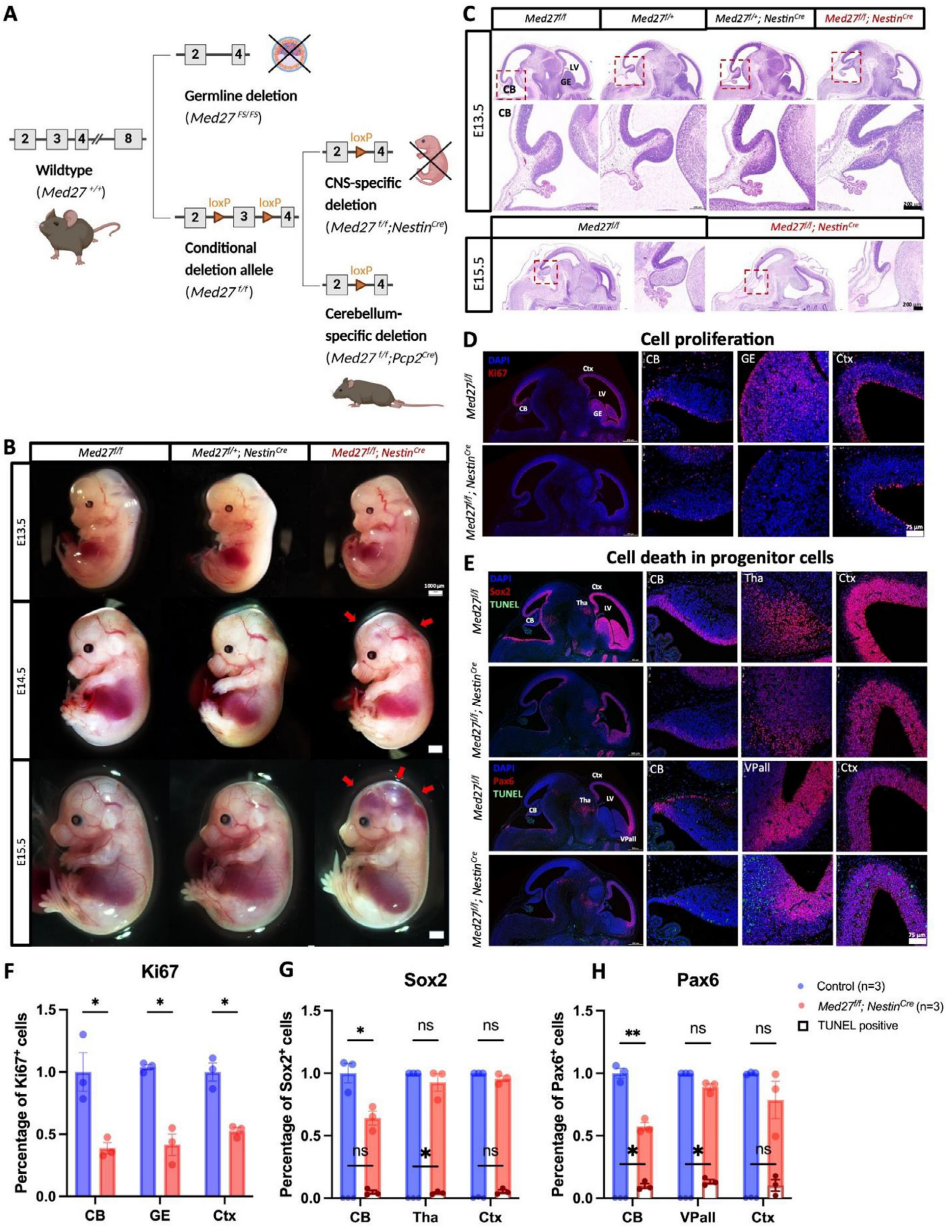
Perinatal lethality and disruption of CNS development in *Med27* CNS LoF embryos. A) Illustration of the three *Med27* mouse models generated by us, including global LoF, CNS‐specific LoF, and cerebellum‐specific LoF of the gene. Created with BioRender.com. B) Representative images of *Med27^f/f^*, *Med27^f/+^; Nestin^Cre^* and *Med27^f/f^; Nestin^Cre^* embryos at E13.5, E14.5 and E15.5. The *Med27^f/f^; Nestin^Cre^* embryos showed hemorrhaging starting from E14.5 (red arrows), scale bar = 1000 µm. C) Representative H&E staining results of *Med27^f/f^, Med27^f/+^, Med27^f/+^; Nestin^Cre^* and *Med27^f/f^; Nestin^Cre^* embryo brains, scale bar = 200 µm. D) Immunofluorescence (IF) staining images of the proliferation marker Ki67 on E13.5 embryo brains, scale bar = 75 µm. E) Representative IF images of progenitor markers Sox2 and Pax6 with TUNEL staining on E13.5 embryo brains, scale bar = 75 µm. Quantification analysis of F) Ki67^+^, G) Sox2^+^, and H) Pax6^+^ cell percentages in each brain regions at E13.5. Multiple unpaired *t*‐tests were used for analysis in (F)–(H); *, **, and ns denote *p* < 0.05, *p* < 0.01,— and *p* >0.05, respectively. CB‐cerebellar, Ctx‐cortex, GE‐ganglionic eminence, LV‐lateral ventricle, Tha‐thalamus, VPall‐ventral pallium.

Inspired by the LoF FS and splice site variants observed in patients, as well as the potential LoF effects of the missense variant demonstrated by the experimental data above, we first generated a germline deletion of exon 3 of mouse *Med27* through CRISPR/Cas9. This introduced an FS mutation, resulting in a global knockout (KO) of the gene (**Figure** **4**A; Figure S4A, Supporting Information, *Med27^FS^* ^/^ *^FS^*). Heterozygote mating between *Med27^FS^* ^/+^ mice allowed us to pinpoint the stage of lethality for homozygous KO embryos to between embryonic days 3.5 (E3.5) and E6.5, indicating a failure of implantation (Figure S4B, Supporting Information). This lethality timing mirrors previously reported observations in *Med20* and *Med28* KO mice.^[^ ^34^, ^35^ ^]^ In addition, abnormal morphology was noted in *Med27^FS^* ^/^ *^FS^* embryos at the E3.5 blastocyst stage (Figure S4C, Supporting Information).

### 2.5 CNS‐Specific LoF of *Med27* Caused Perinatal Lethality with Cerebellar Agenesis in Mice

The early embryonic death of global *Med27* KO mice prompted us to generate mice with conditional LoF of *Med27*. Using CRISPR/Cas9‐induced homologous recombination, we inserted *LoxP* sites into introns 2 and 3 of *Med27* (Figure 4A; Figure S5A, Supporting Information, *Med27^f/f^*). As *MED27* patients predominantly exhibit neurological defects and brain structural anomalies, we generated CNS‐specific conditional KO (CKO) mice by breeding *Med27^f/f^* mice with Nestin‐Cre mice (Figure 4A; Figure S5A, Supporting Information, *Med27^f/f^*;*Nestin^Cre^*). Heterozygote mating revealed that CNS CKO mice died around birth (Figure S5B, Supporting Information).

To determine the cause of lethality in *Med27^f/f^*;*Nestin^Cre^* mice, we analyzed their morphological and histological features. Hemorrhaging was observed starting at E14.5 and became more pronounced by E15.5 (Figure 4B). MRI scans revealed an expansion of the lateral ventricles and extensive hemorrhaging localized to the ganglionic eminence and hindbrain regions at E14.5. These features were exacerbated by E15.5 (Figure S5C, Supporting Information). Histological analysis confirmed severe brain deformities, including cerebellar agenesis, by E15.5 (Figure 4C). Further investigation revealed that these anomalies were associated with abnormal cell proliferation, particularly in progenitor cells. IF staining for Ki67 demonstrated decreased cell proliferation in the cerebellar, ganglionic eminence, and cortical regions (Figure 4D,F). In addition, increased apoptosis of progenitor cells was observed, as indicated by co‐staining of Sox2 or Pax6 with positive TUNEL signals (Figure 4E,G,H). Interestingly, while the amount of Pax6‐expressing cells, a key TF for cerebellar neuron migration,^[^ ^36^, ^37^ ^]^ remained unchanged in other regions, it was significantly reduced in the cerebellum (Figure 4E,H), underscoring the critical role for Med27 in cerebellar development. Collectively, these results illustrated the essential functions of Med27 during early CNS development, particularly in cerebellar morphogenesis and progenitor cell proliferation.

### 2.6 Cerebellum‐Specific LoF of *Med27* Led to Progressive Cerebellar Atrophy and Ataxia in Mice

Inspired by the predominant cerebellar abnormalities observed in *Med27* CNS CKO embryos and in *MED27* patients, we generated cerebellum‐specific *Med27* CKO mice to investigate its role in cerebellar development. Purkinje cells (PCs), which are GABAergic inhibitory neurons, serve as the sole output neurons of the cerebellar network and are critical for cerebellar function.^[^ ^38^, ^39^, ^40^ ^]^ To create a PC‐specific LoF of *Med27*, we bred *Med27^f^* ^/^ *^f^* mice with Purkinje cell protein‐2 (Pcp2)‐Cre mice (Figure 4A, *Med27^f/f^*;*Pcp2^Cre^*). Pcp2‐Cre mice are widely used in studies of cerebellar development.^[^ ^41^, ^42^ ^]^

**Figure 5:**
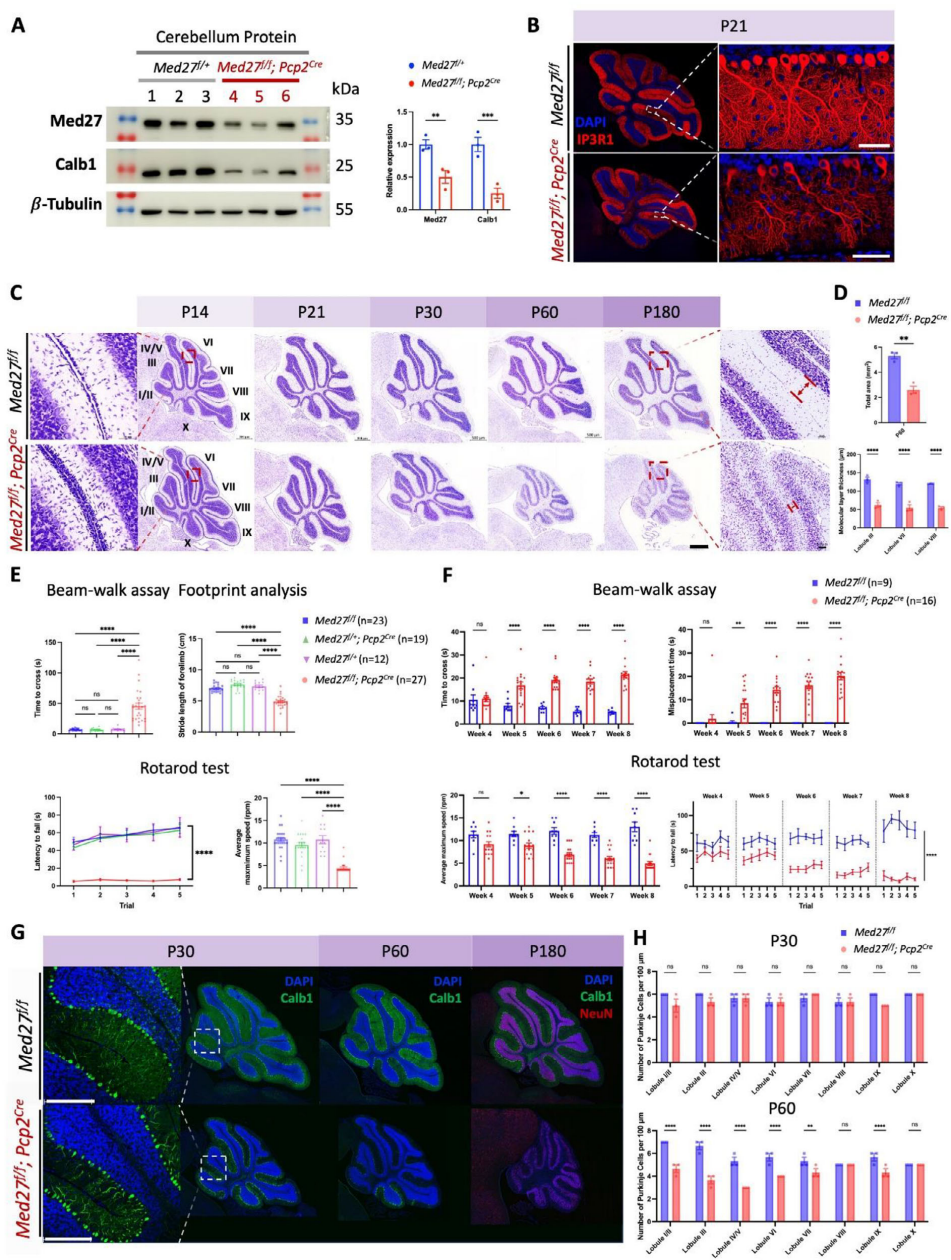
Progressive cerebellar atrophy and ataxia in *Med27* cerebellum LoF mice. A) Representative Western blot images and quantification of the decreased Med27 and Calbindin (Calb1) protein levels in P30 *Med27^f/f^*;*Pcp2^Cre^* cerebellum (sample size *n* = 3). B) Representative IF staining images illustrating IP3R1 expression in PCs from P21 mice, scale bar = 50 µm. C) Representative Nissl staining images of the cerebellums at different ages, scale bar = 500 µm (whole cerebellum images) or 50 µm (enlarged views). D) Quantitative comparisons of the total cerebellar area and molecular layer thicknesses in lobule III, VII, and VIII based on Nissl staining results at P60 (*n* = 3). E) Beam‐walk assay, footprint analysis, and rotarod test results in P60 *Med27^f/f^*, *Med27^f/+^*, *Med27^f/+^*;*Pcp2^Cre^*, and *Med27^f/f^*;*Pcp2^Cre^* mice. F) Longitudinal tracking of motor behavioral deficits in *Med27* cerebellum CKO mice by beam‐walk and rotarod tests. G) Representative Calb1 and NeuN IF staining images in *Med27* cerebellum CKO mice at different ages, scale bar = 200 µm. H) Quantification of PCs density in all cerebellar lobules based on IF staining results of Calb1 on P30 and P60 mice (*n* = 3). For statistical analysis, two‐tailed Student's *t*‐test was used in (A), (D), and (E), one‐way ANOVA was used in (F), and two‐way ANOVA was used in (H); *, **, ***, ****, and ns denote *p* < 0.05, *p* < 0.01, *p* < 0.001, *p* < 0.0001, and *p* > 0.05, respectively.

To evaluate the KO efficiency of *Med27* in the cerebellum of *Med27^f/f^*;*Pcp2^Cre^* mice, we compared protein levels in the cerebellum of mutant mice to WT littermates. A significant reduction in Med27 protein levels was observed, though mRNA levels remained unchanged (**Figure** **5**A; Figure S6A, Supporting Information). In addition, expression of several genes essential for cerebellar development, including Calbindin (Calb1, a representative PC marker), Pcp2, and Skor2, were markedly reduced in mutant mice (Figure 5A; Figure S6A,B, Supporting Information).

The first three postnatal weeks in mice are critical for PC development.^[^ ^43^, ^44^ ^]^ By P21, *Med27^f/f^*;*Pcp2^Cre^* PCs exhibited immature morphologies, including reduced dendritic arborization and smaller, irregular somas, as visualized by IF staining for PC markers IP3R1, PKCα, and Calb1 (Figure 5B; Figure S7A, Supporting Information). These findings suggest impaired dendritogenesis and delayed PC development in mutant mice.

Mature PCs typically receive excitatory inputs from a single climbing fiber (CF) originating in the inferior olive and multiple parallel fibers (PFs) from granule cells (GCs).^[^ ^45^, ^46^ ^]^ Staining of presynaptic markers for PF terminals (VGlut1) and CF terminals (VGlut2) revealed impaired synapses formation in *Med27^f/f^*;*Pcp2^Cre^* mice (Figure S7B, Supporting Information). Specifically, excessive PF and CF presynaptic terminals were observed, along with unsuccessful translocation of CF terminals. This was evident as VGlut2‐positive puncta were enriched around PC soma rather than at their dendrites (Figure S7B, Supporting Information). These findings indicate that the loss of Med27 resulted in abnormal dendritic growth and impaired synapses formation during the early stages of PC maturation.

Beyond PC abnormalities, a noticeable reduction in cerebellum size was observed in *Med27^f/f^*;*Pcp2^Cre^* mice. This reduction progressed over time, from P14 to P180 (Figure 5C,D; Figure S8A–D, Supporting Information), accompanied by thinning of the molecular layer (Figure 5D; Figure S8A–D, Supporting Information), indicative of dendritic loss in PCs. These structural deficits contributed to motor impairments in mutant mice. Behavioral assays revealed significant motor coordination and balance deficits compared to littermate controls, including longer traverse times during the beam‐walk assay, irregular gait patterns with shorter stride lengths and wider step widths via footprint analysis, and decreased latency to fall in the rotarod test (Figure 5E; Figure S9A–D, Supporting Information). Mutant mice also exhibited ataxia‐like behavior, characterized by arched back and lower body weights (Figure S9E and Movie S1, Supporting Information). In contrast, littermate controls (*Med27^f/f^, Med27^f/+^*, and *Med27^f/+^;Pcp2^Cre^*) displayed normal behavior with no significant differences among the three genotypes.

Longitudinally observations revealed that *Med27^f/f^*;*Pcp2^Cre^* mice initially exhibited comparable motor capacities and PC counts to controls at four weeks of age (Figure 5F; Figures S8E and S9A,D, Supporting Information), although the cerebellum size and PC dendritic arborization were already reduced at this stage (Figure 5G; Figure S8, Supporting Information). By five weeks of age, mutant mice began to show early signs of motor deficits. At two months, severely ataxia and prominent cerebellar hypoplasia became evident (Figure 5F; Figure S9A–D, Supporting Information). By P180, PCs were nearly absent, and there was a significant reduction in GCs, despite normal survival rates (Figure 5G; Figure S9E, Supporting Information). Thus, cerebellum‐specific loss of *Med27* in mice resulted in progressive motor dysfunction and cerebellar atrophy, resembling the phenotypes observed in *MED27* patients. These findings provide strong evidence that Med27 is essential for normal cerebellum development, PC maintenance, and the integrity of the cerebellar network.

### 2.7 Dysregulation of the Transcriptional Landscape due to LoF of *Med27* in the Cerebellum

**Figure 6:**
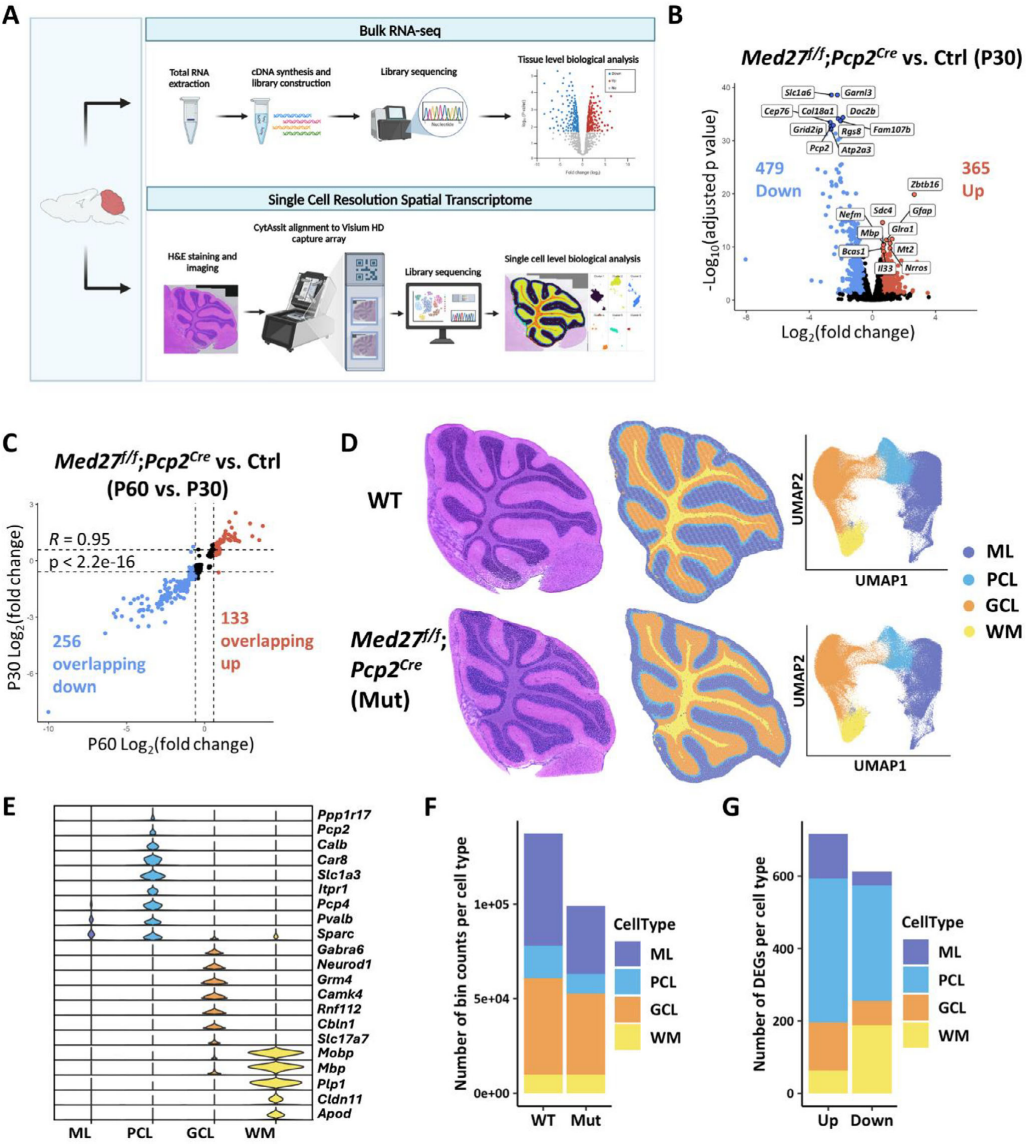
Dysregulation of the transcriptional landscape due to LoF of *Med27* in the cerebellum. A) Schematic diagram of bulk RNA‐seq and sc‐ST experimental design. B) Volcano plot showing bulk RNA‐seq result comparing P30 *Med27^f/f^*;*Pcp2^Cre^* and their littermate control mice's cerebellum transcriptomic profiles. 479 downregulated and 365 upregulated DEGs were identified (|Log_2_foldchange| > 0.58, adjusted *p* < 0.05). Gene symbols of the top 10 down‐ and upregulated DEGs were labeled. C) Correlation plot comparing DEGs identified in P30 and P60 mice by Pearson's correlation test. Dots represent the common and concurrent DEGs identified in both groups. D) H&E staining images (left), spatial clustering of sc‐ST generated Visium spots color coded by cell type identity (middle), and UMAP visualization of the Visium spots (right) for WT and *Med27^f/f^*;*Pcp2^Cre^* (Mut) mice cerebellums. ML, molecular layer; GCL, granule cell layer; PCL, Purkinje cell layer; WM, white matter. E) Violin plots illustrating expression levels of different marker genes used for cell clustering. F) Absolute counts per cell type in WT and Mut sample, respectively. G) DEG counts for up‐ and downregulated genes within each cell type.

Given the essential role of MED in transcription initiation, we hypothesized that the abnormalities in *Med27^f/f^*;*Pcp2^Cre^* mice were caused by transcriptional dysregulation of functionally critical genes. Longitudinal studies of these mice established that P30 marks the initial onset of cerebellar atrophy and motor deficits, while P60 represents a stage of disease progression (Figure 5; Figures S8 and S9, Supporting Information). To investigate the underlying molecular mechanisms, we performed transcriptomic profiling of cerebellar tissues from both time points using bulk RNA‐seq (**Figure** **6**A). At P30, we identified 479 downregulated and 365 upregulated genes, including significant reductions in genes specifically or highly expressed in PCs, such as *Slc1a6*, *Pcp2*, *Atp2a3*, and *Rgs8* (Figure 6B). GO analysis of downregulated DEGs revealed enrichment for biological processes related Pol II‐mediated transcription regulation, neuronal development and differentiation, and axon guidance—all consistent with the transcriptional regulatory role of Med27 and the observed phenotypic abnormalities in mutant mice (Figure S10A, Supporting Information). By P60, the expression of PC‐ and cerebellum‐related genes, such as *Trpc3, Slc1a6*, *Pcp2*, and *Inpp5a*, was further reduced (Figure S10B,C, Supporting Information). A comparison between the two time points revealed a high correlation in overlapping DEGs (*R* = 0.95 by Pearson's correlation), with a greater proportion of the overlapping DEGs being downregulated (Figure 6C; Figure S10D, Supporting Information).

Since bulk RNA‐seq analysis reflected an aggregate of gene expression profiles from the entire cerebellum, it may mask cell‐type‐specific transcriptional dysregulation. To address this limitation, we performed single‐cell‐resolution spatial transcriptomics (sc‐ST) to uncover additional DEGs and examine gene expression changes in their original positional context (Figure 6A). sc‐ST was conducted on H&E‐stained cerebellar sections from P30 mice, and data were processed using the 10X Genomics Visium HD platform and the “Space Ranger” pipeline, resulting in 8 µm binned spots. We captured 137796 bins (average 495.1 UMI/bin) in the WT cerebellum and 99331 bins (average 588.8 UMI/bin) in the mutant cerebellum. After quality control, data integration using Harmony for batch correction was visualized via UMAP projection (Figure S11A, Supporting Information). Unsupervised clustering analysis of the integrated data identified 17 clusters (Figure S11B, Supporting Information), which were further annotated based on cell‐type‐specific marker genes (Figure S11C, Supporting Information).^[^ ^47^, ^48^ ^]^ Four major cerebellar cell types—molecular layer (ML), Purkinje cell layer (PCL), granule cell layer (GCL), and white matter (WM)—were defined based on marker gene expression and cerebellar structure (Figure 6D,E; Figure S11D, Supporting Information). To validate the accuracy of our cell clustering, we applied the Robust Cell Type Decomposition (RCTD) method^[^ ^49^ ^]^ with a cerebellar single‐cell reference data^[^ ^48^ ^]^ to deconvolute the Visium spots. The RCTD UMAP distributions for major cell types were highly consistent with those identified via unsupervised clustering (Figure S12, Supporting Information), confirming the reliability of our cell‐type annotations.

Since the WT cerebellum had a larger area compared to the mutant (Figure 5C; Figure S8D, Supporting Information), the total bin count was higher in the WT sample (Figure 6F). In terms of cell‐type proportions, LoF of *Med27* resulted in losses of PCL, ML, and GCL cells compared to WT, consistent with histological staining results (Figure S11E, Supporting Information; Figure 5G,H). To identify cell‐type‐specific dysregulated genes, we performed DEG analysis within each cell type. The majority of DEGs were identified in the PCL, with 176 downregulated DEGs overlapping with those from bulk RNA‐seq results (Figure 6G).

### 2.8 Downregulation of the Cerebellum Development Master Regulator *Lhx1* due to LoF of *Med27*

**Figure 7:**
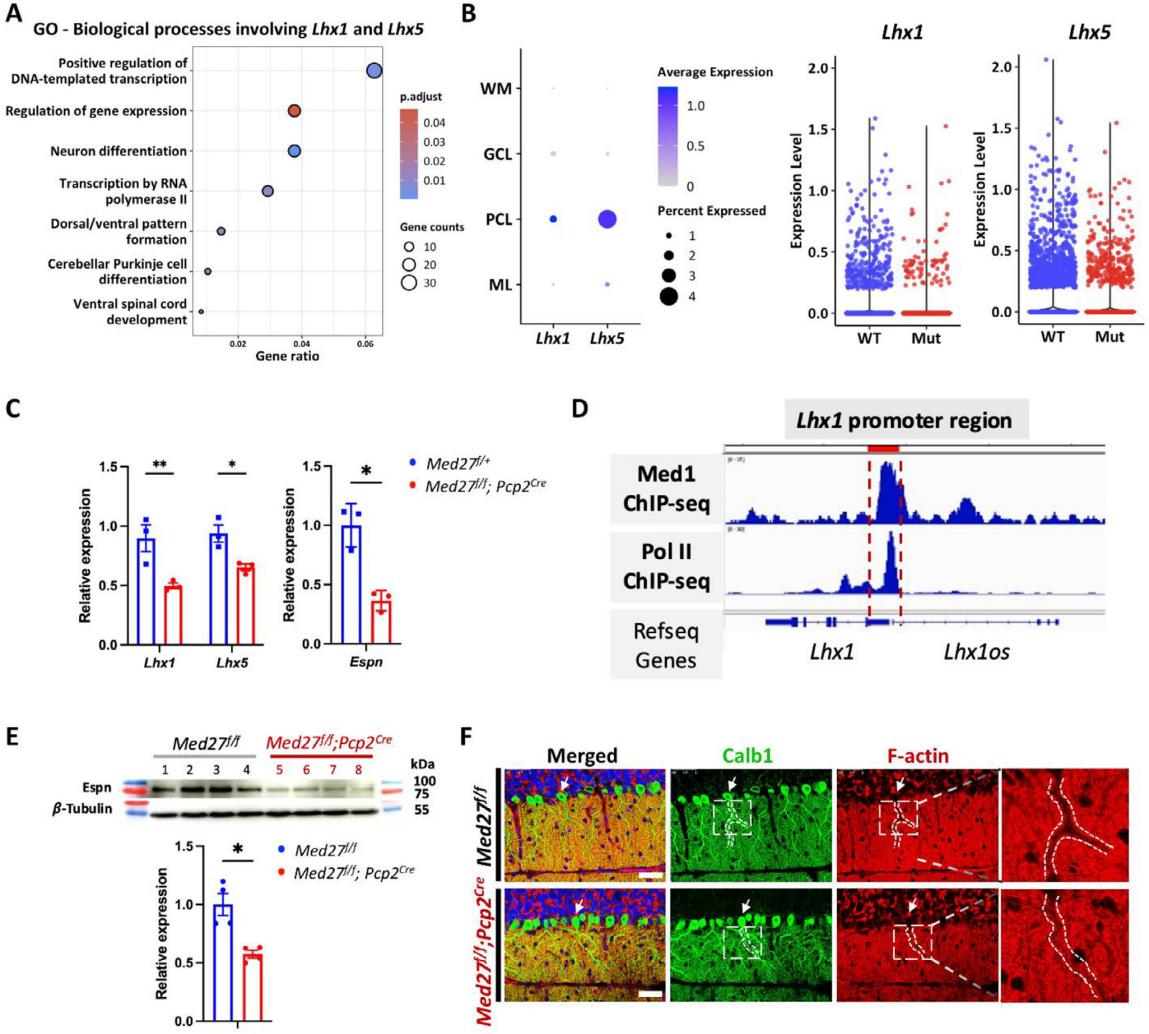
Downregulation of *Lhx1* due to LoF of *Med27* in the cerebellum. A) Biological processes including the *Lhx1* and *Lhx5* genes through GO analysis of downregulated DEGs identified by bulk RNA‐seq in P30 mice. B) Dot plot showing *Lhx1* and *Lhx5* expression levels in all four cell types (left) and in PCL cells (right) identified through sc‐ST analysis. C) qPCR results of *Lhx1*, *Lhx5*, and *Espn* genes at P30 (*n* = 3). D) Genomic occupancy profiles of Med1 and Pol II at the *Lhx1* gene locus (*Lhx1* promoter region: chr1184337678‐84339554, NCBI37/mm9 genomic assembly). ChIP‐seq results were obtained from GSE109043, GSM2928425, GSE36027, GSM918725 datasets. E) Representative Western blot image and quantification of the Espn expression levels at P21 (*n* = 4). β‐Tubulin was used as an endogenous control. F) Representative IF staining images showing the expression of Calb1 in PC and mis‐localized F‐actin in the PC dendrites at P21, scale bars = 50 µm. Two‐way ANOVA and two‐tailed Student's *t*‐tests were used for statistical analysis in (C) and (E). * and ** denote *p* < 0.05 and *p* < 0.01, respectively.

TFs play pivotal roles in regulating gene expression. To identify functionally critical genes directly regulated by Med27, we specifically examined commonly dysregulated TFs identified through sc‐ST and bulk RNA‐seq. Among these, *Lhx1*, which encodes the master regulator TF LIM homeobox protein 1, stood out (**Figure** **7**A) due to its well‐established roles in embryonic brain development and cerebellar PC differentiation,^[^ ^50^ ^]^ as previously illustrated by us.^[^ ^51^, ^52^ ^]^ Expression of *Lhx1*, as well as its closely related TF *Lhx5*, was significantly decreased in cerebellums with *Med27* LoF, as demonstrated by bulk RNA‐seq (data not shown), sc‐ST analysis (Figure 7B), and RT‐qPCR (Figure 7C).

Our previous studies have shown that cerebellar LoF of *Lhx1* resulted in abnormal den dritogenesis and spine morphogenesis in PCs,^[^ ^52^ ^]^ findings that are consistent with the current observations in cerebellums with *Med27* LoF (Figure 5B; Figure S7, Supporting Information). Analysis of publicly available ChIP‐seq data for Med1 and Pol II in mice^[^ ^19^, ^53^ ^]^ revealed that MED directly occupies the promoter region of *Lhx1*, suggesting that MED regulates *Lhx1* expression (Figure 7D). Since the functional integrity of the MED complex relies on all its components, LoF of *Med27* likely destabilizes the MED complex, thereby disrupting the MED‐*Lhx1* promoter interaction and impairing *Lhx1* expression in the cerebellum.

Our earlier work also demonstrated that cerebellar LoF of *Lhx1* led to decreased expression of Espin, an actin‐regulatory protein specifically expressed in PC dendrites.^[^ ^54^ ^]^ This disruption resulted in improper F‐actin localization in PC dendrites and spines.^[^ ^52^ ^]^ Consistently, in cerebellums with *Med27* LoF, Espin expression was significantly reduced (Figure 7C,E), accompanied by mislocalized F‐actin in PC dendrites (Figure 7F). These findings further support the interpretation that Med27 functions through Lhx1 to regulate cerebellum development and PCs differentiation.

## 3 Discussion

Understanding the pathogenicity mechanisms underlying NDDs is crucial for effective diagnosis and treatment. Our previous report identified a novel NDD‐causing gene, *MED27*, associated with an autosomal recessive monogenic disorder in patients who uniformly exhibited cerebellar atrophy (or hypoplasia) with movement disorders.^[^ ^9^ ^]^ Currently, functional characterization of MED27 remains limited. Previous studies have shown that chemically induced mutagenesis of *med27* in zebrafish caused reductions in the retinal amacrine cell layer,^[^ ^55^ ^]^ while KO of the gene led to lethality at the pupal stage in flies^[^ ^56^, ^57^ ^]^ and partial embryonic lethality in chickens.^[^ ^58^ ^]^ In addition, KO of *Med27* resulted in cell death in mouse B cells, T cells, and mouse ESCs.^[^ ^59^ ^]^ These findings are consistent with our own observation of early embryonic lethality in mice with complete LoF of *Med27* (Figure S4, Supporting Information), illustrating the indispensable role of MED27 during embryonic development.

We analyzed published bulk RNA‐seq data to compare *MED27*/*Med27* expression levels across diverse human and mouse tissue types (Figure S13, Supporting Information). Bulk RNA‐seq data indicate that the gene is abundantly and ubiquitously expressed across various tissue types, with no particularly elevated levels in brain tissues. Thus, the neural‐specific phenotypes observed in *MED27* patients cannot be solely explained by its expression levels. We further analyzed single‐nuclei RNA‐sequencing (snRNA‐seq) data in the human and mouse cerebellum, given the prominent cerebellar atrophy phenotype seen in *MED27* patients. In both organisms, its expression is high in PCs, which are the primary output neurons in the cerebellum, potentially highlighting its critical role in cerebellar function.

Given the lack of knowledge regarding its disease mechanism and the unknown functions of MED27 during neuronal development, our study aimed to address these gaps using the in vitro and in vivo preclinical models we generated. Our in vitro model—hESCs and NPCs carrying the most recurrent *MED27* variant identified in our patient cohort—were designed to replicate the dysregulations occurring during early embryogenesis and early neuronal differentiation in patients. Meanwhile, our in vivo models, consisting of mice with constitutive or tissue‐specific LoF of *Med27*, aimed to mimic the potential partial LoF mechanism observed in *MED27* patients. Since MED, together with Pol II, is essential for initiating transcription of most protein‐coding and non‐coding genes, we hypothesize that mutant MED27 dysregulates different downstream target genes during key developmental processes. Correspondingly, our in vitro models captured its downstream targets during early neurogenesis, while our in vivo models apprehended these targets during cerebellar development.

In our in vitro studies, we first identified dysregulated genes involved in biological processes such as nervous system development and neuron differentiation in *MED27* mutant hESCs (Figure 1). These findings were consistent with the defective ectodermal differentiation capacity observed in mutant hESCs, while their ability to differentiate into endodermal and mesodermal lineages remained intact (Figure S1, Supporting Information). This observation also aligns with the predominant neuronal lineage defects seen in *MED27* patients. Further investigation of hESC‐derived NPCs revealed molecular changes caused by mutant MED27 during early neurogenesis. Specifically, we observed malfunctioning transcriptional machinery, including disrupted MED and Pol II occupancy, as well as altered chromatin interactions in mutant NPCs. In addition, we demonstrated decreased stability of MED, characterized by reduced interactions between mutant MED27 and its neighboring MED subunits (Figure 3). This instability likely contributes to altered MED occupancy and chromatin interactions, suggesting a potential LoF conformational change in the MED. These abnormalities collectively explained the disturbed transcriptomic profile observed in mutant NPCs.

We investigated the expression of all MED subunit genes across our bulk RNA‐seq datasets, which include data from hESCs, human NPCs, and mouse cerebellar tissues from cerebellum‐specific knockout mice at P30 and P60. In addition, we incorporated published RNA‐seq results from cardiomyocyte‐specific *Med27* knockout mouse models at embryonic day 11.5 and four weeks of age.^[^ ^60^ ^]^ As summarized in Figure S14A (Supporting Information), compared between mutant and control samples, very few MED subunit genes showed significant dysregulation (defined by |Log_2_foldchange| ≥ 0.58 and adjusted *p* < 0.05). These findings indicate that, at the transcriptional level, mutant MED27 in human cells and LoF of Med27 in mouse tissues generally do not affect the expression of other MED subunits.

In the previous study,^[^ ^60^ ^]^ although *Med27* knockout in mouse cardiomyocytes did not affect the transcriptional levels of other Mediator subunit genes, all tested Mediator core subunits showed reduced protein expression as detected by Western blot analysis. The authors proposed that Med27 plays an essential role in maintaining the integrity and stability of the Mediator complex. This aligns with our proposed pathogenic mechanism, in which patient‐specific *MED27* mutations result in LoF and lead to Mediator complex instability. Consistently, in our Western blot analysis, we also observed significantly reduced levels of MED1/Med1 (Mediator middle submodule) and MED23/Med23 (Mediator tail submodule) in mutant human cells and mouse tissues (Figure S14B,C, Supporting Information).

Currently, all reported patients with germline *MED* gene variants exhibit NDD phenotypes (Table S4, Supporting Information). Common features include growth failure, motor/speech delay, cognitive impairment, intellectual disability, hypotonia, and brain structural abnormalities. Despite these shared features, the exact symptoms and their severities vary across patients, even among those with variants in the same gene. Similar to *MED27* variants, patient‐specific variants in other MED genes destabilize the MED complex. For instance, a c.1013‐5A>G splicing variant in *MED17* caused a frameshift and truncated protein, leading to drastically reduced interactions with MED12, MED15, MED4, and MED22 compared to WT MED17 in Co‐IP experiments.^[^ ^61^ ^]^ Similarly, the p.(Tyr39Cys) missense variant in *MED25* impaired interactions with MED1, MED6, MED14, MED16, MED23, MED24, and CDK8.^[^ ^62^ ^]^ Variants in kinase module genes, such as *MED12* and *MED13L*, also caused impaired recruitment or reduced interaction with other kinase module subunits.^[^ ^63^, ^64^ ^]^ These findings corroborate our proposed pathogenic mechanism for patient‐specific *MED27* variants, which implicates MED complex stability as a key factor.

We also identified *EGR1* and *FOS*—two IEGs—as top candidates for direct downstream targets of MED27 during early neurogenesis. Both gene are well‐established master regulatory TFs in neuronal development and plasticity, playing critical roles in both embryonic and adult brain function.^[^ ^20^, ^24^, ^65^ ^]^ IEGs enable rapid and dynamic responses to intrinsic and extrinsic stimuli, followed by a second wave of transcriptional regulation that encodes enduring adaptions and affects widespread neuronal functions. Previous research has shown that these genes were transcriptionally regulated by MED subunits. For example, in *Med23* KO mouse ESCs, *Egr1* mRNA was the most significantly downregulated gene identified in microarray analyses. MED occupancy was assayed by ChIP‐qPCR using antibodies for Med1 and Med17 in a single IP reaction. Results showed that, upon serum stimulation, MED occupancy to the *Egr1* promoter region was reduced approximately threefold in *Med23* ^−/−^ cells compared to WT cells. Similar ChIP‐qPCR experiments using Pol II antibody also showed an approximately threefold decrease in Pol II occupancy at the *Egr1* promoter in *Med23* ^−/−^ cells relative to WT cells.^[^ ^25^ ^]^ This reduction in *Egr1* persisted regardless of serum induction.^[^ ^26^ ^]^ Similarly, in Hela cells with siRNA‐mediated knockdown of *MED14* and in a human neuroblastoma cell line with CRISPR/Cas9‐mediated knockdown of *MED12*, serum‐induced *EGR1* and *FOS* mRNA levels were both downregulated. In HCT116 cells with lentiviral‐mediated *MED14* knockdown, both *FOS* and *EGR1* mRNA levels were reduced by ≈50%, corroborating an ≈50% reduction in MED14 and Pol II occupancy at their promoters, as detected by ChIP‐qPCR.^[^ ^27^, ^66^ ^]^ These findings suggest that LoF changes in MED subunits result in the downregulation of these IEGs through decreased MED and Pol II occupancies at their promoters, consistent with our proposed pathogenic mechanism for *MED27* mutations. This further supports a potential LoF mechanism for the *MED27* mutation analyzed in our study.

Interestingly, in the first report linking *MED23* to an NDD syndrome, *FOS* was the most upregulated gene in patient‐derived skin fibroblasts, while *EGR1* expression remained unchanged.^[^ ^28^ ^]^ Furthermore, in immortalized lymphoblastoid cells from seven *MED12*‐related NDD patients—who carried different disease‐causing *MED12* variants and exhibited variable phenotypes—distinct expression patterns of *EGR1* and *FOS* were observed upon serum induction, with some showing upregulation and others downregulation.^[^ ^27^ ^]^ Nevertheless, the degree of MED and Pol II promoter occupancies versus mRNA levels of these genes was consistently correlated. Specifically, ChIP‐qPCR experiments conducted at the promoters of these IEGs showed that the recruitment of Pol II and MED12 at their respective promoter paralleled the expression levels of *JUN*, *FOS* and *EGR1*. For example, lower Pol II and MED12 occupancies at the promoter correlated with lower mRNA levels of the gene. This promoter occupancy/mRNA level pattern is specific for each MED12 mutation. Similarly, in our study, we observed that the recruitment of Pol II and MED1 at their respective promoters paralleled the expression levels of *FOS* and *EGR1*, suggesting a similar molecular phenotype for MED12 and MED27 mutations.

The authors of these studies proposed that the phenotypic diversity in these patients may result from impaired fine‐tuning of IEG expression during development, and that altered IEG expression could serve as a molecular signature for cognitive deficits. Our findings echoed these previous reports, demonstrating that IEGs are first‐tier essential TFs influenced by mutant MED27 during early neurogenesis. Notably, the simultaneous downregulation of *EGR1* and *FOS* in our study further supports a potential LoF effect of the recurrent missense *MED27* variant we analyzed.

We further investigated in vivo mouse models with LoF of *Med27* and demonstrated its indispensable roles during early embryogenesis and CNS development. Constitutive LoF of *Med27* led to embryonic lethality and implantation failure as early as E3.5 (Figure S4, Supporting Information). CNS‐specific LoF of *Med27* resulted in severe brain structural defects, with the most prominent feature being cerebellar agenesis (Figure 4), consistent with the homogenous cerebellar abnormalities observed in *MED27* patients.^[^ ^9^, ^11^ ^]^ Given that these mice were not viable after birth, we generated cerebellum‐specific LoF *Med27* mice, which successfully recapitulated the progressive motor deficits and cerebellar atrophy phenotypes seen in *MED27* patients (Figure 5; Figures S6–S9, Supporting Information). Through comprehensive transcriptomic profiling (Figure 6), we identified *Lhx1* as a potential direct target of Med27 during cerebellar development (Figure 7). Since our earlier report,^[^ ^51^ ^]^ Lhx1 has been well‐established as a master regulatory TF in embryonic brain development and cerebellar PC differentiation.^[^ ^50^, ^52^ ^]^ Comparing our current cerebellum‐specific LoF *Med27* mouse model with our previous cerebellum‐specific LoF *Lhx1* mouse model, we observed striking similarities between the two. Both models exhibited identical abnormalities in PC dendritogenesis and spine morphogenesis and affected the same downstream pathways (Figure S7, Supporting Information; Figure 7). These findings provide strong evidence that Med27 functions through Lhx1 in the cerebellum.

To obtain a more comprehensive understanding of the functional deficits caused by mutant MED27, we compared bulk RNA‐seq data obtained from different models. We first examined whether the same DEGs could be identified across all four sample types: hESCs, NPCs, and *Med27*‐cKO mouse cerebellar tissues at P30 and P60. As shown in Figure S15 (Supporting Information), there is limited overlap between human and mouse samples, with only three DEGs showing consistent changes (upregulated or downregulated in all four models) (Figure S15A, Supporting Information). Abundant concordant DEGs were observed between P30 and P60 mouse tissues, as expected, while the comparison between human hESCs and NPCs demonstrated much less concordance (Figure S15A, Supporting Information). Supporting this observation, clustering analysis using all overlapping homologous DEGs from human and mouse models revealed three distinct clusters: hESCs, NPCs, and P30/P60 mouse tissues (Figure S15B, Supporting Information).

Given the significant divergence due to species differences, we next performed separate analyses for human and mouse samples. Clustering analysis using all DEGs from hESCs and NPCs categorized them into two groups consistent with their cell type identity. Meanwhile, when clustering was performed using intersected DEGs (i.e., concordant overlapping DEGs between hESCs and NPCs), the samples were grouped into control cells versus *MED27* mutant cells (Figure S15C, Supporting Information). For mouse tissues, the high similarity between P30 and P60 samples resulted in consistent clustering into two categories—control mice or cKO mice—whether all DEGs or intersected DEGs were used (Figure S15D, Supporting Information).

Despite the differences in DEG sets across sample types, we observed enrichment of similar biological processes through GO analysis. Specifically, when comparing the top 15 biological process pathways identified for each sample type, seven were consistently enriched across all four sample types (Figure S15E, Supporting Information). Notably, a large proportion of the overlapping pathways were associated with Pol II‐regulated transcription (Figure S15F, Supporting Information). These findings collectively suggest that while distinct DEG sets were identified across different sample types, they converge on similar pathways related to transcriptional regulation, consistent with our proposed functional defects of mutant MED27.

Notably, while our in vitro results suggest a partial LoF disease mechanism, the mouse models utilized in this study carried genotypes (KO of *Med27*) that differed from those observed in *MED27* patients (compound heterozygous of a LoF and a missense variant, or a homozygous missense variant). Future experiments involving mice with patient‐specific *Med27* missense mutations would be valuable to confirm whether they indeed result in a LoF effect. Furthermore, different missense variants may exhibit distinct pathogenicity mechanisms. For instance, the p.P259L variant was predicted to increase the stability of MED and did not demonstrate reduced interactions with neighboring MED subunits, unlike other *MED27* variants (Figure 3). Additionally, our behavioral assays primarily focused on evaluating motor functions and balance in mutant mice. Unfortunately, due to their severe motor deficits, it was challenging to assess their cognitive functions and social behaviors accurately. As a result, the cognitive deficiencies observed in *MED27* patients could not be fully evaluated in our current models.

Due to the unavailability of MED27 antibodies for ChIP or CUT&Tag, this study utilized MED1 CUT&Tag seq to represent the DNA occupancy profile of the MED complex. Future experiments involving the addition of a small tag to the endogenous MED27, followed by CUT&Tag seq using an antibody against this tag, could facilitate the mapping of the endogenous genomic distribution of MED27. However, such experiments must be approached with caution, as adding a tag may disrupt the conformation of the MED complex.

Although CUT&Tag seq is a powerful tool for detecting protein–DNA interactions at high resolution, it may not fully capture all forms of MED complex destabilization. For instance, even when the MED complex is destabilized, enhancer and promoter regions may remain in close proximity due to the presence of strong CTCF loop anchors and cohesin proteins that maintain chromatin compartmentalization. Furthermore, MED complex destabilization may not result in complete disassembly. Disrupted MED subunits may still remain DNA‐occupying activity, as reported in previous studies.^[^ ^5^, ^67^ ^]^ Consequently, CUT&Tag may detect residue MED subunits bound to DNA, underestimating the extent of destabilization. In addition, transient or dynamic protein‐DNA interactions are challenging to detect using CUT&Tag, as it captures only a static snapshot. Future studies integrating CUT&Tag seq with complementary high‐resolution experimental approaches will provide a more comprehensive understanding of MED complex stabilization and its downstream effects, such as using Capture‐C or Micro‐Capture‐C to reveal enhancer‐promoter interactions and 3D chromatin architecture alterations at nucleosome resolution in regions occupied by the MED complex,^[^ ^68^, ^69^ ^]^ or single‐molecule imaging to provide insights into the dynamic changes in protein interactions that are beyond the capacity of CUT&Tag.^[^ ^70^, ^71^ ^]^

Although the dysregulated transcriptional mechanisms resulting from mutant MED27 are addressed to some extent, the current study has limitations in mechanistic resolution. For instance, the Hi‐C experiment performed in the current study provided a genome‐side view of chromatin organization with moderate resolution (10 kb). Future studies using advanced approaches, such as Micro‐C, could generate high‐resolution chromatin architecture maps (≈200 bp) and capture fine‐scale interactions.^[^ ^72^ ^]^ In addition, examining the binding of cohesin proteins and CTCF at chromatin loops could clarify whether mutant MED27 and MED destabilization affect chromatin interactions through architectural proteins. Furthermore, different TFs recruit MED to activate downstream genes, partly through their binding at distinct enhancer regions.^[^ ^3^ ^]^ MED27 mutation may disrupt TF‐MED interactions. Future experiments, such as Co‐IP coupled with MS, could delineate changes in the TF‐MED interactome and provide insights into enhancer‐promoter looping mechanisms. Finally, while the destabilization of MED was established in this study using semi‐quantitative Co‐IP and TAP‐MS assays, future approaches, such as cryo‐electron microscopy, could enable direct visualization of structural alterations in the MED complex caused by mutant MED27. Such studies would provide a more detailed understanding of the molecular mechanisms underlying *MED27*‐related phenotypes.

## 4 Conclusion 

Taken together, our pathogenicity study supports a partial LoF mechanism underlying this *MED27*‐associated NDD (Figure S16, Supporting Information). Patient‐specific mutant MED27 induces instability in MED, leading to altered DNA occupancy and chromatin interaction, which subsequently dysregulate the transcription of critical downstream genes, including master regulatory TFs. Given the ubiquitous expression and essential roles of MED27, its mutation likely impacts distinct downstream targets at various developmental stages, such as the IEGs during early neurogenesis and *Lhx1*/*Lhx5* during cerebellar development, as identified in our study. This study established MED27 as essential for maintaining MED complex integrity and orchestrating neurodevelopment, while also providing a framework for understanding “neuro‐MEDopathies” caused by pathogenic variants in other MED subunits and guiding the development of targeted treatments.

## 5 Experimental Section

Please refer to the Supporting Information file for methodologies and materials used in this study.

## Conflict of Interest

The authors declare no conflict of interest.

## Author Contributions

N.Y., X.L., and T.G. contributed equally to this work. S.G. conceived the study, designed the experiments, and wrote the manuscript. N.Y. and T.G. conducted the mouse studies, while X.L., H.Z., and L.Y. performed the cellular studies. T.G. and L.G. carried out bioinformatics analysis. Y.F., Y.Q., and H.W. performed the Hi‐C experiments and data analysis. Y.L.L. and K.M.K. provided assistance with Lhx1‐related analyses. N.C. and Y.L. conducted proteomics predictions. H.K. assisted with mouse brain MRI scans. P.L., H.H.C., L.M., and X.C. provided guidance on the project. All authors critically reviewed the manuscript and approved its content.

## Ethical Statement

All animal experiments were approved by the Animal Experimentation Ethics Committee (AEEC) at CUHK and all the experimental procedures were conducted in the accordance of AEEC guidelines at CUHK.

## Supporting information



Supporting Information

Supplemental Movie 1

Supplemental Table 1

Supplemental Table 2

Supplemental Table 3

Supplemental Table 4

## Data Availability

The data that support the findings of this study are available from the corresponding author upon reasonable request.

## References

[1] X. Chen , X. Yin , J. Li , Z. Wu , Y. Qi , X. Wang , W. Liu , Y. Xu , Science 2021, 372, eabg0635. 33958484 10.1126/science.abg0635

[2] S. Rengachari , S. Schilbach , S. Aibara , C. Dienemann , P. Cramer , Nature 2021, 594, 129. 33902108 10.1038/s41586-021-03555-7

[3] W. F. Richter , S. Nayak , J. Iwasa , D. J. Taatjes , Nat. Rev. Mol. Cell Biol. 2022, 23, 732. 35725906 10.1038/s41580-022-00498-3 PMC9207880

[4] M. G. Jaeger , B. Schwalb , S. D. Mackowiak , T. Velychko , A. Hanzl , H. Imrichova , M. Brand , B. Agerer , S. Chorn , B. Nabet , F. M. Ferguson , A. C. Muller , A. Bergthaler , N. S. Gray , J. E. Bradner , C. Bock , D. Hnisz , P. Cramer , G. E. Winter , Nat. Genet. 2020, 52, 719. 32483291 10.1038/s41588-020-0635-0 PMC7610447

[5] L. El Khattabi , H. Zhao , J. Kalchschmidt , N. Young , S. Jung , P. Van Blerkom , P. Kieffer‐Kwon , K. R. Kieffer‐Kwon , S. Park , X. Wang , J. Krebs , S. Tripathi , N. Sakabe , D. R. Sobreira , S. C. Huang , S. S. P. Rao , N. Pruett , D. Chauss , E. Sadler , A. Lopez , M. A. Nobrega , E. L. Aiden , F. J. Asturias , R. Casellas , Cell 2019, 178, 1145e20. 31402173 10.1016/j.cell.2019.07.011 PMC7533040

[6] M. H. Kagey , J. J. Newman , S. Bilodeau , Y. Zhan , D. A. Orlando , N. L. van Berkum , C. C. Ebmeier , J. Goossens , P. B. Rahl , S. S. Levine , D. J. Taatjes , J. Dekker , R. A. Young , Nature 2010, 467, 430. 20720539 10.1038/nature09380 PMC2953795

[7] C. Wang , Y. Xing , J. Zhang , M. He , J. Dong , S. Chen , H. Wu , H. Y. Huang , C. H. Chou , L. Bai , F. He , J. She , A. Su , Y. Wang , P. A. Thistlethwaite , H. D. Huang , J. X. Yuan , Z. Y. Yuan , J. Y. Shyy , Circ. Res. 2022, 131, 828. 36252121 10.1161/CIRCRESAHA.122.321532

[8] W. S. Tang , L. Weng , X. Wang , C. Q. Liu , G. S. Hu , S. T. Yin , Y. Tao , N. N. Hong , H. Guo , W. Liu , H. R. Wang , T. J. Zhao , Cell Rep. 2021, 36, 109314. 34233190 10.1016/j.celrep.2021.109314

[9] L. Meng , P. Isohanni , Y. Shao , B. H. Graham , S. E. Hickey , S. Brooks , A. Suomalainen , P. Joset , K. Steindl , A. Rauch , A. Hackenberg , F. A. High , A. Armstrong‐Javors , N. E. Mencacci , P. Gonzalez‐Latapi , W. A. Kamel , J. Y. Al‐Hashel , B. I. Bustos , A. V. Hernandez , D. Krainc , S. J. Lubbe , H. Van Esch , C. De Luca , K. Ballon , C. Ravelli , L. Burglen , L. Qebibo , D. G. Calame , T. Mitani , D. Marafi , et al., Ann. Neurol. 2021, 89, 828. 33443317 10.1002/ana.26019

[10] E. Cali , S. J. Lin , C. Rocca , Y. Sahin , A. Al Shamsi , S. El Chehadeh , M. Chaabouni , K. Mankad , E. Galanaki , S. Efthymiou , S. Sudhakar , A. Athanasiou‐Fragkouli , T. Celik , N. Narli , S. Bianca , D. Murphy , F. M. De Carvalho Moreira , S. Y. S. Group , A. Andrea , C. Petree , K. Huang , K. Monastiri , M. Edizadeh , R. Nardello , M. Ognibene , P. De Marco , M. Ruggieri , F. Zara , P. Striano , Y. Sahin , et al., Genet. Med. 2022, 24, 2194. 36001086 10.1016/j.gim.2022.07.013 PMC10519206

[11] R. Maroofian , R. Kaiyrzhanov , E. Cali , M. Zamani , M. S. Zaki , M. Ferla , D. Tortora , S. Sadeghian , S. M. Saadi , U. Abdullah , E. G. Karimiani , S. Efthymiou , G. Yesil , S. Alavi , A. M. Al Shamsi , H. Tajsharghi , M. S. Abdel‐Hamid , N. W. Saadi , F. Al Mutairi , L. Alabdi , C. Beetz , Z. Ali , M. B. Toosi , S. Rudnik‐Schoneborn , M. Babaei , P. Isohanni , J. Muhammad , S. Khan , M. Al Shalan , S. E. Hickey , et al., Brain 2023, 146, 5031. 37517035 10.1093/brain/awad257 PMC10690011

[12] L. W. Koblan , J. L. Doman , C. Wilson , J. M. Levy , T. Tay , G. A. Newby , J. P. Maianti , A. Raguram , D. R. Liu , Nat. Biotechnol. 2018, 36, 843. 29813047 10.1038/nbt.4172 PMC6126947

[13] F. A. Ran , P. D. Hsu , J. Wright , V. Agarwala , D. A. Scott , F. Zhang , Nat. Protoc. 2013, 8, 2281. 24157548 10.1038/nprot.2013.143 PMC3969860

[14] B. Hu , R. Yang , Z. Cheng , S. Liang , S. Liang , N. Yin , F. Faiola , J. Hazard. Mater. 2020, 393, 122440. 32151936 10.1016/j.jhazmat.2020.122440

[15] M. Alowaysi , M. Al‐Shehri , M. Baadhaim , H. AlZahrani , D. Aboalola , M. Daghestani , H. Hashem , R. Aljahdali , R. Salem , A. Alharbi , M. Muharraq , K. Alghamdi , F. Alsobiy , A. Zia , R. Lehmann , J. Tegner , K. Alsayegh , Stem Cell Res. 2023, 71, 103158. 37406498 10.1016/j.scr.2023.103158

[16] Y. Shan , Z. Liang , Q. Xing , T. Zhang , B. Wang , S. Tian , W. Huang , Y. Zhang , J. Yao , Y. Zhu , K. Huang , Y. Liu , X. Wang , Q. Chen , J. Zhang , B. Shang , S. Li , X. Shi , B. Liao , C. Zhang , K. Lai , X. Zhong , X. Shu , J. Wang , H. Yao , J. Chen , D. Pei , G. Pan , Nat. Commun. 2017, 8, 672. 28939884 10.1038/s41467-017-00668-4 PMC5610324

[17] V. Martinez‐Cerdeno , S. C. Noctor , Front. Neuroanat. 2018, 12, 104. 30574073 10.3389/fnana.2018.00104 PMC6291443

[18] D. Hnisz , B. J. Abraham , T. I. Lee , A. Lau , V. Saint‐Andre , A. A. Sigova , H. A. Hoke , R. A. Young , Cell 2013, 155, 934. 24119843 10.1016/j.cell.2013.09.053 PMC3841062

[19] M. Quevedo , L. Meert , M. R. Dekker , D. H. W. Dekkers , J. H. Brandsma , D. L. C. van den Berg , Z. Ozgur , I. W. F. J. van , J. Demmers , M. Fornerod , R. A. Poot , Nat. Commun. 2019, 10, 2669. 31209209 10.1038/s41467-019-10502-8 PMC6573065

[20] F. Duclot , M. Kabbaj , Front. Behav. Neurosci. 2017, 11, 35. 28321184 10.3389/fnbeh.2017.00035 PMC5337695

[21] L. Lu , X. Liu , W. K. Huang , P. Giusti‐Rodriguez , J. Cui , S. Zhang , W. Xu , Z. Wen , S. Ma , J. D. Rosen , Z. Xu , C. F. Bartels , R. Kawaguchi , M. Hu , P. C. Scacheri , Z. Rong , Y. Li , P. F. Sullivan , H. Song , G. L. Ming , Y. Li , F. Jin , Mol. Cell 2020, 79, 521. 32592681 10.1016/j.molcel.2020.06.007 PMC7415676

[22] W. Y. Choi , J. H. Hwang , A. N. Cho , A. J. Lee , I. Jung , S. W. Cho , L. K. Kim , Y. J. Kim , Mol. Cells 2020, 43, 1011. 33293480 10.14348/molcells.2020.0207 PMC7772509

[23] F. Inoue , A. Kreimer , T. Ashuach , N. Ahituv , N. Yosef , Cell Stem Cell 2019, 25, 713. 31631012 10.1016/j.stem.2019.09.010 PMC6850896

[24] P. P. Tregub , Y. K. Komleva , M. V. Kukla , A. S. Averchuk , A. S. Vetchinova , N. A. Rozanova , S. N. Illarioshkin , A. B. Salmina , Cells 2025, 14, 143. 39851571 10.3390/cells14020143 PMC11763428

[25] G. Wang , M. A. Balamotis , J. L. Stevens , Y. Yamaguchi , H. Handa , A. J. Berk , Mol. Cell 2005, 17, 683. 15749018 10.1016/j.molcel.2005.02.010

[26] W. Wang , X. Yao , Y. Huang , X. Hu , R. Liu , D. Hou , R. Chen , G. Wang , Transcription 2013, 4, 39. 23340209 10.4161/trns.22874 PMC3644042

[27] L. M. Donnio , B. Bidon , S. Hashimoto , M. May , A. Epanchintsev , C. Ryan , W. Allen , A. Hackett , J. Gecz , C. Skinner , R. E. Stevenson , A. P. M. de Brouwer , C. Coutton , C. Francannet , P. S. Jouk , C. E. Schwartz , J. M. Egly , Hum. Mol. Genet. 2017, 26, 2062. 28369444 10.1093/hmg/ddx099

[28] S. Hashimoto , S. Boissel , M. Zarhrate , M. Rio , A. Munnich , J. M. Egly , L. Colleaux , Science 2011, 333, 1161. 21868677 10.1126/science.1206638

[29] B. Bonev , G. Cavalli , Nat. Rev. Genet. 2016, 17, 661. 27739532 10.1038/nrg.2016.112

[30] M. J. Rowley , A. Poulet , M. H. Nichols , B. J. Bixler , A. L. Sanborn , E. A. Brouhard , K. Hermetz , H. Linsenbaum , G. Csankovszki , E. Lieberman Aiden , V. G. Corces , Genome Res. 2020, 30, 447. 32127418 10.1101/gr.257832.119 PMC7111518

[31] S. Zhang , N. Ubelmesser , N. Josipovic , G. Forte , J. A. Slotman , M. Chiang , H. J. Gothe , E. G. Gusmao , C. Becker , J. Altmuller , A. B. Houtsmuller , V. Roukos , K. S. Wendt , D. Marenduzzo , A. Papantonis , Sci. Adv. 2021, 7, abg8205. 10.1126/sciadv.abg8205 PMC8535795 34678064

[32] S. Zhang , N. Ubelmesser , M. Barbieri , A. Papantonis , Nat. Genet. 2023, 55, 832. 37012454 10.1038/s41588-023-01364-4

[33] A. R. Ardakany , H. T. Gezer , S. Lonardi , F. Ay , Genome Biol. 2020, 21, 256. 32998764 10.1186/s13059-020-02167-0 PMC7528378

[34] W. Cui , C. Marcho , Y. Wang , R. Degani , M. Golan , K. D. Tremblay , J. A. Rivera‐Perez , J. Mager , Reproduction 2019, 157, 215. 30571656 10.1530/REP-18-0508 PMC6545164

[35] L. Li , R. M. Walsh , V. Wagh , M. F. James , R. L. Beauchamp , Y. S. Chang , J. F. Gusella , K. Hochedlinger , V. Ramesh , PLoS One 2015, 10, 0140192. 10.1371/journal.pone.0140192 PMC4596692 26445504

[36] J. Yeung , T. J. Ha , D. J. Swanson , D. Goldowitz , J. Neurosci. 2016, 36, 9057. 27581449 10.1523/JNEUROSCI.4385-15.2016 PMC5005719

[37] D. Engelkamp , P. Rashbass , A. Seawright , V. van Heyningen , Development 1999, 126, 3585. 10409504 10.1242/dev.126.16.3585

[38] A. L. Joyner , N. S. Bayin , Development 2022, 149, dev185587. 36172987 10.1242/dev.185587 PMC9641654

[39] K. Leto , M. Arancillo , E. B. Becker , A. Buffo , C. Chiang , B. Ding , W. B. Dobyns , I. Dusart , P. Haldipur , M. E. Hatten , M. Hoshino , A. L. Joyner , M. Kano , D. L. Kilpatrick , N. Koibuchi , S. Marino , S. Martinez , K. J. Millen , T. O. Millner , T. Miyata , E. Parmigiani , K. Schilling , G. Sekerkova , R. V. Sillitoe , C. Sotelo , N. Uesaka , A. Wefers , R. J. Wingate , R. Hawkes , Cerebellum 2016, 15, 789. 26439486 10.1007/s12311-015-0724-2 PMC4846577

[40] M. Sepp , K. Leiss , F. Murat , K. Okonechnikov , P. Joshi , E. Leushkin , L. Spanig , N. Mbengue , C. Schneider , J. Schmidt , N. Trost , M. Schauer , P. Khaitovich , S. Lisgo , M. Palkovits , P. Giere , L. M. Kutscher , S. Anders , M. Cardoso‐Moreira , I. Sarropoulos , S. M. Pfister , H. Kaessmann , Nature 2024, 625, 788. 38029793 10.1038/s41586-023-06884-x PMC10808058

[41] J. J. Barski , K. Dethleffsen , M. Meyer , Genesis 2000, 28, 93. 11105049

[42] A. Slugocka , J. Wiaderkiewicz , J. J. Barski , Cerebellum 2017, 16, 191. 26969183 10.1007/s12311-016-0770-4 PMC5243870

[43] G. C. Beekhof , C. Osorio , J. J. White , S. van Zoomeren , H. van der Stok , B. Xiong , I. H. Nettersheim , W. A. Mak , M. Runge , F. R. Fiocchi , H. J. Boele , F. E. Hoebeek , M. Schonewille , Elife 2021, 10, 63668. 10.7554/eLife.63668 PMC8195607 33973524

[44] I. Dusart , F. Flamant , Front. Neuroanat. 2012, 6, 11. 22514522 10.3389/fnana.2012.00011 PMC3324107

[45] M. Ito , M. Kano , Neurosci. Lett. 1982, 33, 253. 6298664 10.1016/0304-3940(82)90380-9

[46] M. Coesmans , J. T. Weber , C. I. De Zeeuw , C. Hansel , Neuron 2004, 44, 691. 15541316 10.1016/j.neuron.2004.10.031

[47] L. Tejwani , N. G. Ravindra , C. Lee , Y. Cheng , B. Nguyen , K. Luttik , L. Ni , S. Zhang , L. M. Morrison , J. Gionco , Y. Xiang , J. Yoon , H. Ro , F. Haidery , R. M. Grijalva , E. Bae , K. Kim , R. T. Martuscello , H. T. Orr , H. Y. Zoghbi , H. S. McLoughlin , L. P. W. Ranum , V. G. Shakkottai , P. L. Faust , S. Wang , D. van Dijk , J. Lim , Neuron 2024, 112, 362. 38016472 10.1016/j.neuron.2023.10.039 PMC10922326

[48] V. Kozareva , C. Martin , T. Osorno , S. Rudolph , C. Guo , C. Vanderburg , N. Nadaf , A. Regev , W. G. Regehr , E. Macosko , Nature 2021, 598, 214. 34616064 10.1038/s41586-021-03220-z PMC8494635

[49] D. M. Cable , E. Murray , L. S. Zou , A. Goeva , E. Z. Macosko , F. Chen , R. A. Irizarry , Nat. Biotechnol. 2022, 40, 517. 33603203 10.1038/s41587-021-00830-w PMC8606190

[50] R. McMahon , T. Sibbritt , N. Salehin , P. Osteil , P. P. L. Tam , Dev. Growth Differ. 2019, 61, 327. 31111476 10.1111/dgd.12609

[51] Y. Zhao , K. M. Kwan , C. M. Mailloux , W. K. Lee , A. Grinberg , W. Wurst , R. R. Behringer , H. Westphal , Proc. Natl. Acad. Sci. USA 2007, 104, 13182. 17664423 10.1073/pnas.0705464104 PMC1941824

[52] N. C. Lui , W. Y. Tam , C. Gao , J. D. Huang , C. C. Wang , L. Jiang , W. H. Yung , K. M. Kwan , Nat. Commun. 2017, 8, 15079. 28516904 10.1038/ncomms15079 PMC5454373

[53] F. Yue , Y. Cheng , A. Breschi , J. Vierstra , W. Wu , T. Ryba , R. Sandstrom , Z. Ma , C. Davis , B. D. Pope , Y. Shen , D. D. Pervouchine , S. Djebali , R. E. Thurman , R. Kaul , E. Rynes , A. Kirilusha , G. K. Marinov , B. A. Williams , D. Trout , H. Amrhein , K. Fisher‐Aylor , I. Antoshechkin , G. DeSalvo , L. H. See , M. Fastuca , J. Drenkow , C. Zaleski , A. Dobin , P. Prieto , et al., Nature 2014, 515, 355.25409824

[54] G. Sekerkova , P. A. Loomis , B. Changyaleket , L. Zheng , R. Eytan , B. Chen , E. Mugnaini , J. R. Bartles , J. Neurosci. 2003, 23, 1310. 12598619 10.1523/JNEUROSCI.23-04-01310.2003 PMC2854510

[55] K. Durr , J. Holzschuh , A. Filippi , A. K. Ettl , S. Ryu , I. T. Shepherd , W. Driever , Genetics 2006, 174, 693. 16582438 10.1534/genetics.105.055152 PMC1602071

[56] J. Gokcezade , G. Sienski , P. Duchek , G3 2014, 4, 2279. 25236734 10.1534/g3.114.014126 PMC4232553

[57] D. Li‐Kroeger , O. Kanca , P. T. Lee , S. Cowan , M. T. Lee , M. Jaiswal , J. L. Salazar , Y. He , Z. Zuo , H. J. Bellen , Elife 2018, 7, 38709. 10.7554/eLife.38709 PMC6095692 30091705

[58] K. Tsujino , Y. Okuzaki , N. Hibino , K. Kawamura , S. Saito , Y. Ozaki , S. Ishishita , A. Kuroiwa , S. Iijima , Y. Matsuda , K. Nishijima , T. Suzuki , Dev. Growth Differ. 2019, 61, 393. 31613003 10.1111/dgd.12631

[59] W. A. Whyte , D. A. Orlando , D. Hnisz , B. J. Abraham , C. Y. Lin , M. H. Kagey , P. B. Rahl , T. I. Lee , R. A. Young , Cell 2013, 153, 307. 23582322 10.1016/j.cell.2013.03.035 PMC3653129

[60] S. Zhu , Z. Chen , C. Liu , J. Duong , T. Tran , Z. Liang , X. Fang , K. Ouyang , Life Sci. 2024, 356, 123020. 39209248 10.1016/j.lfs.2024.123020

[61] T. Terabayashi , S. Hashimoto , Neurogenetics 2021, 22, 353. 34392449 10.1007/s10048-021-00661-6

[62] L. Basel‐Vanagaite , P. Smirin‐Yosef , J. L. Essakow , S. Tzur , I. Lagovsky , I. Maya , M. Pasmanik‐Chor , A. Yeheskel , O. Konen , N. Orenstein , M. Weisz Hubshman , V. Drasinover , N. Magal , G. Peretz Amit , Y. Zalzstein , A. Zeharia , M. Shohat , R. Straussberg , D. Monte , M. Salmon‐Divon , D. M. Behar , Hum. Genet. 2015, 134, 577. 25792360 10.1007/s00439-015-1541-x

[63] H. Zhou , J. M. Spaeth , N. H. Kim , X. Xu , M. J. Friez , C. E. Schwartz , T. G. Boyer , Proc. Natl. Acad. Sci. USA 2012, 109, 19763. 23091001 10.1073/pnas.1121120109 PMC3511715

[64] T. Smol , F. Frenois , M. Billotte , R. Caumes , L. A. Menke , A. Nassar‐Sheikh Rashid , C. Thuillier , D. Monte , F. Petit , A. Verger , J. Ghoumid , HGG Adv. 2025, 6, 100467. 40500968 10.1016/j.xhgg.2025.100467 PMC12221883

[65] T. Wells , K. Rough , D. A. Carter , Front. Mol. Neurosci. 2011, 4, 6. 21629823 10.3389/fnmol.2011.00006 PMC3099308

[66] M. D. Galbraith , J. Saxton , L. Li , S. J. Shelton , H. Zhang , J. M. Espinosa , P. E. Shaw , Nucleic Acids Res. 2013, 41, 10241. 24049075 10.1093/nar/gkt837 PMC3905876

[67] F. Sun , T. Sun , M. Kronenberg , X. Tan , C. Huang , M. F. Carey , Genes Dev. 2021, 35, 1175. 34301767 10.1101/gad.348471.121 PMC8336890

[68] D. J. Downes , A. L. Smith , M. A. Karpinska , T. Velychko , K. Rue‐Albrecht , D. Sims , T. A. Milne , J. O. J. Davies , A. M. Oudelaar , J. R. Hughes , Nat. Protoc. 2022, 17, 445. 35121852 10.1038/s41596-021-00651-w PMC7613269

[69] J. C. Hamley , H. Li , N. Denny , D. Downes , J. O. J. Davies , Nat. Protoc. 2023, 18, 1687. 36991220 10.1038/s41596-023-00817-8

[70] W. K. Cho , J. H. Spille , M. Hecht , C. Lee , C. Li , V. Grube , I. I. Cisse , Science 2018, 361, 412. 29930094 10.1126/science.aar4199 PMC6543815

[71] Y. E. Guo , J. C. Manteiga , J. E. Henninger , B. R. Sabari , A. Dall'Agnese , N. M. Hannett , J. H. Spille , L. K. Afeyan , A. V. Zamudio , K. Shrinivas , B. J. Abraham , A. Boija , T. M. Decker , J. K. Rimel , C. B. Fant , T. I. Lee , I. I. Cisse , P. A. Sharp , D. J. Taatjes , R. A. Young , Nature 2019, 572, 543. 31391587 10.1038/s41586-019-1464-0 PMC6706314

[72] I. Jerkovic , G. Cavalli , Nat. Rev. Mol. Cell Biol. 2021, 22, 511. 33953379 10.1038/s41580-021-00362-w

