# Supplementary material for: Pathogenicity of Mediator Complex Subunit 27 (MED27) in a Neurodevelopmental Disorder with Cerebellar Atrophy
Source: Adv Sci (Weinh). 2025 Sep 29;12(48):e05535. doi: 10.1002/advs.202505535 (PMC12752580; doi:10.1002/advs.202505535)
Supplement: Supplementary file 1 — Supporting Information [file ADVS-12-e05535-s003.pdf]

Supporting Information

Pathogenicity of Mediator Complex Subunit 27 (*MED27*) in a Neurodevelopmental Disorder with Cerebellar Atrophy

*Nuermila Yiliyaer, Xiaocheng Li, Tianyu Guo, Haiying Zhou, Lihai Gong, Luowei Yuan, Yang Fu, Yulong Qiao, Ying Lam Lui, Nuo Chen, Pengfei Lin, Hoi Hung Cheung, Ho Ko, Linyan Meng, Xiao Chen, Yong Lei, Kin Ming Kwan, Huating Wang, Shen Gu**

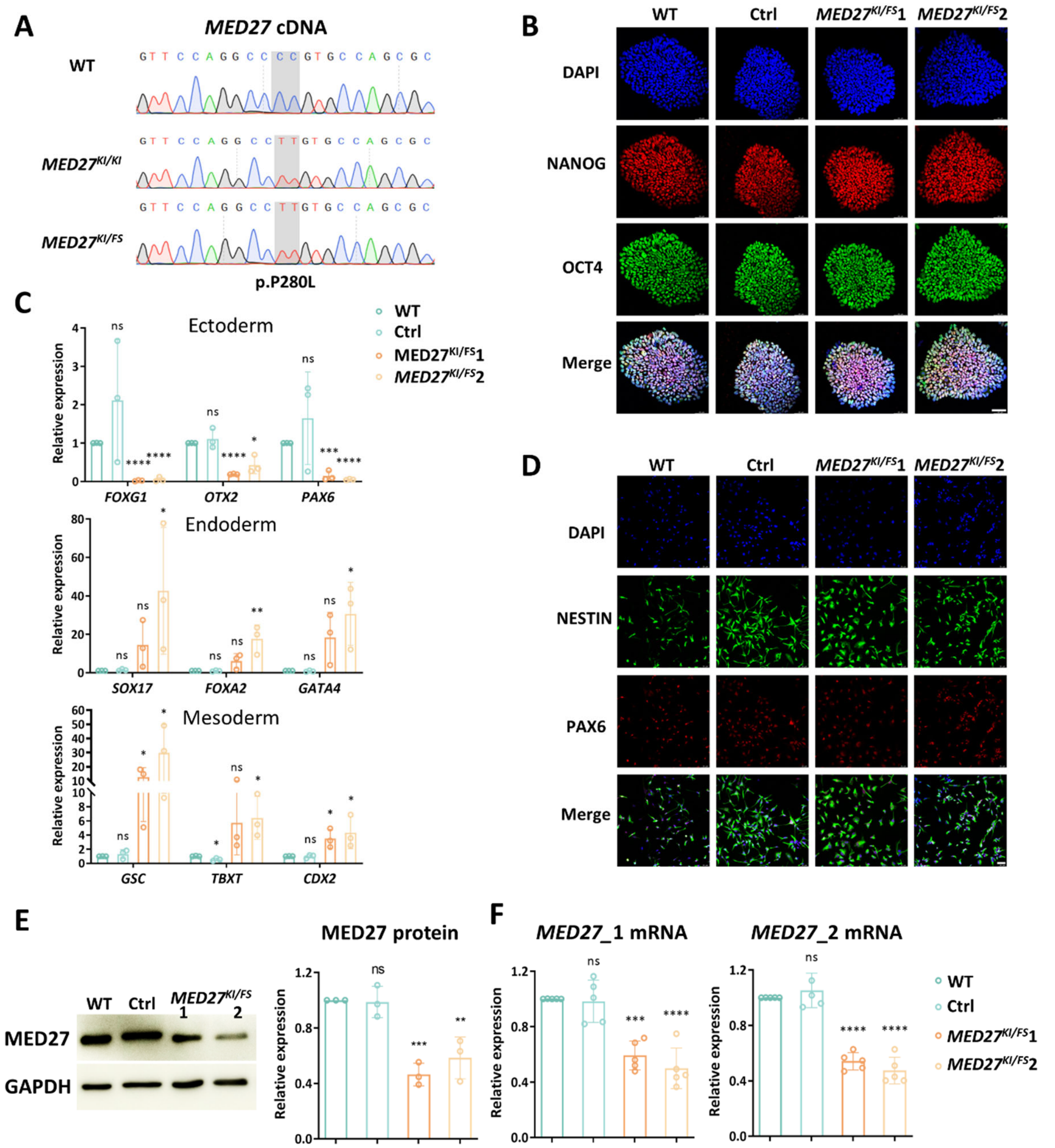

Figure S1 Characterization of human stem cells with patient-specific *MED27* variants. (A) Representative Sanger sequencing results of *MED27* cDNA in wildtype (WT), *MED27*$^{KI/KI}$, and *MED27*$^{KI/FS}$ cells. (B) Representative IF staining images of pluripotency markers in hESCs. Scale bar = 50 µm. (C) qPCR of marker genes representing the three germ layers examined after hESC spontaneous differentiation. (D) Representative IF staining images of marker genes in differentiated NPCs. Scale bar = 50 µm. (E) Representative western blot image and quantification of MED27 protein expression levels in *MED27*$^{KI/KI}$ and *MED27*$^{KI/FS}$ NPCs compared to the WT and control NPCs. GAPDH was used as an endogenous control. (F) qPCR results illustrating *MED27* mRNA levels in *MED27*$^{KI/FS}$ NPCs compared to the WT and control NPCs. In Figure S1C, 1E and 1F, data were generated from at least three biological replicates. Error bars represent SD of the mean. One-way ANOVA was used for statistical analysis. *, **, ***, ****, and ns denote $p<0.05$, $p<0.01$, $p<0.001$, $p<0.0001$, and $p>0.05$, respectively.

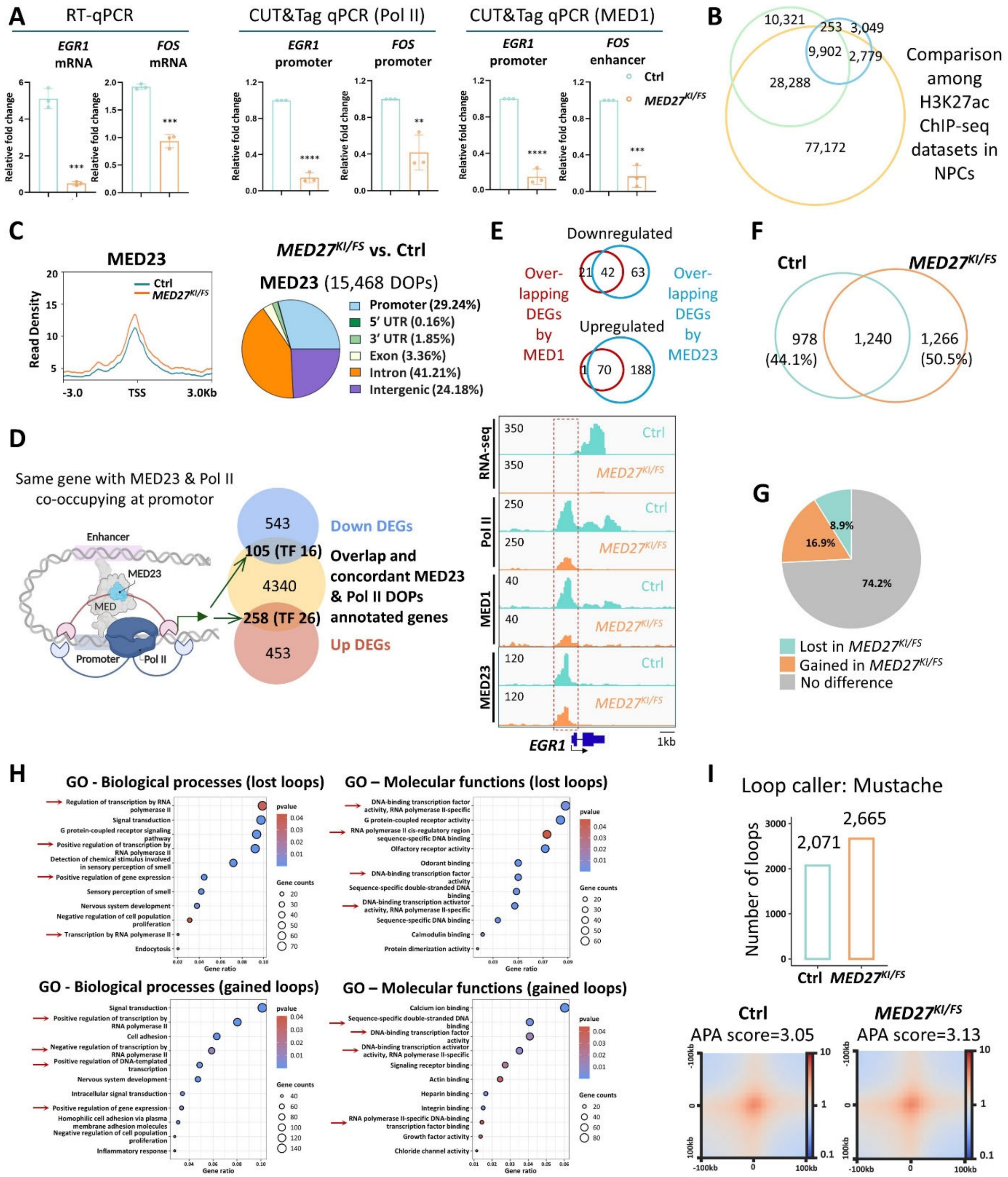

Figure S2 Active enhancer regions defined in NPCs and Hi-C loop counts. (A) RT-qPCR and CUT&Tag qPCR validation for *EGR1* and *FOS* genes' mRNA levels and MED1/Pol II DNA occupancy levels. Corresponding to Figure 2E and 2F. Data were generated from three biological replicates. Error bars represent SD of the mean. Unpaired t-test was used for statistical analysis. **, ***, ****, and ns denote p<0.01, p<0.001, and p<0.0001, respectively. **(B)** Venn diagram showing the unique and overlapped peak counts obtained from three NPC H3K27ac ChIP-seq datasets. Colors indicate different datasets (green: dataset GSM3163832; blue: dataset GSM3177720; orange: dataset GSE158382). Given GSE158382 contains the largest peak counts and includes most of the peaks from the other two datasets, we used peak information from this dataset to define active enhancers in our analysis. **(C)** Meta-analysis (left) of MED23 CUT&Tag read density around TSS within a ±3 kb window. Triplicates were performed for control (ctrl) and mutant (*MED27*$^{KI/FS}$) NPCs, with aggregated read density

plotted for each sample type. Genomic distribution (right) of MED23 differential occupying peaks (DOPs) between mutant and control NPCs. **(D)** Venn diagram (left) showing overlapped DEGs with concordant MED23 and Pol II DOPs at promoter regions. TF, transcription factors identified among the overlapped genes. On the right, bulk RNA-seq read counts of *EGR1* and occupying profiles of Pol II, MED1, and MED23 at the *EGR1* promoter. **(E)** Venn diagram showing overlapping DEGs identified using MED1 experimental data (see Figure 2D) and overlapping DEGs identified using MED23 experimental data (see Figure S2D). **(F)** Venn diagram demonstrating shared and unique Hi-C loops called by SIP in Ctrl (cyan) and *MED27*$^{KI/FS}$ (orange) samples, respectively. **(G)** Pie chart illustrating differential Hi-C loops between Ctrl and *MED27*$^{KI/FS}$ samples (|Log_2foldchange|>1, adjusted p<0.05) called by SIP. **(H)** GO analysis of genes within loops that were lost (top) or gained (bottom) in the mutant cells. The bubble size represents the number of genes associated with each term, and the color intensity of the bubble reflects the p value. GO terms related to gene transcription are highlighted with red arrows. See also Table S1. **(I)** Bar chat illustrating loop counts and heatmap showing loop strengths (loopability) for control and mutant NPCs analyzed by loop caller Mustache. APA scores were calculated to demonstrate enrichment of loops for each sample.

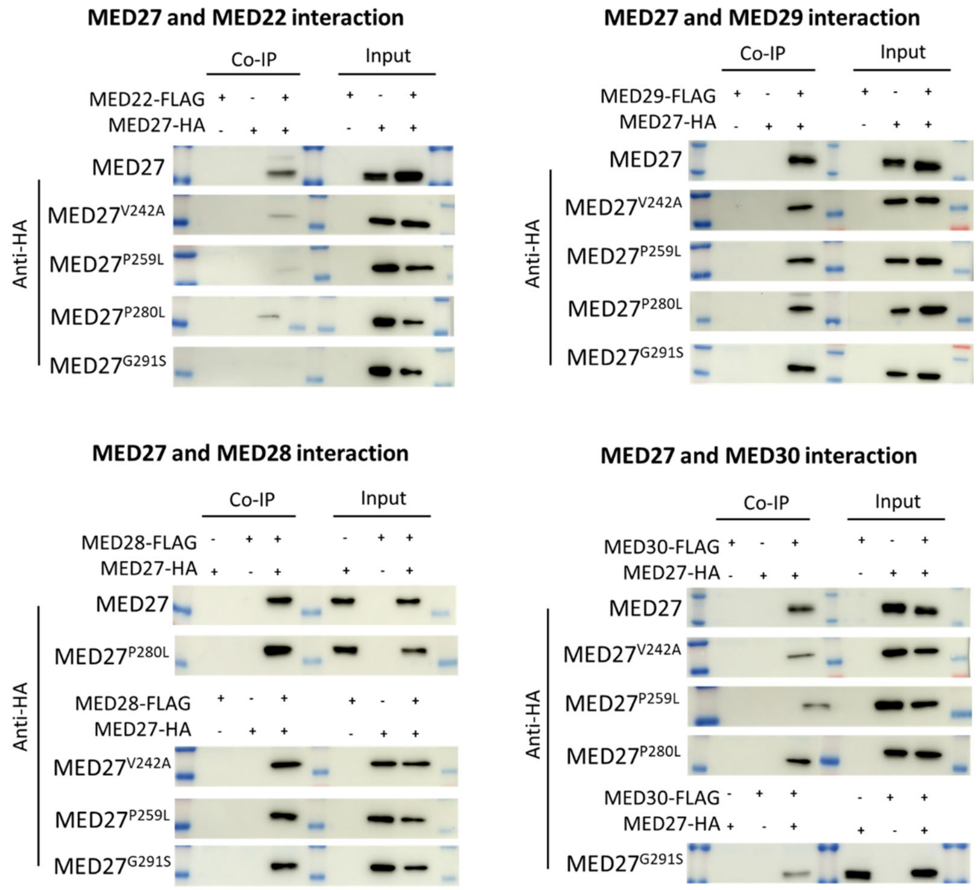

Figure S3 Co-IP experiments demonstrating the interactions between MED27 and MED20, MED22, MED28, MED29, and MED30, respectively.

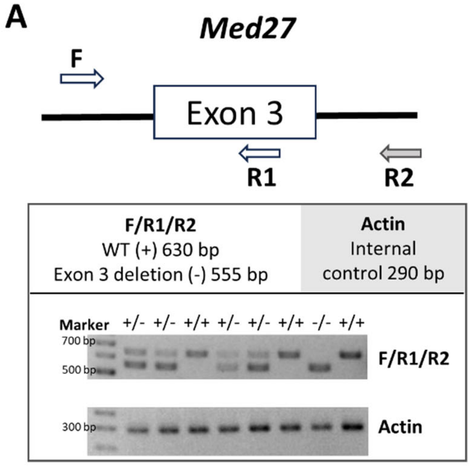

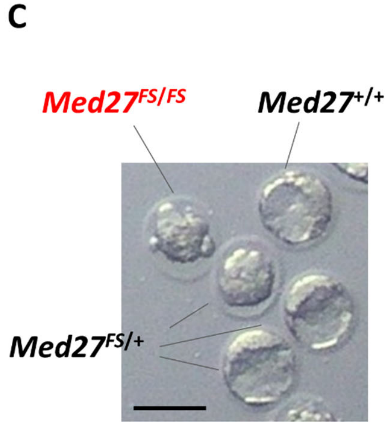

B

	Med27$^{+/+}$	*Med27*$^{FS/+}$	*Med27*$^{FS/FS}$	Total	Number of litters
P0 neonate	189	312	0	501	30
E13.5	2	7	0	9	1
E11.5	2	8	0	10	1
E9.5	0	2	0	2	1
E8.5	1	3	0	4	1
E7.5	11	21	0	32	5
E6.5	5	2	0	7	3
E3.5	9	17	6	32	5
**Total**	219	372	6	597	47

Figure S4 Genotyping strategy and lethality stage of *Med27* germline KO mice. (A) Diagram showing exon 3 of the *Med27* gene and the genotyping strategy. An actin (*Actb*) genomic region was used as the PCR control for each sample. F/R1/R2 are PCR primers. WT, wildtype. (B) Progeny counts generated from *Med27*$^{FS/-}$ heterozygote mating. The expected Mendelian ratio for each genotype is 1:2:1. (C) Representative genotyped embryos at E3.5, scale bar = 100µm.

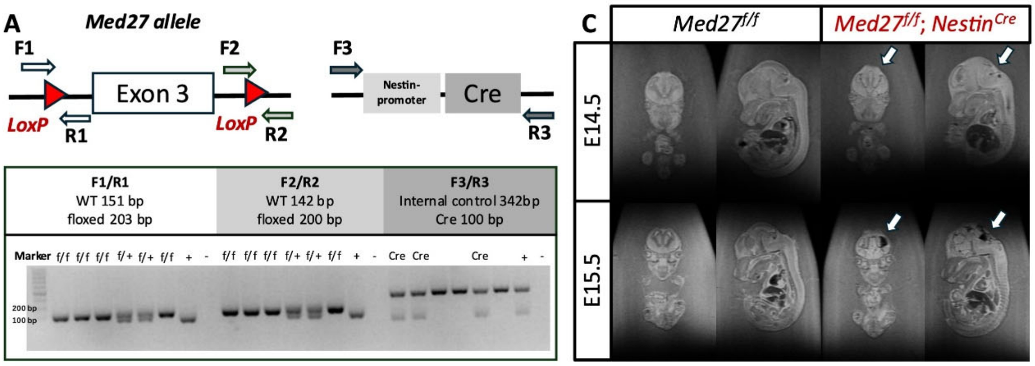

B

	$Med27^{f/f}$	$Med27^{f/+}$	$Med27^{f/+}$; $Nestin^{Cre}$	$Med27^{f/f}$; $Nestin^{Cre}$	Total	Number of litters
P0 neonate	6	10	11	0*	27	6
E17.5	0	2	2	1	5	1
E15.5	7	9	7	7	30	5
E14.5	2	5	6	5	18	3
E13.5	7	2	7	9	25	3
**Total**	22	28	33	22	105	18

Figure S5 Genotyping strategy and lethality stage of *Med27* CNS LoF mice. (A) Diagram showing the floxed *Med27* allele and the genotyping strategy. An interleukin 2 (*Il2*) genomic region was used as the PCR control for each sample. F1/R1, F2/R2, and F3/R3 are PCR primer pairs. WT, wildtype. (B) Progeny counts generated from $Med27^{f/f}$ mating with $Med27^{f/+}$; $Nestin^{Cre}$ mice. The expected Mendelian ratio for each genotype is 1:1:1:1. (C) MRI scan of *Med27* CNS KO embryos showing T2-weighted fast spin echo sequence (FSE) with a 30.00 mm x 30.00 mm field of view on the sagittal plane and coronal plane, respectively; slice thickness = 0.3 mm, total slice = 9, number of average = 1. White arrows indicate the hemorrhaging in lateral ventricles and hindbrain region.

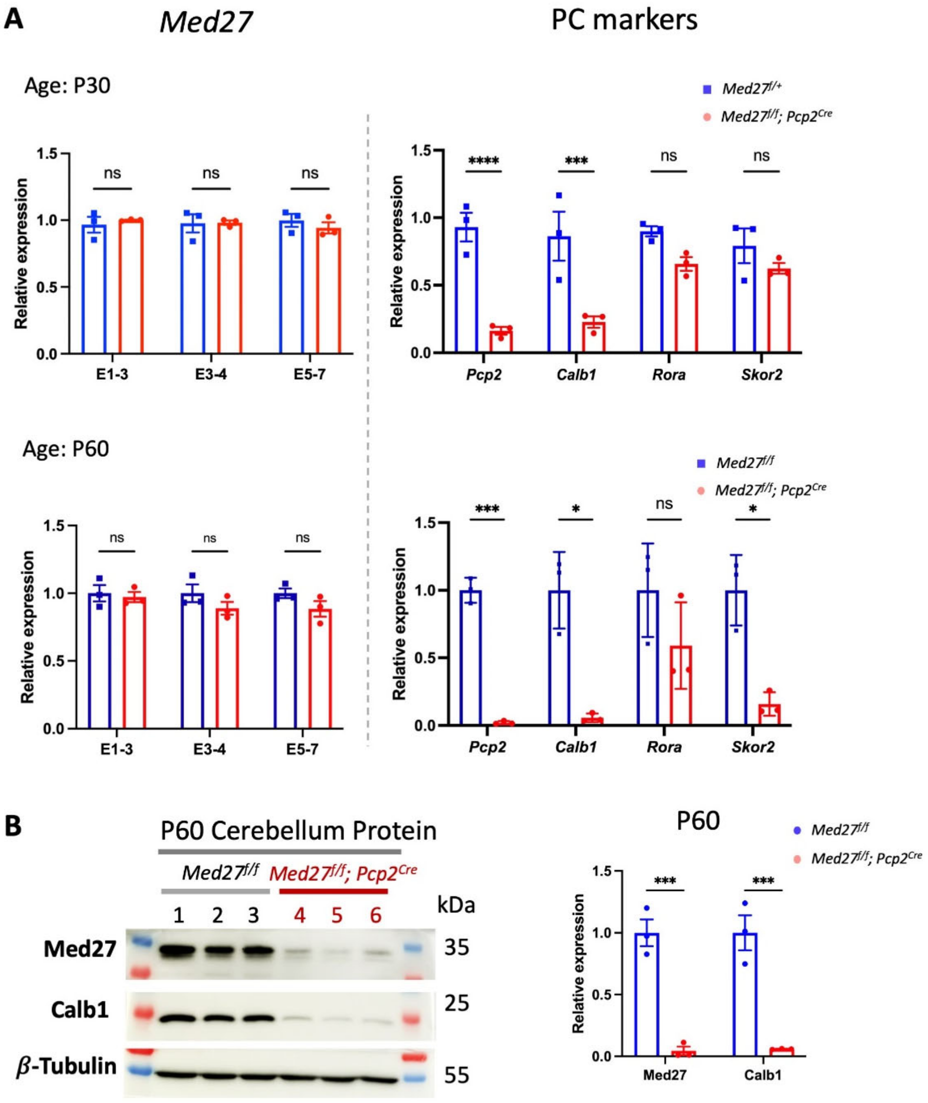

Figure S6 Expression levels of *Med27* and PC marker genes in *Med27* cerebellum LoF mice. (A) qPCR results of *Med27* (three sets of primers designed to target different regions, including exons (E) 1-3, E3-4, and E5-7) and PC marker genes *Pcp2*, *Calb1*, *Rosa*, and *Skor2* (n=3). (B) Western blot images and quantifications of the protein levels of Med27 and Calb1 in P60 mice (n=3). For statistical analysis, two-way ANOVA was used in a, two-tailed Student's T-test was used in b; *, ***, **** and ns denote $p<0.05$, $p<0.001$, $p<0.0001$ and $p>0.05$, respectively.

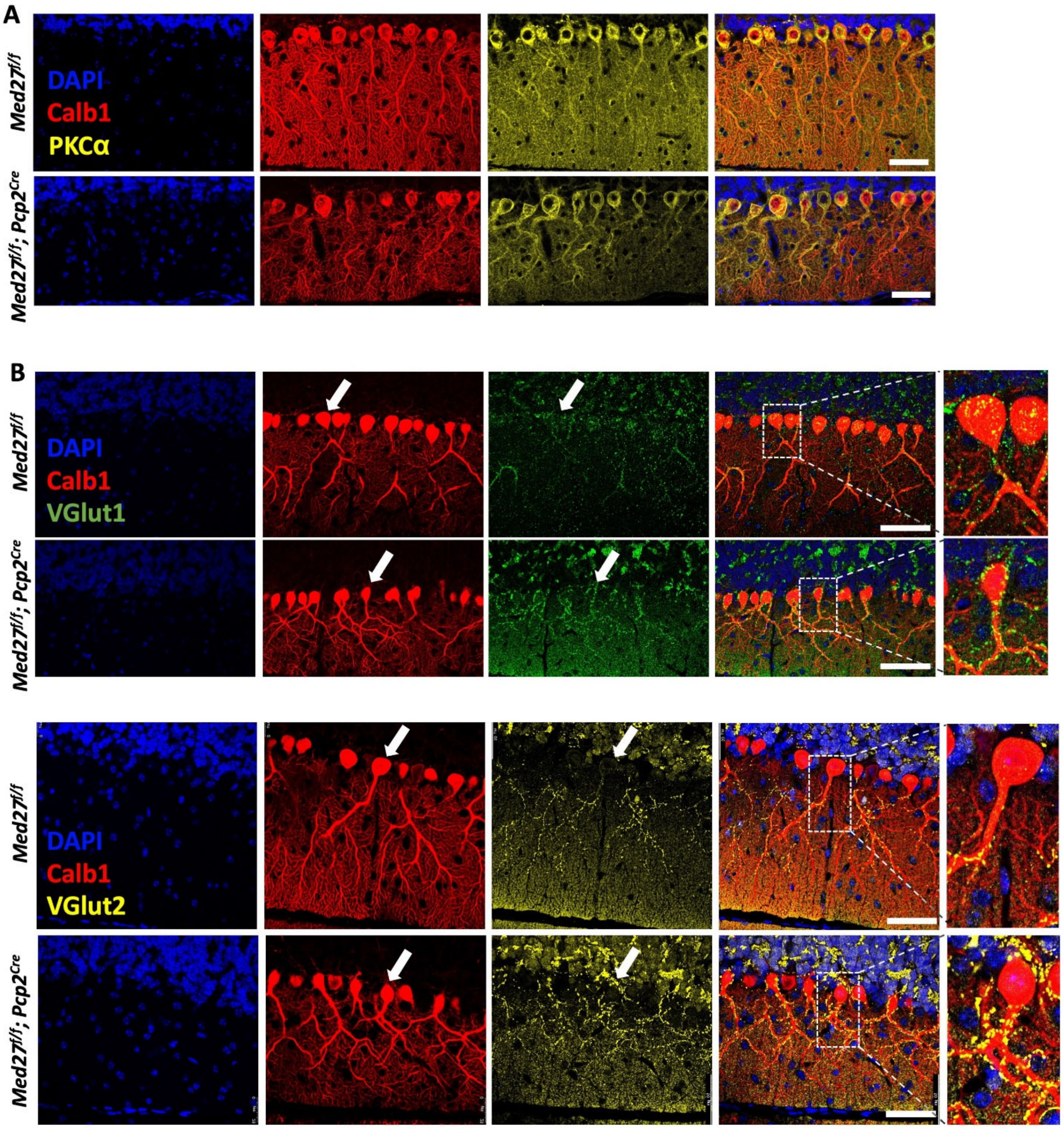

Figure S7 Abnormal PCs in *Med27* cerebellum LoF mice. (A) Representative IF staining images of PCs markers Calb1 and PKCα in P21 mice. (B) Representative IF images of presynaptic markers VGlut1 (vesicular-glutamate transporter 1 for parallel fibers - PC synapse) and VGlut2 (vesicular-glutamate transporter 2 for climbing fiber - PC synapse), scale bar = 50 µm.

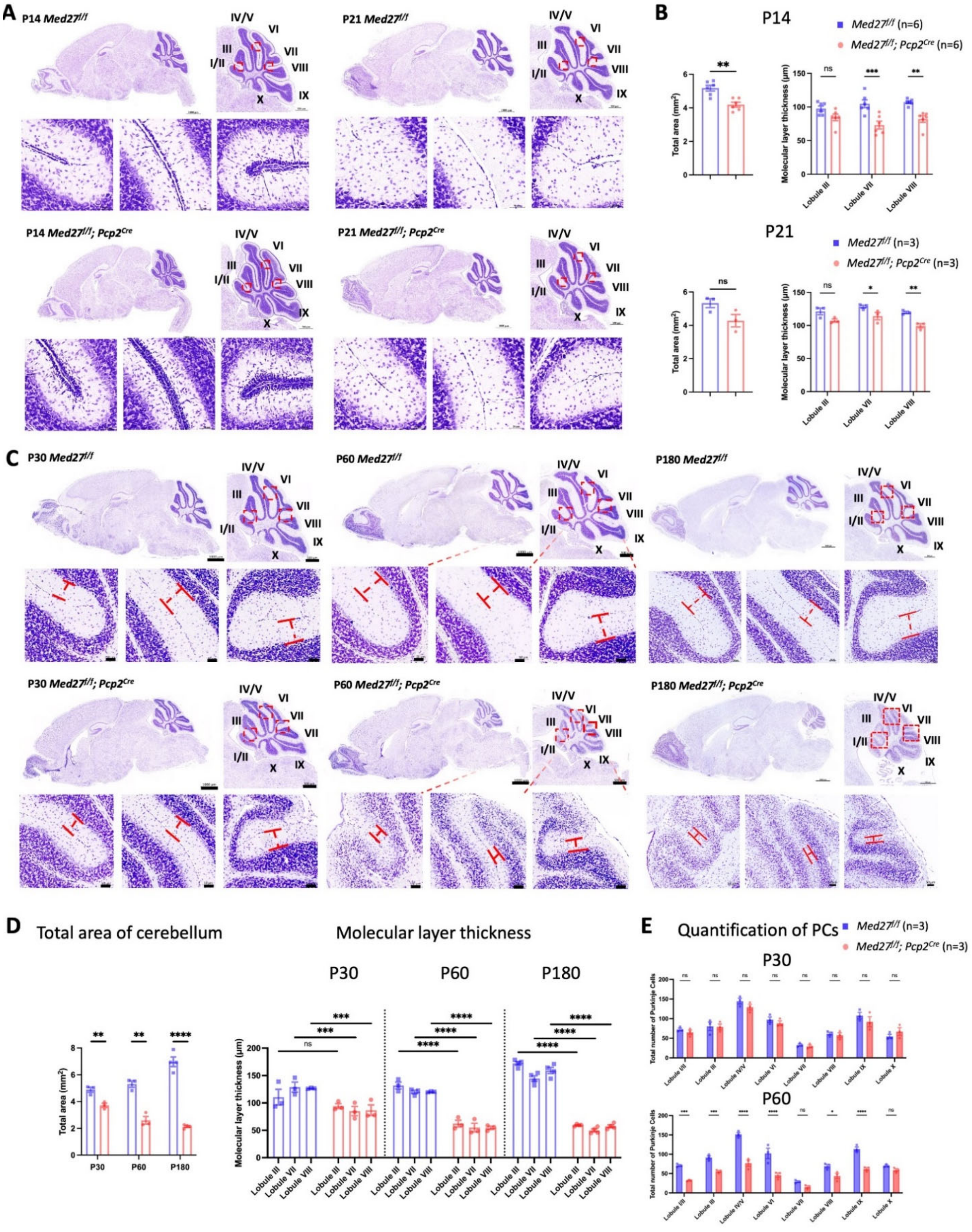

Figure S8 Cerebellar atrophy in *Med27* cerebellum LoF mice. (A) Representative Nissl staining images and (B) quantification comparisons of the total cerebellar area and molecular layer thicknesses in lobule III, VII and VIII based on Nissl staining results at P14 and P21. (C) Representative Nissl staining images and (D) quantification comparisons showing cerebellar atrophy from P30 to P180 (n=3 to 4). (E) Quantification of total PCs counts in all cerebellar lobules at P30 and P60. For statistical analysis, two-tailed Student's T-test and two-way ANOVA with Tukey's multiple comparison were used; *, **, ***, **** and ns denote $p<0.05$, $p<0.01$, $p<0.001$, $p<0.0001$ and $p>0.05$, respectively.

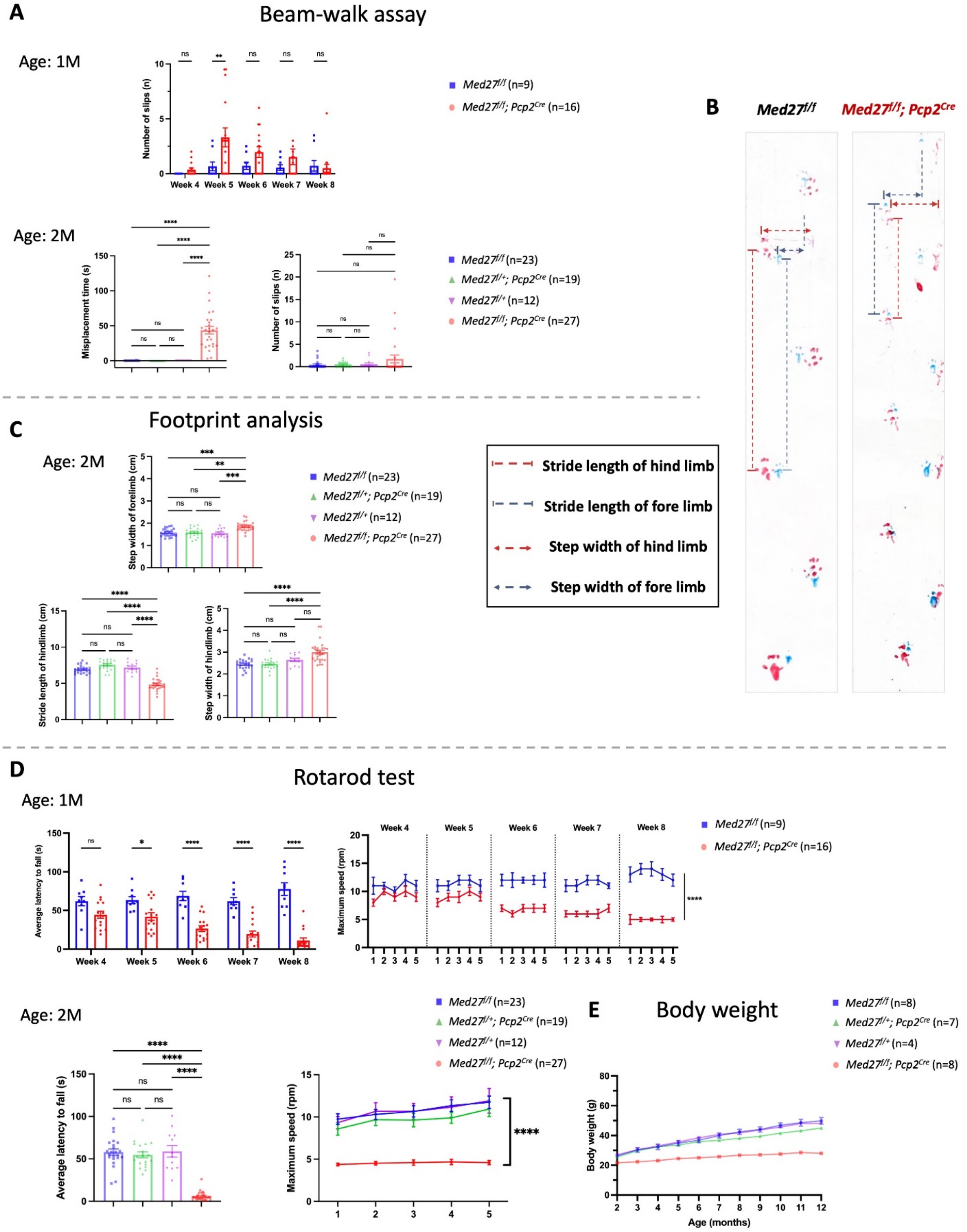

Figure S9 Progressive motor deficits in *Med27* cerebellum LoF mice. (A) Beam-walk assay results of number of slips in P30 and P60 mice. (B) Representative footprints in P60 mice. Hind paws were painted in red and fore paws in blue. (C) Quantification results of the stride length and step width of footprints. (D) Quantification results of the latency to fall and maximum speed of the rotating beam by rotarod test. (E) Body weight changes of male mice over time. For statistical analysis, one-way and two-way ANOVA were used; *, **, ***, **** and ns denote p<0.05, p<0.01, p<0.001, p<0.0001 and p>0.05, respectively.

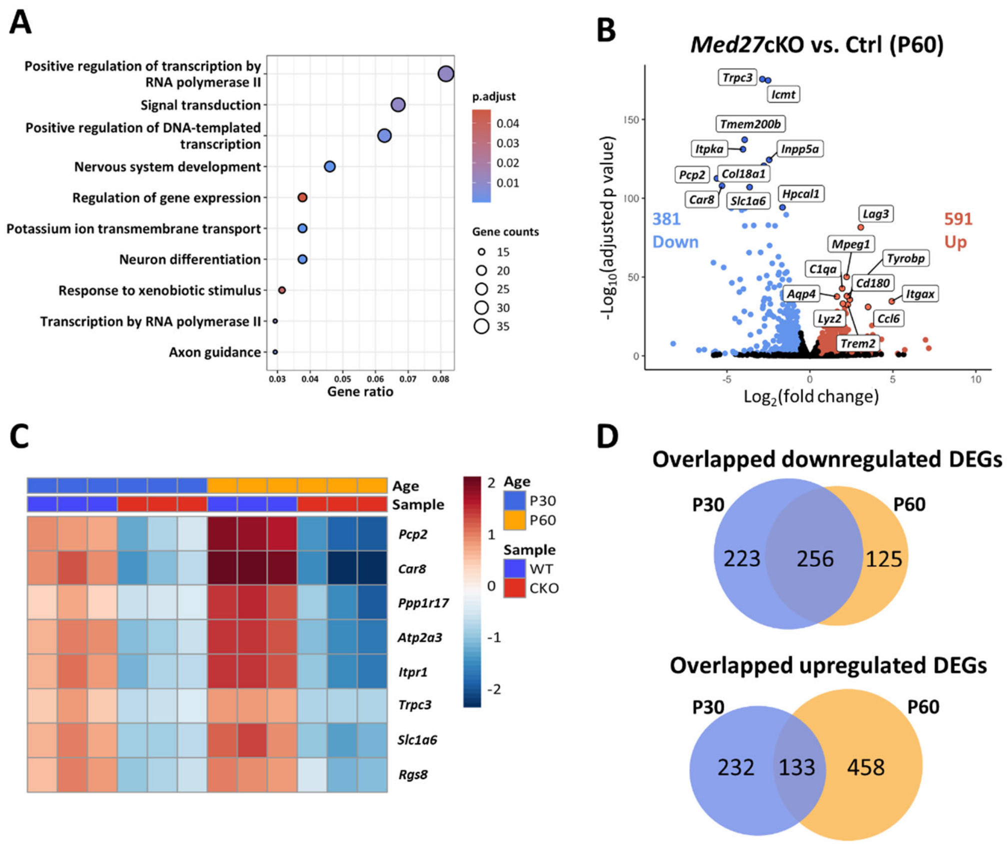

Figure S10 Transcriptomic dysregulation due to LoF of *Med27* in the cerebellum illustrated by bulk RNA-seq. (A) GO analysis of downregulated DEGs identified in Figure 6B with the top enriched biological processes shown. The bubble size corresponds to the number of genes each term involved. The color intensity of the bubble corresponds to adjusted p value. (B) Volcano plot showing bulk RNA-seq result comparing P60 *Med27*$^{f/f}$;*Pcp2*^{Cre} and their littermate control mice's cerebellum transcriptomic profiles. 381 downregulated and 591 upregulated DEGs were identified ($|Log_2foldchange|>0.58$, adjusted $p<0.05$). Gene symbols of the top 10 down- and upregulated DEGs were labeled. (C) Heatmap of expression levels for genes related to PCs or cerebellum development. (D) Venn diagrams illustrating overlapped down DEGs and up DEGs between P30 and P60 bulk RNA-seq results.

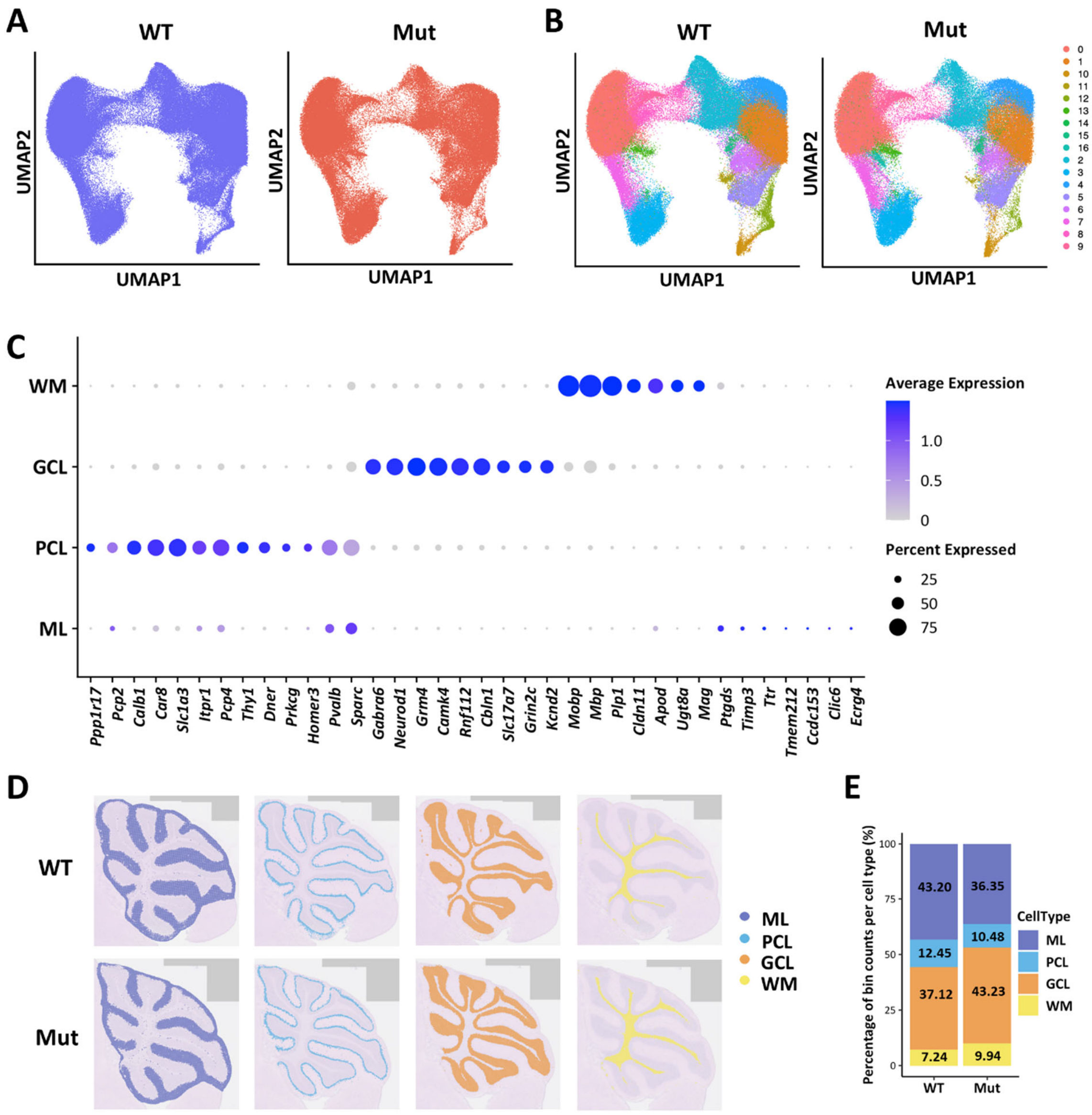

Figure S11 Transcriptomic dysregulation due to LoF of *Med27* in the cerebellum illustrated by sc-ST. (A) UMAP embeddings of the Visium HD data after batch correction. (B) UMAP embeddings of the Visium HD data colored by unsupervised clustering. (C) Dot plot of representative marker genes in each cell type. Color density represents the average expression level and dot size represents the expressed percentage of a marker gene detected within a specific cell type. ML, molecular layer; GCL, granule cell layer; PCL, Purkinje cell layer; WM, white matter. (D) Spatial localization of each cell type in the WT and Mut sample, respectively. (E) Bar plot demonstrating the proportions of each cell type in the WT and Mut sample, respectively.

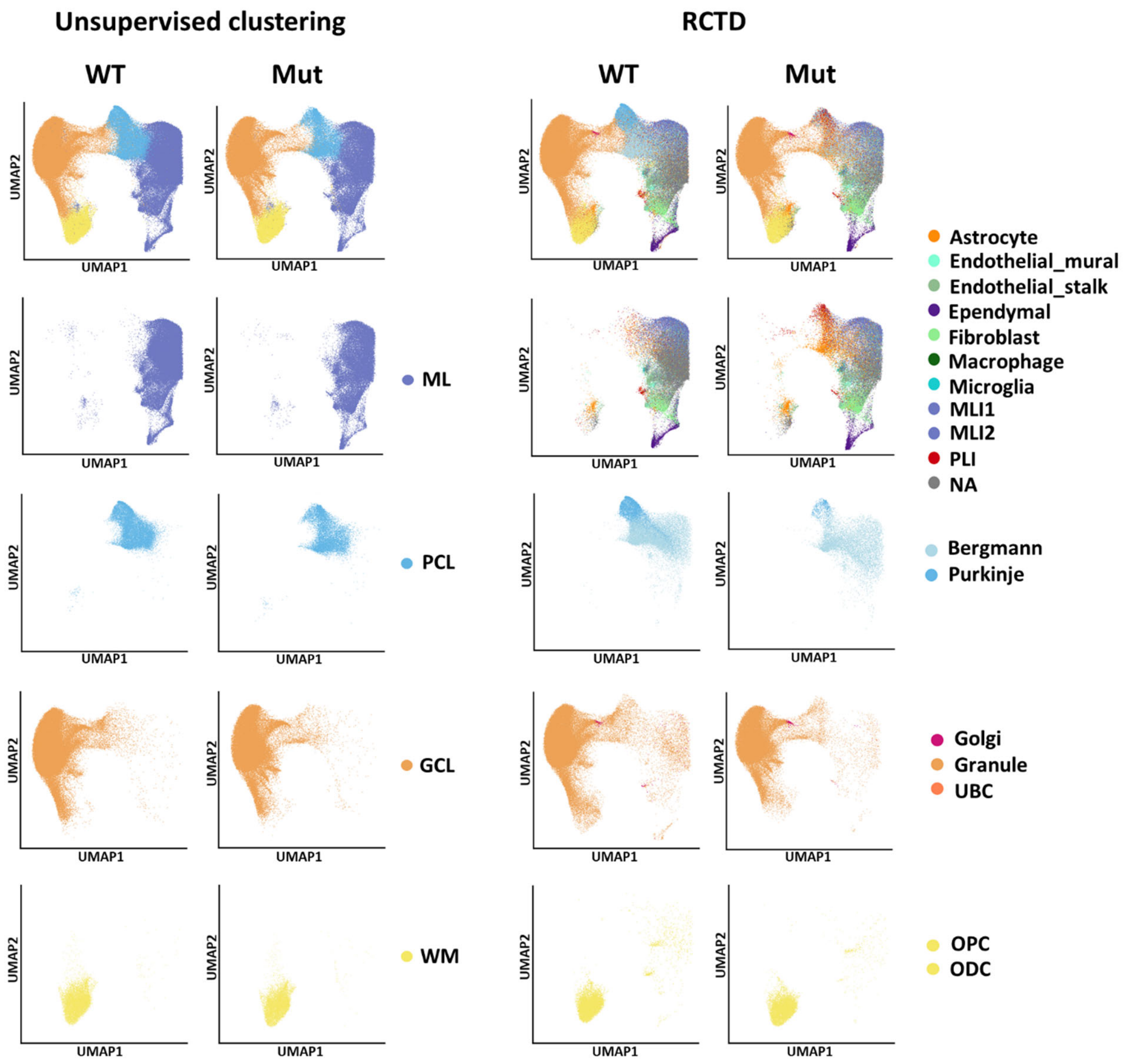

Figure S12 Comparison of annotated cell clusters identified by unsupervised clustering and the RCTD-based method. (Left) UMAP embeddings of each cell type (ML, PCL, GCL, WM) for the WT and Mut sample generated using the unsupervised clustering method. (Right) UMAP embeddings of the corresponding cell types obtained from the RCTD-based method. ML, molecular layer; GCL, granule cell layer; PCL, Purkinje cell layer; WM, white matter; MLI, molecular layer interneurons; PLI, Purkinje layer interneurons; UBC, unipolar brush cell; OPC, oligodendrocyte precursor cell; ODC, oligodendrocyte cell.

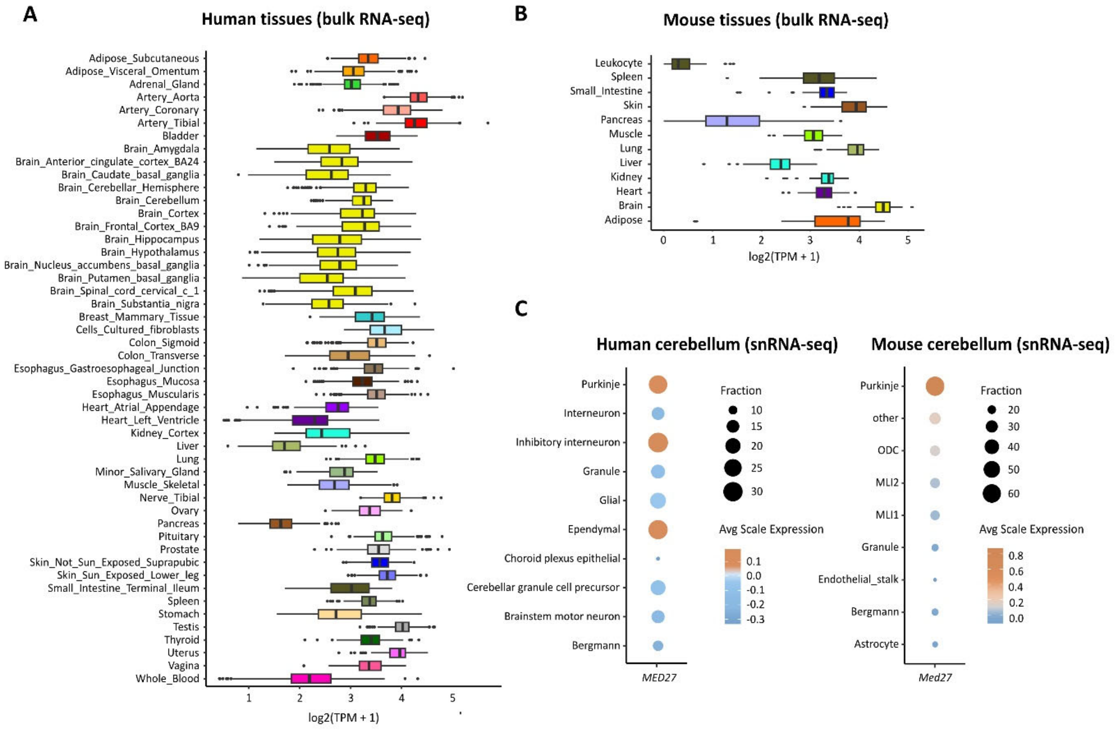

Figure S13. ***MED27*/*Med27* mRNA expression levels in human and mouse tissues. (A)** *MED27* expression levels across various human tissues. Bulk RNA-seq data were obtained from the GTEx project (GTEx Analysis V10 release, https://www.gtexportal.org/). The Y-axis denotes 49 human adult tissue types, each containing over 77 samples. **(B)** *Med27* expression levels across various mouse tissues. Bulk RNA-seq data were obtained from four independent datasets in the Bgee database (https://www.bgee.org/, experiment IDs ERP108893, SRP028336, SRP181218, and SRP199494). The X-axis in both (A) and (B) plots represents mRNA expression levels quantified as transcripts per million (TPM), normalized by transcript length and sequencing depth. (C) Bubble plots showing *MED27*/*Med27* expression levels in the human and mouse cerebellum. Single-nuclei RNA-sequencing (snRNA-seq) data were obtained from the dbGaP database (accession number phs001908.v2.p1) and GEO database (accession number GSE165371), respectively. Bubble size represents the mean percentage of *MED27*/*Med27* expression in each cell type, while bubble color indicates the mean scaled expression value derived from each cell type. MLI, molecular layer interneurons; ODC, oligodendrocytes; other, cell types with cell count less than 1%.

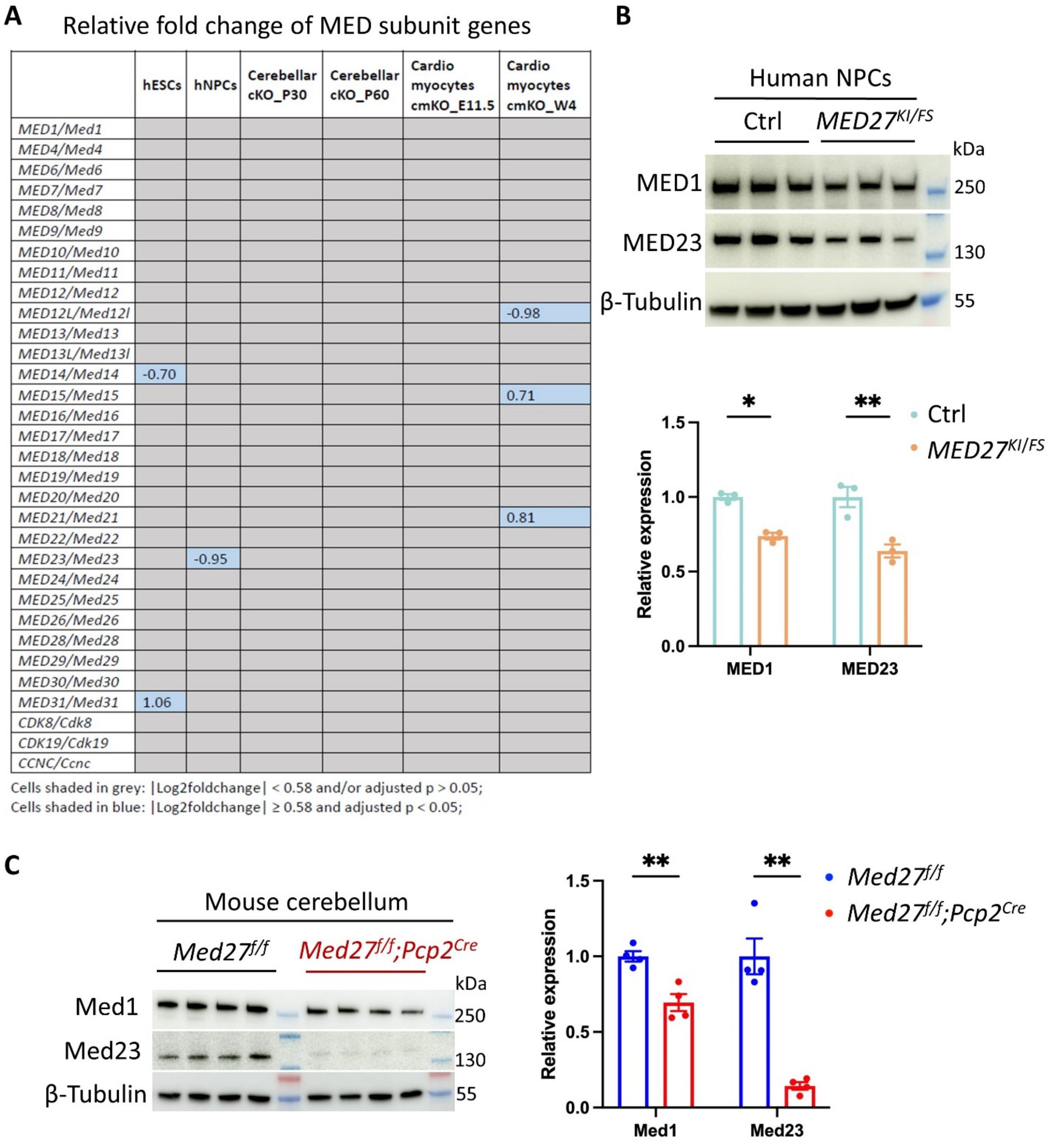

A Relative fold change of MED subunit genes

	hESCs	hNPCs	Cerebellar cKO_P30	Cerebellar cKO_P60	Cardio myocytes cmKO_E11.5	Cardio myocytes cmKO_W4
MED1/Med1						
MED4/Med4						
MED6/Med6						
MED7/Med7						
MED8/Med8						
MED9/Med9						
MED10/Med10						
MED11/Med11						
MED12/Med12						
MED12L/Med12l						-0.98
MED13/Med13						
MED13L/Med13l						
MED14/Med14	-0.70					
MED15/Med15						0.71
MED16/Med16						
MED17/Med17						
MED18/Med18						
MED19/Med19						
MED20/Med20						
MED21/Med21						0.81
MED22/Med22						
MED23/Med23		-0.95				
MED24/Med24						
MED25/Med25						
MED26/Med26						
MED28/Med28						
MED29/Med29						
MED30/Med30						
MED31/Med31	1.06					
CDK8/Cdk8						
CDK19/Cdk19						
CCNC/Ccnc						

Cells shaded in grey: |Log2foldchange| < 0.58 and/or adjusted p > 0.05;
Cells shaded in blue: |Log2foldchange| ≥ 0.58 and adjusted p < 0.05;

Figure S14. Expression levels of other MED subunits. (A) Relative fold changes in the expression of MED subunit genes (excluding *MED27*/*Med27*) as determined by bulk RNA-seq. hESCs, human embryonic stem cells; hNPCs, human neuronal progenitor cells; cKO, cerebellar-specific conditional knockout of *Med27* in mice; cmKO, cardiomyocyte-specific knockout of *Med27* in mice; P30 and P60, postnatal days 30 and 60; E11.5, embryonic day 11.5; W4, 4 weeks of age. **(B)** Representative Western blot image and quantification of the MED1 and MED23 expression levels in human NPCs. **(C)** Representative Western blot image and quantification of the Med1 and Med23 expression levels in the cerebellum of cKO mice at P60 (n=4). In (B) and (C), β-Tubulin was used as the endogenous control. Statistical analysis was performed using two-tailed student's T-tests. * and ** denote $p<0.05$ and $p<0.01$, respectively.

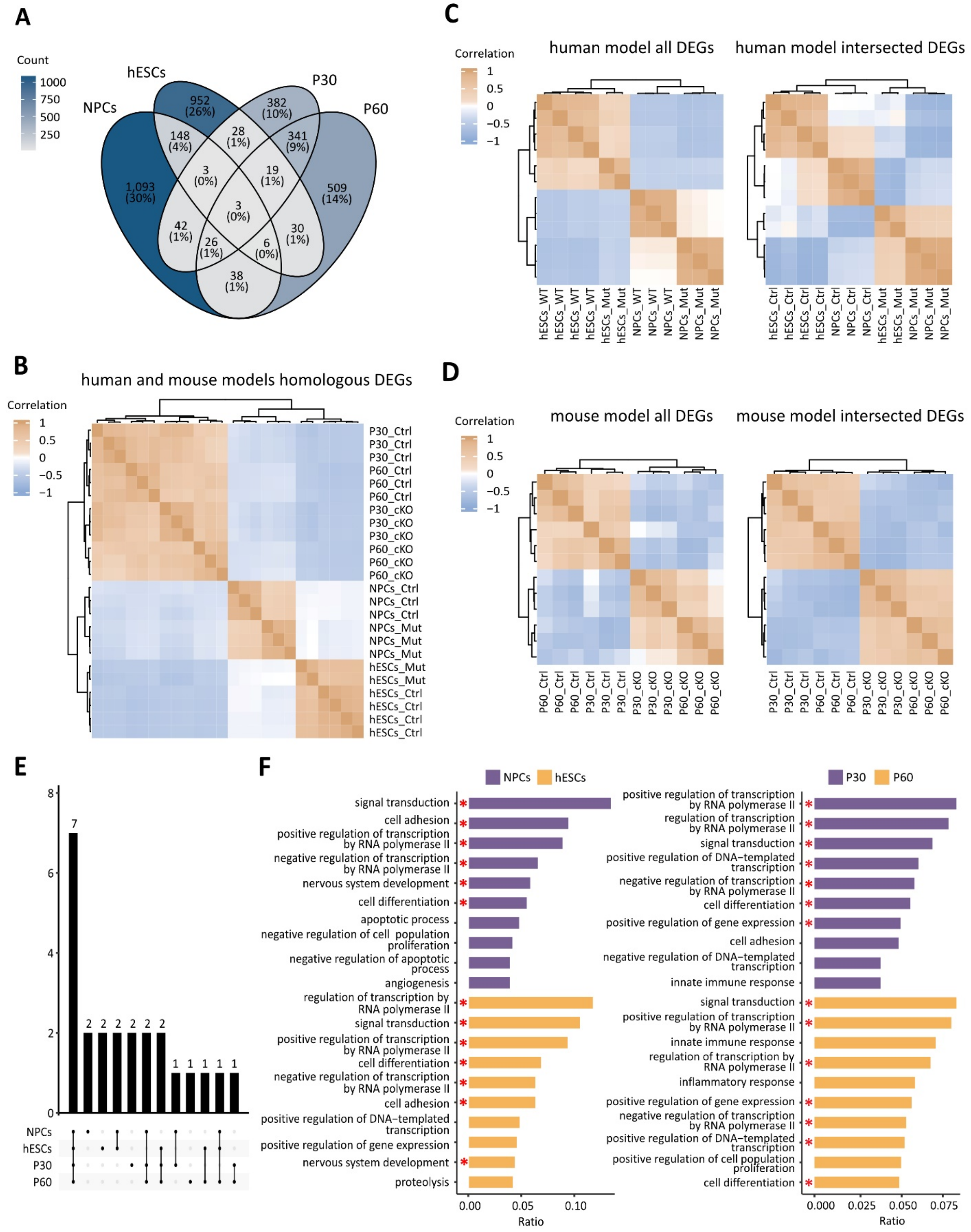

Figure S15. Comparison of DEGs across human and mouse samples. (A) Venn diagram illustrating the overlapping concordant DEGs across the four sample types. Colors represent the number of overlapping DEGs. hESCs, human embryonic stem cells; NPCs, human neuronal progenitor cells; P30 and P60, *Med27*-cKO mouse cerebellar tissues at postnatal day 30 and day 60, respectively. **(B)** Heatmap showing clustering analysis based on all 3,369 overlapping homologous DEGs identified between human and mouse models. **(C)** Heatmap displaying clustering analysis of human samples using all 2,388 DEGs identified in hESCs

and NPCs samples (left) or the 160 intersected DEGs shared between these two cell types (right). **(D)** Heatmap showing clustering analysis of mouse tissues using all 1,427 DEGs identified in P30 and P60 samples (left) or the 389 intersected DEGs shared between these two stages (right) (see also Figure S10D). **(E)** UpSet plot illustrating the overlapping enriched terms from the top 15 biological process pathways identified by GO analysis of DEGs across the four sample types. **(F)** Top 10 biological process pathways enriched from GO analysis for each sample type. Identical GO terms shared between hESCs and NPCs, or between P30 and P60 mouse tissues, are marked with a red asterisk.

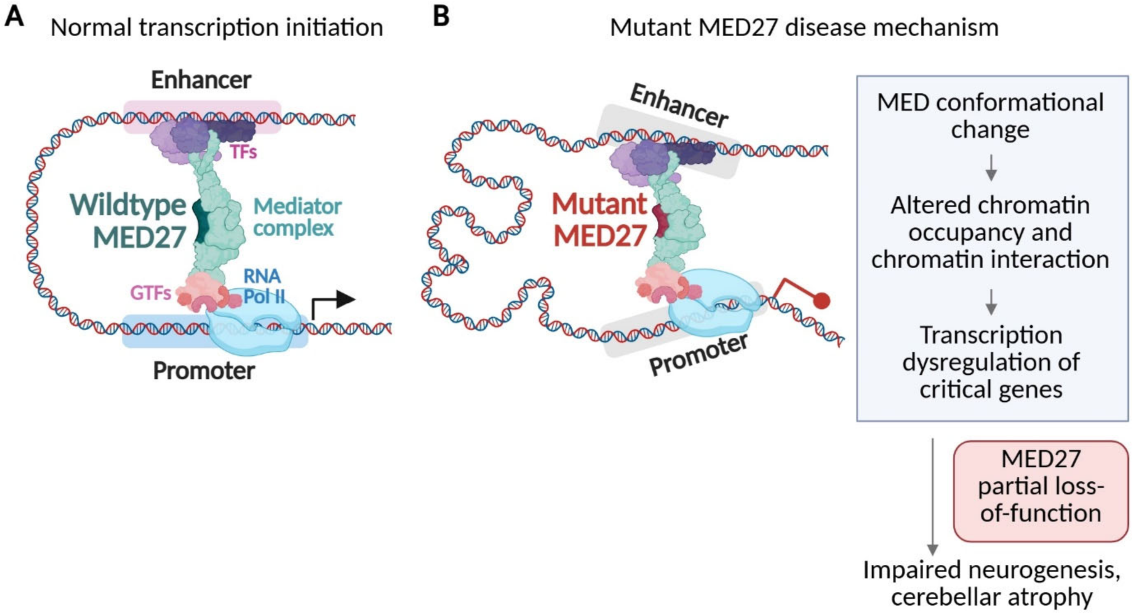

Figure S16 Illustration of MED27 function as a Mediator complex subunit. (A) Normal transcription initiation process (adopted from Reference 5). TFs, transcription factors; GTFs, general TFs. (B) MED27 disease pathogenicity mechanism proposed in this study. Created with BioRender.com.

Table S1. Overlapped DEGs with concordant MED and Pol II DOPs. Datasheet 1 corresponds to the overlapped DEGs in Figure 2E, datasheet 2 corresponds to Figure 2F, datasheet 3 corresponds to Figure S2D, and datasheet 2 corresponds to Figure S2H.

Table S2. AP-MS results

Table S3. Antibody and primer lists

Table S4. Clinical presentations of MED patients

Movie S1. Movement recording of one pair of littermates at P45 (*Med27*$^{f/f}$;*Pcp2*^{Cre} and *Med27*$^{f/f}$). The *Med27*$^{f/f}$;*Pcp2*^{Cre} mouse exhibited abnormal gait coordination, imbalanced posture, and lower body weight compared to its control littermate.

Materials and Methods

Cell culture

H1 hESCs were cultured on Geltrex-coated 6-well plates (Gibco #A1413301) in StemFlex medium (Gibco #A3349401) supplemented with 1% Normocin (InvivoGen #ant-nr-2), and the medium was refreshed daily. HEK-293T cells were cultured in DMEM (Gibco #12800017) supplemented with 10% fetal bovine serum (Gibco #10270106). All cells were maintained at 37°C in a humidified atmosphere with 5% CO_2 under sterile conditions and routinely tested for mycoplasma contamination.

Genome editing in hESCs

The single guide RNA (sgRNA) of base editor was designed following previous protocol [1]. The phosphorylated and annealed top and bottom oligo of sgRNA were ligated with digested MLM127 backbone. The Knock-out sgRNA was designed following CHOPCHOP (https://chopchop.cbu.uib.no/). The phosphorylated and annealed top and bottom oligo of sgRNA were ligated with digested pSpCas9(BB)-2A-GFP vector (PX458, Addgene# 48138). The constructed plasmids were sequenced by Sanger di-deoxynucleotide sequencing.

To generate *MED27*$^{KI/KI}$ hESCs, H1 cells were dissociated to single cells by Tryple. Washed cell pellet was resuspended in the entire Nucleofector Solution (Lonza# VPH-5012) and transferred to a cuvette for electroporation by the Lonza Nucleofector Transfection 2b Device. The entire Nucleofector Solution included 3μg pCMV_BE4max_P2A_GFP plasmid (Addgene# 112099) and 1μg sgRNA MLM127 plasmid in 82μl of Nucleofector Solution with 18μl of supplement. The *MED27*$^{KI/FS}$ hESCs were generated on top of *MED27*$^{KI/KI}$ hESCs, with similar experimental procedures except for the entire Nucleofector Solution being 4μg sgRNA PX458 plasmid in 82μl of Nucleofector Solution with 18μl of supplement. 48 hours post nucleofection, cells were sorted by BD FACSAria Fusion Cell sorter to select GFP positive cells. Single colonies of the sorted cells were genotyped by PCR and Sanger sequencing to select the ones with desired editing. A 12 bp deletion was noted at the sgRNA cut site for *MED27*$^{KI/FS}$ hESCs, which does not interfere with its normal protein coding frame. Predicted off-target sites were sequenced to rule out potential off-target effects. The sgRNA sequences, genotyping primers, and off-target detection primers sequences are listed in Table S3.

Neuronal progenitor cells (NPCs) differentiation

Cultured hESCs were digested into single cells using TrypLE (Thermo Scientific #12604021) and seeded into Ultra-Low Attachment 96-well plates (Corning #7007) at 5,000 cells per well. Plates were centrifuged at 110g for 6 minutes and cultured in StemFlex medium with 10 μM

ROCK inhibitor (SelleckChem, #S1049). Following differentiation medium was used per well sequentially: On Day 1, 100 μL of fresh StemFlex medium without ROCK inhibitor. On Day 2, 100 μL of differentiation medium 1 (DM1) composed of KnockOut DMEM (Gibco #10829018), 20% KnockOut Serum Replacement (Gibco #10828010), 1% GlutaMAX (Gibco #35050061), 1% NEAA (Gibco #11140050), 0.1% β-mercaptoethanol (Sigma #M3148), 1% Normocin, 10 μM SB431542 (PeproTech #3014193), and 0.1 μM LDN193189 (PeproTech #1066208). On Day 3, 75 μL DM1 and 25 μL differentiation medium 2 (DM2) composed of DMEM/F12 (Millipore #DF-042-B), 1% N2 Supplement (Gibco #17502048), 1% GlutaMAX, 1% NEAA, 1% Normocin, 10 μM SB431542, and 0.1 μM LDN193189. On Day 4 to 5, the ratio of DM1 to DM2 was gradually adjusted to 50:50 (Day 4) and 25:75 (Day 5), with 0.1 μM LDN193189. On Day 6 to 8, 100 μL DM2 containing 0.1 μM LDN193189. On Day 9, formed EBs were transferred to poly-L-ornithine (Sigma #P4957) and laminin (Sigma #L2020)-coated 12-well plates and cultured in DM2. On Day 10 to 15, the medium was refreshed daily with DM2 until neural rosette formation. Outer-layer cells were removed, and the rosette EBs were digested with Accutase (Millipore #SCR005) before being reseeded onto coated 6-well plates in differentiation medium 3 (DMEM/F12, 0.5% N2, 1% B27, Gibco, #12587010; 1% GlutaMAX; 1% NEAA; 1% Normocin). After two passages, cells were selected based on two positive markers (CD24, BD, #555428; CD184, BD, #555976) and two negative markers (CD44, BD, #555478; CD271, BD, #562562) by the BD FACSAria Fusion Cell Sorter and NPCs were collected post sorting.

Spontaneous Differentiation

hESCs cells were digested with TrypLE and seeded onto Ultra-Low Attachment 24-well plates (Corning, #3473) in spontaneous differentiation medium 1 (DMEM/F12, 20% KnockOut Serum Replacement, 1% NEAA, 2 mM GlutaMAX, 0.1 mM β-mercaptoethanol). The medium was refreshed every other day with gentle shaking. After 7 days, cells were transferred to gelatin-coated plates (Stem Cell #07903) and cultured in spontaneous differentiation medium 2 (DMEM, 10% FBS) for another 7 days. Differentiated cells were harvested and total RNA was extracted for RT-qPCR analysis.

RT-qPCR

Total RNA was extracted using TRIzol (Invitrogen, #15596018) and purified with the TriRNA Pure Kit (Geneaid, #FI17102). Reverse transcription was performed using the cDNA Synthesis Master Mix (Thermo Scientific, #M1662). qPCR was conducted on the QuantStudio™ 7 Flex Real-Time PCR System using SYBR Green Master Mix (Thermo

Scientific #A46112). At least three biological replicates were performed for each experiment. See Table S3 for primers used in this study.

Western Blot

Proteins were extracted with lysis buffer (25 mM Tris-HCl, 150 mM NaCl, 5 mM EDTA, 1% Triton X-100, 10% glycerol) supplemented with protease inhibitor cocktail (Bimake #B14001) and denatured at 95°C for 10 minutes. Samples (10 μg) were loaded onto 12% SDS-polyacrylamide gels, transferred to PVDF membranes, and blocked with 5% milk for 1 hour. Membranes were incubated with the primary antibody overnight at 4°C, followed by secondary antibody incubation for 2 hours at room temperature. Signals were visualized using the Amersham Cytiva ImageQuant 800 Imaging System. At least three biological replicates were performed for each experiment. See Table S3 for antibodies used in this study.

IF staining in cells

Cells were fixed with 4% paraformaldehyde for 20 minutes, washed with PBS, permeabilized with 0.1% Triton X-100 for 20 minutes, and blocked with 1% BSA (ExCell #S-BSA00100) and 4% goat serum (Sigma #G9023) for 1 hour. Cells were incubated with the primary antibody overnight at 4°C and then incubated with the secondary antibody for 2 hours, followed by DAPI staining (Solarbio #C0060) for 20 minutes. Coverslips were mounted with mounting medium (Invitrogen #00495802) and images were captured using a Leica TCS SP8 Confocal Microscope. See Table S3 for antibodies used in this study.

Bulk RNA sequencing

Illumina paired-end sequencing reads were trimmed using Trimmomatic v0.39 with the adapter reference TruSeq3-PE.fa:2:30:10. Reads were then aligned to the human reference genome (GRCh38) or the mouse reference genome (GRCm39) using Hisat2 v2.2.1. Read counts were generated with featureCounts v2.0.4 and subsequently used for differential gene expression analysis with the DESeq2 package v1.42.1 in R. Differentially expressed genes (DEGs) were identified using an adjusted P-value cutoff of 0.05 and absolute log2 fold change thresholds of 1 for human cell samples and 0.58 for mouse tissue samples. Volcano plots and heatmaps were created using the R packages ggplot2 v3.5.1 and pheatmap v1.0.12, respectively. GO analysis was conducted using the Database for Annotation, Visualization, and Integrated Discovery (DAVID) online tool (http://david.ncifcrf.gov/).

CUT&Tag sequencing

Sample processing

The CUT&Tag assay was performed following manufacture's protocol (Vazyme #TD904 kit). Briefly, 10,000 cells were collected; their nuclei were extracted and fixed with 0.1%

formaldehyde, then neutralized with glycine. Nuclei were subsequently incubated with ConA Beads Pro, followed by incubating with MED1 (Bethyl Lab #A300-793A), MED23 (Bethyl Lab #A300-425A), or Pol II (Abcam #ab26721) primary antibody overnight at 4°C. Secondary antibody incubation was then performed for 1 hour, after which nuclei were treated with Hyperactive pA/G-Transposon Pro provided in the kit. Subsequently, DNA was extracted, libraries were prepared, and sequencing was performed on an Illumina NovaSeq platform. CUT&Tag qPCR was performed using the libraries with 20 times dilution. See Table S3 for primer sequences.

Data analysis

CUT&Tag sequencing reads were trimmed using Cutadapt v4.9 to remove adapter sequences and aligned to the human reference genome (hg38) using Bowtie2 v2.5.4 with the following parameters: --local --very-sensitive-local --no-unal --no-mixed --no-discordant --phred33 -I 10 -X 500. Peaks were identified using MACS2 v2.2.9 with the parameters -q 0.05 -f BAMPE --keep-dup=all. BigWig files for peak visualization were generated with deepTools bamCoverage v3.5.5 (--normalizeUsing RPKM). Peak distribution across transcription start site (TSS) regions was analyzed using deepTools programs computeMatrix (-b 3000 -a 3000), plotHeatmap, and plotProfile. Peaks were annotated with associated genes and genomic features using the R package ChIPseeker v1.40.0 with a defined TSS region. Differentially enriched peak sites were identified using the R package DiffBind v3.14.0 with a DESeq2-based analysis method, and overlaps between peak signals were determined using BEDTools. Active enhancer regions were defined based on previously published H3K27ac ChIP-seq data in NPCs (Figure S2B).

Hi-C

In situ Hi-C library preparation

The in situ Hi-C libraries were prepared following established protocols. Briefly, approximately 300,000 cells were cross-linked and processed to generate Hi-C libraries. Cells were lysed in 500 µl of ice-cold Hi-C lysis buffer [10 mM Tris-HCl (pH 8.0), 10 mM NaCl, 0.2% Igepal CA630, supplemented with complete proteinase inhibitors] and incubated on ice for 15 minutes. The lysate was centrifuged at 2500 × g for 5 minutes, and the pelleted nuclei were washed once with 500 µl of 1.25× NEBuffer 3.1. After discarding the supernatant, the nuclei were resuspended in 358 µl of 1.25× NEBuffer 3.1, and 11 µl of 10% SDS was added. The mixture was incubated at 37°C for 1 hour. Subsequently, 75 µl of 10% Triton X-100 was added to quench the SDS, followed by another incubation at 37°C for 1 hour. Chromatin digestion was performed by adding 100 units of DpnII restriction enzyme (NEB #R0543) and

incubating the samples at 37°C overnight. The enzyme was inactivated by heating the samples to 62°C for 20 minutes, followed by cooling to room temperature. The samples were then centrifuged at 2500 × g for 5 minutes, and the pelleted nuclei were resuspended in 50 µl of fill-in master mix [3.75 µl of 0.4 mM biotin-14-dATP, 1.5 µl of 1 mM dCTP, 1.5 µl of 1 mM dGTP, 1.5 µl of 1 mM dTTP, 2 µl of DNA polymerase I (5 U/µl, Klenow Fragment), and 1× NEBuffer 3.1]. The mixture was incubated at 23°C for 4 hours to fill in the restriction fragment overhangs and biotinylate the DNA ends. For ligation, a master mix [398 µl of water, 50 µl of 10× NEB T4 DNA ligase buffer, 1 µl of bovine serum albumin (50 mg/ml), 1 µl of T4 DNA ligase (400 U/µl)] was added, and the samples were incubated at 16°C overnight in a ThermoMixer C with intermittent shaking. After ligation, the nuclei were pelleted by centrifugation at 2500 × g for 5 minutes, and 380 µl of the supernatant was discarded. The pellets were resuspended in the remaining 120 µl of ligation mix, supplemented with 12 µl of 10% SDS and 5 µl of proteinase K (20 mg/ml), and incubated at 55°C for 2 hours with shaking at 1000 rpm. Following this, 15 µl of 5 M NaCl was added, and the reaction was incubated at 65°C for 16 hours. DNA was purified by phenol:chloroform extraction and ethanol precipitation, then resuspended in 130 µl of 0.1× TE buffer. The DNA was sheared to approximately 300 bp using a Covaris S220 instrument (intensity: 175 W, duty cycle: 10%, cycles per burst: 200, time: 150 seconds). Biotinylated DNA was captured using 10 µl of Dynabeads MyOne Streptavidin C1 beads (10 mg/ml; Life Technologies, 65001). The beads were resuspended in 23 µl of 10 mM Tris-Cl (pH 8.0), and library preparation was performed using the NEBNext Ultra II DNA Library Preparation Kit (NEB #E7645S). After magnetic separation and washing, the beads were resuspended in 20 µl of 10 mM Tris buffer and boiled at 98°C for 10 minutes. The eluted DNA was amplified using Phanta Master Mix (Vazyme #P511-01) for 10–13 PCR cycles, and the PCR products were purified with VAHTS DNA Clean Beads (Vazyme #N411-01). Finally, the libraries were sequenced on an Illumina HiSeq X Ten instrument using paired-end reads.

Hi-C data processing

Raw sequencing reads were processed using HiC-Pro (v3.1.0) [2]. The paired-end reads were aligned to the human reference genome (hg38) using a two-step mapping strategy. In the first step, reads were aligned end-to-end using Bowtie2 (v2.3.3.1) [3]. In the second step, reads spanning the ligation junction were identified and realigned to the reference genome. The LIGATION_SITE parameter was set to the DpnII restriction enzyme recognition sequence ‘GATCGATC’. After alignment, reads were merged and assigned to their corresponding restriction fragments. Reads that were unmapped, of low quality (Mapping Quality (MAPQ) <

10), singletons, or aligned to multiple locations were filtered out. Additionally, invalid fragments such as religation products, dangling ends, self-circles, dumped pairs, and PCR duplicates were removed to ensure high-quality data. To account for technical biases and enable comparative analysis across different conditions, the interaction matrices were normalized using the Iterative Correction and Eigenvector Decomposition (ICE) method [4].

To ensure a fair comparison between samples with varying numbers of valid pairs, we performed downsampling to normalize the data. Specifically, the mutant sample, which had a higher number of valid pairs, was downsampled to match the level of the control sample. This was achieved using the 'shuf -n' command, which randomly selects a subset of reads corresponding to the number of valid pairs in the mutant sample. To identify chromatin loops, valid pairs were first converted into the '.hic' format using hicpro2juicebox with Juicer Tools (v1.22.01) [2, 5]. Chromatin loops were then identified using two independent loop-calling algorithms. The first analysis was performed using SIP (v1.6.4) [6] with Juicer Tools (v1.19.02) [5], utilizing the parameters: -norm KR -res 10000 -g 3 -fdr 0.01 -mat 1000. The second analysis was conducted using Mustache (v1.3.3) [7] with the parameters: -r 10kb -pt 0.01. To conduct aggregate peak analysis (APA), we first converted .hic files into .cool format using hic2cool (v0.8.3; https://github.com/4dn-dcic/hic2cool). The .cool files were subsequently balanced using cooler (v0.10.2) [8], to account for systematic biases in the Hi-C data. Finally, APA plots and scores were generated using coolpup.py [9] with the parameter --norm_corners 10.

To identify chromatin loops shared between two conditions, we integrated loops from both conditions using the mergeBedpe() function from the R package hictoolsr (v1.1.2) [10]. This function utilized the DBSCAN (v1.1-11) algorithm with a resolution parameter (res = 10e3) to cluster and merge loops that either overlapped or were shifted slightly in their genomic coordinates. Loops merged under these criteria were classified as overlapping loops, while loops that did not merge were classified as shifted loops. To identify condition-specific loops, we quantified observed interaction counts from .hic files and computed the log2 fold change (log2FC) of interaction counts (control / mutant) for each loop using DESeq2 (v1.42.0) [11]. Loops with $|\log_2\text{FoldChange}| > 1$ were designated as condition-specific loops.

Co-IP

To construct expression plasmids, the coding sequences (CDS) of *MED20*, *MED22*, *MED28*, *MED29*, and *MED30* were cloned into the pCS2-FLAG vector, while the wildtype (WT) and mutant CDS of *MED27* were cloned into the pCMV-3HA vector, respectively, using the

ClonExpress Ultra One Step Cloning Kit (Vazyme #C117). All constructed plasmids were verified by Sanger sequencing. Primers used in plasmid construction are listed in Table S3.

To express these genes, 0.5 million HEK-293T cells were seeded in 6-well plates and 2μg plasmids was transfected in each well using Lip8000 (Beyotime# c0533) according to the manufacturer's instructions. For each Co-IP experiment, WT or mutant *MED27* was co-transfected with one of its neighboring MED subunits (*MED20*, *MED22*, *MED28*, *MED29*, or *MED30*), with single plasmid transfection as the controls. 48 hours post-transfection, cells were lysed in lysis buffer (0.5% NP-40, 1 mM EDTA, 1× TBS: 50 mM Tris, 150 mM NaCl) on ice for 20 minutes. Lysates were centrifuged under full speed at 4°C for 15 minutes. Anti-FLAG M2 Magnetic Beads (Sigma #M8823) were washed with TBS and incubated with the lysates at 4°C overnight. After thorough washing, proteins were eluted and analyzed by western blot.

AP-MS

Plasmid construction and expression

cSFB-tagged (S tag, FLAG epitope tag, and streptavidin-binding peptide tag) pMH-SFB vector was constructed as previously reported [12]. WT and mutant (p.P280L) coding sequences of *MED27* were respectively cloned into the SFB-tagged destination vector using the ClonExpress® II One Step Cloning Kit (Vazyme). All constructs were confirmed by Sanger sequencing. Backbone, WT MED27, and mutant MED27 plasmids were expressed in the HEK-293T cells using polyethylenimine (MCE) as the transfection reagent, respectively. Twenty-four hours post-transfection, cells stably expressing SFB-tagged MED27 proteins were selected by culturing in medium supplemented with 2ug/mL puromycin (Gibco) for 3 days. Expression of the conjugated MED27 proteins was validated by Western blot and IF staining. Cells were further expanded in complete culture medium for the subsequent affinity purification experiments.

Affinity purification

To isolate SFB-tagged proteins, transfected 293T cells were harvested, lysed in the NETN buffer (20 mM Tris–HCl [pH 8.0], 100 mM NaCl, 1 mM EDTA and 0.5% Nonidet P-40) containing protease inhibitors (Selleckchem) for 30 minutes (min) on a shaker at 4°C. The lysates were centrifuged at 14 krpm and 4°C for 20 min, and the resulting supernatant 1 were collected and stored on ice. The remaining pellets were further lysed in the TurboNuclease buffer (50mM Tris–HCl [pH 8.0] and 1mM MgCl2) containing TurboNuclease (Sigma) and protease inhibitors for 40 min in a 37°C water bath with occasional inversion until the solutions turned white. The resulting white solutions were centrifuged at 14 krpm and 4°C for

20 min to collect the supernatant 2. Supernatant 1 and 2 were subsequently combined and centrifuged at 14 krpm and 4°C for an additional 20 min. S-protein beads (MCE) were washed twice with 1 mL of NETN prior to incubation with the protein lysates. After mixing with the protein lysates, the beads were rotated at 4°C for 4 hours (h) for protein capture. Afterwards, the beads were pelleted by centrifugation at 5 krpm for 1 min and the supernatant was discarded. The beads were then washed three times with the NETN buffer and the resin-bound proteins were eluted by boiling in 2× SDS buffer for 15 min. Eluted proteins were analyzed and confirmed by SDS-PAGE followed by Western blot.

Preparation of peptides for MS analyses

Beads were washed with 1 mL of 50mM ammonium bicarbonate (NH_4HCO_3) buffer. After centrifugation at 1000rpm for 2 min, the supernatant was discarded. The samples were then reduced with 2μL of 0.5M Tris (2-carboxyethyl) phosphine (TCEP) at 37°C for 60 min and alkylated with 4μL 1M iodoacetamide (IAM) at room temperature for 40 min in darkness. Fivefold volumes of cold acetone were added to precipitate protein at -20°C overnight. After centrifugation at 12,000g at 4°C for 20 min, the pellets were washed twice by 1mL of pre-chilled 90% acetone aqueous solution. Then the pellets were re-suspended with 100μL 100mM triethylammonium bicarbonate (TEAB) buffer. Trypsin was added at 1:50 trypsin-to-protein mass ratio and incubated at 37°C overnight. The peptide mixtures were desalted by the Pierce C18 Spin Tips and subsequently dried using a speed vacuum concentrator.

MS data acquisition

Digested samples were analyzed on the TOF HT, an ion-mobility spectrometry quadrupole time of flight mass spectrometer (Bruker Daltonik, Bremen, Germany) equipped with the Vanquish Neo UHPLC liquid chromatography system (Thermo Fisher Scientific, MA, USA). Samples were reconstituted in 0.1% FA and 200 ng peptide was separated by the AUR3-15075C18 column (15 cm length, 75 μm i.d, 1.7 μm particle size, 120 Å pore size, IonOpticks) with a 20 min gradient starting at 4% buffer B (80% ACN with 0.1% FA) followed by a stepwise increase to 28% in 15 min, 90% in 1.5 min and stayed there for 3.5 min. The column flow rate was maintained at 300 nL/min with the column temperature of 50°C. Data-independent analysis (DIA) data was acquired in the diaPASEF mode, which was defined as 24 × 25 Th precursor isolation windows over 400-1000 m/z. To adapt the MS1 cycle time, the repetitions were set to 3 steps in the 8-scan diaPASEF scheme in the experiment. During PASEF MSMS scanning, the collision energy was ramped linearly as a function of the mobility from 59 eV at 1/K0 = 1.6 Vs/cm2 to 20 eV at 1/K0 = 0.6 Vs/cm2. Raw data of DIA were processed and searched against a protein database using the

Spectronaut 19 (Biognosys AG, Switzerland) with default settings. Human protein database was downloaded from UniProt (homo_sapiens, 20608 entries, version 2024). Qvalue (FDR) cutoff were set as 1% at the precursor, peptide, and protein level. Peptides which passed the 1% Qvalue cutoff were used to calculate the major group quantities with the MaxLFQ method.

MS data analysis

To investigate the varying interaction capabilities between the WT and mutant MED27, the MS data were analyzed using the Significance Analysis of Interactome (SAINT) tool [13]. Our proteomics data captured approximately 71,000 unique peptides mapping to 5,516 proteins. All bait-prey interactions with an average probability cutoff score of 0.9 Saint score and nuclear localization were considered as high-confidence interacting proteins (HCIPs) for further analysis on putative specific interactions. In the WT group, there were 1,155 proteins exhibited a SAINT score exceeding 0.9, whereas the mutant group illustrated 1,210 such proteins. Among these, 664 proteins in the WT group were nuclear proteins and classified as HCIPs, while the mutant group included 650 HCIPs (Table S2 Sheet 1 and 2). Subsequently, differentially interacting proteins between the WT and the mutant group were identified based on the cutoff of $|\log_2$FoldChange (FC_B)$|>1.5$ (total of 41 proteins as shown in Table S2 Sheet 3). Proteins with $\log_2$FC_B(mutant/wt)> 1.5 were classified as mutant preferred interactors, whereas proteins with $\log_2$FC_B(mutant/wt) < -1.5 were classified as WT preferred interactors [14].

Further disease association analysis was performed using DisGeNET, a platform integrating information on gene–disease association (GDA) and variant–disease association (VDA) to annotate potential disease relevance of the selected genes. A list of candidate genes with $|\log_2 FC|>2SD$ (comparing mutant against WT, SD=1.23, Table S2 Sheet 4) were subjected to the platform analysis and the top 10 enriched disease clusters were identified [15, 16].

Mouse models

The *Med27* gene is located on chromosome 2. *Med27* knockout (KO) mice and *Med27*$^{f/f}$ mice were purchased from Cyagen. To achieve central nervous system-specific deletion and Purkinje cell-specific deletion of *Med27*, *Med27*$^{f/f}$ mice were crossed with Nestin-Cre (B6.Cg-Tg(Nes-cre)1Kln/J, RRID:IMSR_JAX:003771) and Pcp2-Cre (C57BL/6N, C001010, Cyagen) mice, respectively, genotyped and bred for desired genotypes. All animal experiments were approved by the Animal Experimentation Ethics Committee (AEEC) at

CUHK and all the experimental procedures were conducted in the accordance of AEEC guidelines at CUHK.

Mouse embryo collection and genotyping

Med27 heterozygous KO females (8 to 12 weeks old) were mated with *Med27* KO heterozygous males, with the presence of vaginal plugs marking the date of fertilization (E0.5). Embryos were collected into individual tubes either by dissection or by flushing from the uteri of female mice. Collected embryos were lysed in lysis buffer (100 mM Tris-HCl pH 8.3, 100 mM KCl, 0.02% gelatin, 0.45% Tween 20, 60 μg/mL yeast tRNA) supplemented with Proteinase K. The lysates were digested at 56°C for 10 minutes, followed by a heat inactivation step at 95°C for 10 minutes. The genotypes of the embryos were determined by PCR in a 20 μL reaction volume containing 2 μL DNA template, 10 μL 2X Rapid Tag Master Mix (Vazyme), and 0.4 μM of primers (see Table S3 for primer sequences). PCR amplification was performed under the following condition: 95°C for 5 minutes followed by 35 cycles at 95°C for 30s, 62 °C for 30s, and 72°C for 15s, followed by a final extension at 72°C for 5 minutes.

Paraffin section and cryosection

Mice were anesthetized and perfused with cooled 1x phosphate buffered saline (PBS, pH=7.4) at a flow rate of 70 rpm flowed by perfusion with 4% paraformaldehyde (PFA) in 1xPBS. Whole brains were carefully extracted and post-fixed with 4% PFA in PBS for 48 hours at 4°C. Post-fixation, the brains were either transferred to 70% ethanol at 4°C for dehydration and paraffinization using a tissue processor (Epredia Excelsior AS Tissue Processor) or transferred to 15%-30% sucrose in 1xPBS for dehydration and cryoprotection. For paraffin section, the brain tissues were embedded in paraffin wax (Epredia HistoStar Embedding center). The paraffin blocks were sagittally sectioned at a thickness of 5-7 μm using a microtome (Leica RM2335 Rotary Microtome). For cryosection, the brain tissues were embedded in Optimal Cutting Temperature (OCT) compound (CryoGlue, SLEE medical GmbH). Cryosections were cut at a thickness of 10 μm using a cryotome (Epredia Cryostar NX70 Cryostat).

IF staining in mice

All paraffin sections were first processed with dewaxing protocol using MEDITE TST 44 Automatic Slide Stainer, followed by antigen retrieval using the Thermo PT Module Antigen Retiever. Both paraffin sections and cryosections were washed with 1xPBS and blocked with 5% goat serum and 0.3% Triton X-100. The sections were then incubated overnight at 4°C with primary antibodies (Table S3). The following day, the sections were incubated with

secondary antibodies (Table S3), counterstained with DAPI and mounted using Fluoromount-G™ Mounting Medium (Invitrogen, 495802). TUNEL staining was performed following the protocol provided with the DeadEnd™ Fluorometric TUNEL System Kit (Promega #G3250). Imaging of the slides was performed using a confocal microscope (Leica TCS SP8 Inverted Confocal Microscope). Images were analyzed and quantified using Image J software (version 13.0.0/1.53k). Representative images were taken in the same lobule of the cerebellum and adjusted for brightness and contrast to ensure consistent background intensity.

Total RNA isolation in mice

Mice were anesthetized and perfused with cooled 1x PBS (pH=7.4) at a flow rate of 70 rpm to clear blood. After perfusion, cerebellum was extracted, and the total RNA was isolated using the TriRNA Pure kit (Geneaid, TRPD200). RNA concentration and quality were quantified using a NanoDrop spectrophotometer (Thermo Fisher).

Protein extraction and western blot of cerebellum tissue

Cerebellum tissues were lysed using a lysis buffer containing 150mM NaCl, 1% Triton X-100, 25mM Tris-HCl, 5mM EDTA, 10% Glycerol, and 1x protease inhibitor (Selleckchem, B14001). Protein concentrations were measured using the Pierce BCA Protein Assay Kit (Thermo Fisher Scientific). Antibodies used for detection are listed in the Table S3. Membranes were imaged using the Amersham Cytiva Image Quant 800 Imaging System and data were analyzed using Image J software (version 13.0.0/1.53k).

Behavioral tests

All behavioral tests were performed during the 12-hour light cycle. Mice used in the experiments were aged- matched, and littermate controls were included. During testing, all mice had access to food and water.

Beam-walk assay

The beam-walk test was used to assess motor coordination and balance. A narrow rectangular beam (1 cm wide, 1 meter long, and positioned horizontally 20 cm above the surface) was supported by boxes at both ends. One end of the beam was attached to an enclosed dark box to house the mouse after crossing. In this test, the mouse was placed at the starting point of the beam, and the following parameters were recorded: number of paw slips, misplacement time (the duration the paws remained off the beam following a slip), and total crossing time. Cameras positioned on both sides recorded these events. To ensure optimal performance, each mouse underwent at least three training sessions before the actual test to minimize latency and avoid directional turns.

Footprint analysis

The footprint test was used to measure forelimb and hindlimb locomotion. Edible oil-based colors were applied to the forepaws (blue) and hindpaws (red). The mouse then walked along a 5-cm-wide, 60-cm-long white paper placed inside an open-air corridor, which led to an enclosed dark box at the end. Each mouse underwent two trials, and three consecutive steps per trial were analyzed to determine the average step length and stride length between the forepaws and hindpaws.

Rotarod test

To evaluate motor coordination and motor learning abilities, a rotarod test was conducted using a rotarod device (PanLab LE 8205). Mice were habituated to the device the day before testing by completing five 90-second trials at a constant speed of 4 rpm. On the test day, the mice ran on the device as it accelerated from 4 to 40 rpm over 5 minutes. The primary outcomes were measured: latency to fall (the time taken before the mouse fell) and rotation speed at the time of falling. The average results and the learning curve from five trials were recorded. Each trial was followed by a mandatory 15-minute rest period to prevent fatigue.

Visium HD Spatial transcriptomics

Tissue processing

Mice were anesthetized and perfused with cooled 1xPBS at a flow rate of 70 rpm to clear blood and preserve tissue integrity. After perfusion, the cerebellum was carefully extracted, embedded in OCT compound and quickly frozen at -80 °C. The frozen cerebellum was then sectioned into 10 µm slices in sagittal plane using a cryostat (Leica CM1950). These sections were stored at -80 °C until further processing to prevent any tissue degradation. All procedures were followed according to the manufacturer's instructions (CG000685, Visium HD Spatial Gene Expression Reagent Kits, 10x Genomics) and carried out in the Single Cell & Spatial Omics Core laboratory of the School of Biomedical Sciences at CUHK.

Data analysis

Illumina's raw sequencing data from *Med27*$^{f/f}$ (WT) and *Med27*$^{f/f}$;*Pcp2*^{Cre} (Mutant) samples were demultiplexed into FASTQ files by Space Ranger 'mkfastq' pipeline (10x Genomics v7.1). Using the Visium HD Manual Alignment tools provided by Loupe Browser (10x Genomics v8.0.0), we manually aligned fiducial markers to tissue sections captured by brightfield microscope. Space Ranger 'count' pipeline (10x Genomics v3.1.1) was used to generate spatial feature counts of tissue selection. Following analyses of datasets were performed using pipelines in the Seurat package (v5.1.0) in R. Reads mapped to mitochondrial genes (*mt-Nd1, mt-Nd2, mt-Co1, mt-Co2, mt-Atp8, mt-Atp6, mt-Co3, mt-Nd3, mt-Nd4l, mt-Nd4, mt-Nd5, mt-Nd6, mt-Cytb*) were excluded. Spots with very few or extremely

high counts were filtered out in each dataset based on the violin plot of log2-transformed UMIs (nCounts) (for WT nCount>=30 and nCount <=2048; for Mutant nCount>=40 and nCount <=2048). Each dataset was then normalized using the standard log-normalization method with a scale factor of median UMI per 8um bin in Seurat. Variable features were identified by 'FindVariableFeatures' function with default parameters, followed by scaling data. Principal Component Analysis (PCA) coordinates were generated by 'RunPCA' before data integration by Harmony. Subsequently, the top 30 principal components were used to generate UMAP embedding, which was used to define cell-type clusters with the 'FindNeighbors' and 'FindClusters' (resolution = 0.45) functions. Cluster-specific marker genes were identified using the 'FindAllMarkers' (min.pct=0.25, only.pos = TRUE) function. The top 50 genes per cluster were used to guide the annotation of the clusters with H&E stained histological images and with referenced to the Allen Brain Atlas, while gene expressions were visualized in the spatial localization upon the histology images. DEGs between WT and Mutant samples in each cluster were identified using the 'FindMarker' function with Wilcoxon Rank Sum test. Genes were considered significant based on adjusted *P* value < 0.05 and averaged log2fold change>0.58 (absolute value). Feature plots, violin plots, and dot plots were generated by Seurat.

RCTD deconvolution of Visium HD data

To validate the annotated cell types from unsupervised clustering, Robust Cell Type Decomposition (RCTD) deconvolution analysis was performed using a single-cell RNA-seq (scRNA-seq) cerebellum dataset [17]. The reference scRNA-seq dataset was reduced to 10,000 cells per cell type using RCTD 'Reference' default function in R package spacexr v2.2.1. RCTD was run to deconvolute the whole tissue section in doublet mode and then projected these annotations to the Seurat object to perform spatial visualization.

***Ex vivo* MRI**

MR facility (MR Solutions Inc.) is in the Shanghai Fraternity Association Research Services Centre at CUHK. Embryos were harvested and genotyped, fixed with 4% PFA overnight, then were embedded in 1% agarose and imaged in 7.0T mouse head coil.

Statistical analysis

Statistical tests were performed based on the experimental design. For simple comparisons, two-tailed Student's t-test was used, while one-way or two-way ANOVAs with multiple comparisons were applied for multi-group caparisons. Analyses were conducted using GraphPad Prism (version 9.4.1).

Supplementary references

1. Komor, A.C., Y.B. Kim, M.S. Packer, J.A. Zuris, and D.R. Liu, *Programmable editing of a target base in genomic DNA without double-stranded DNA cleavage.* Nature, 2016. **533**(7603): p. 420-4.
2. Servant, N., N. Varoquaux, B.R. Lajoie, E. Viara, C.J. Chen, J.P. Vert, E. Heard, J. Dekker, and E. Barillot, *HiC-Pro: an optimized and flexible pipeline for Hi-C data processing.* Genome Biol, 2015. **16**: p. 259.
3. Langmead, B. and S.L. Salzberg, *Fast gapped-read alignment with Bowtie 2.* Nat Methods, 2012. **9**(4): p. 357-9.
4. Imakaev, M., G. Fudenberg, R.P. McCord, N. Naumova, A. Goloborodko, B.R. Lajoie, J. Dekker, and L.A. Mirny, *Iterative correction of Hi-C data reveals hallmarks of chromosome organization.* Nat Methods, 2012. **9**(10): p. 999-1003.
5. Durand, N.C., M.S. Shamim, I. Machol, S.S. Rao, M.H. Huntley, E.S. Lander, and E.L. Aiden, *Juicer Provides a One-Click System for Analyzing Loop-Resolution Hi-C Experiments.* Cell Syst, 2016. **3**(1): p. 95-8.
6. Rowley, M.J., A. Poulet, M.H. Nichols, B.J. Bixler, A.L. Sanborn, E.A. Brouhard, K. Hermetz, H. Linsenbaum, G. Csankovszki, E. Lieberman Aiden, and V.G. Corces, *Analysis of Hi-C data using SIP effectively identifies loops in organisms from C. elegans to mammals.* Genome Res, 2020. **30**(3): p. 447-458.
7. Roayaei Ardakany, A., H.T. Gezer, S. Lonardi, and F. Ay, *Mustache: multi-scale detection of chromatin loops from Hi-C and Micro-C maps using scale-space representation.* Genome Biol, 2020. **21**(1): p. 256.
8. Abdennur, N. and L.A. Mirny, *Cooler: scalable storage for Hi-C data and other genomically labeled arrays.* Bioinformatics, 2020. **36**(1): p. 311-316.
9. Flyamer, I.M., R.S. Illingworth, and W.A. Bickmore, *Coolpup.py: versatile pile-up analysis of Hi-C data.* Bioinformatics, 2020. **36**(10): p. 2980-2985.
10. Davis, E., *hictoolsr: An R Package for Hi-C Data Analysis.* 2022.
11. Love, M.I., W. Huber, and S. Anders, *Moderated estimation of fold change and dispersion for RNA-seq data with DESeq2.* Genome Biol, 2014. **15**(12): p. 550.
12. Bian, W., H. Jiang, S. Feng, J. Chen, W. Wang, and X. Li, *Protocol for establishing a protein-protein interaction network using tandem affinity purification followed by mass spectrometry in mammalian cells.* STAR Protoc, 2022. **3**(3): p. 101569.
13. Kaushal, P., M.R. Ummadi, G.M. Jang, Y. Delgado, S.K. Makanani, K. Alba, D.M. Winters, S.F. Blanc, J. Xu, B. Polacco, Y. Zhou, E. Stevenson, M. Eckhardt, L. Zuliani-Alvarez, R. Kaake, D.L. Swaney, N.J. Krogan, and M. Bouhaddou, *Protocol for mapping differential protein-protein interaction networks using affinity purification-mass spectrometry.* STAR Protoc, 2024. **5**(4): p. 103286.
14. Bian, W., H. Jiang, L. Yao, W. Hao, L. Wu, and X. Li, *A spatially defined human Notch receptor interaction network reveals Notch intracellular storage and Ataxin-2-mediated fast recycling.* Cell Rep, 2023. **42**(7): p. 112819.
15. Pinero, J., A. Bravo, N. Queralt-Rosinach, A. Gutierrez-Sacristan, J. Deu-Pons, E. Centeno, J. Garcia-Garcia, F. Sanz, and L.I. Furlong, *DisGeNET: a comprehensive platform integrating information on human disease-associated genes and variants.* Nucleic Acids Res, 2017. **45**(D1): p. D833-D839.
16. Zhou, X., Y.K. Lee, X. Li, H. Kim, C. Sanchez-Priego, X. Han, H. Tan, S. Zhou, Y. Fu, K. Purtell, Q. Wang, G.R. Holstein, B. Tang, J. Peng, N. Yang, and Z. Yue, *Integrated proteomics reveals autophagy landscape and an autophagy receptor controlling PKA-RI complex homeostasis in neurons.* Nat Commun, 2024. **15**(1): p. 3113.

17. Kozareva, V., C. Martin, T. Osorno, S. Rudolph, C. Guo, C. Vanderburg, N. Nadaf, A. Regev, W.G. Regehr, and E. Macosko, *A transcriptomic atlas of mouse cerebellar cortex comprehensively defines cell types.* Nature, 2021. **598**(7879): p. 214-219.
